# Minocycline Abrogates Individual Differences in Nerve Injury-Evoked Affective Disturbances in Male Rats and Prevents Associated Supraspinal Neuroinflammation

**DOI:** 10.1007/s11481-024-10132-y

**Published:** 2024-06-15

**Authors:** Jayden A. O’Brien, Paul J. Austin

**Affiliations:** Brain and Mind Centre, School of Medical Sciences, Faculty of Medicine and Health, The University of Sydney, Sydney, NSW Australia

**Keywords:** Anhedonia, Chronic constriction injury, Depression-like behaviour, Hippocampus, Pain-related disability, Radial arm maze

## Abstract

Chronic neuropathic pain precipitates a complex range of affective and behavioural disturbances that differ markedly between individuals. While the reasons for differences in pain-related disability are not well understood, supraspinal neuroimmune interactions are implicated. Minocycline has antidepressant effects in humans and attenuates affective disturbances in rodent models of pain, and acts by reducing neuroinflammation in both the spinal cord and brain. Previous studies, however, tend not to investigate how minocycline modulates individual affective responses to nerve injury, or rely on non-naturalistic behavioural paradigms that fail to capture the complexity of rodent behaviour. We investigated the development and resolution of pain-related affective disturbances in nerve-injured male rats by measuring multiple spontaneous ethological endpoints on a longitudinal naturalistic foraging paradigm, and the effect of chronic oral minocycline administration on these changes. Disrupted foraging behaviours appeared in 22% of nerve-injured rats – termed ‘affected’ rats – and were present at day 14 but partially resolved by day 21 post-injury. Minocycline completely prevented the emergence of an affected subgroup while only partly attenuating mechanical allodynia, dissociating the relationship between pain and affect. This was associated with a lasting downregulation of ΔFosB expression in ventral hippocampal neurons at day 21 post-injury. Markers of microglia-mediated neuroinflammation were not present by day 21, however proinflammatory microglial polarisation was apparent in the medial prefrontal cortex of *affected* rats and not in CCI minocycline rats. Individual differences in affective disturbances following nerve injury are therefore temporally related to altered microglial morphology and hippocampal neuronal activation, and are abrogated by minocycline.

**Graphical Abstract:**

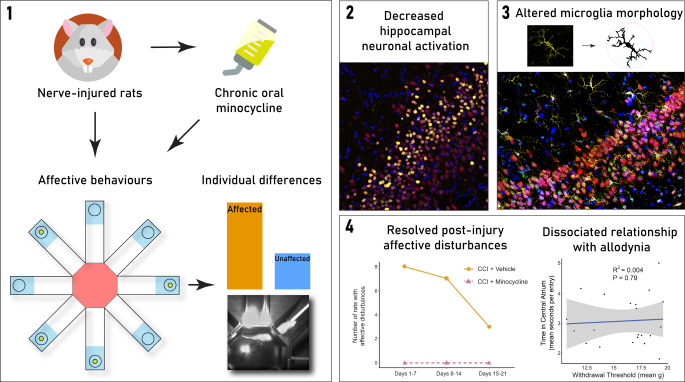

**Supplementary Information:**

The online version contains supplementary material available at 10.1007/s11481-024-10132-y.

## Introduction

Chronic neuropathic pain profoundly impacts the quality of life of affected people. However, the degree to which an individual is disabled by their pain can vary remarkably (Bay-Nielsen et al. 2001; Kehlet et al. 2006; Müller et al. 2017). There is therefore great interest in uncovering the reasons for individual differences in disability in response to nerve injury (Chou and Shekelle 2010; Tanguay-Sabourin et al. 2023). Increased evoked nociceptive hypersensitivity does not fully explain these individual differences (Bagnato et al. 2015; Montoro and Galvez-Sánchez 2022), and instead psychosocial factors such as coping style, pain catastrophising, and self-efficacy appear to mediate increased self-reported pain intensity levels in chronic pain patients with high levels of disability (Arnstein et al. 1999; Pincus et al. 2002; Wood et al. 2016; Lazaridou et al. 2022). Depression and anxiety are common disabling comorbidities, affecting between one and two-thirds of chronic pain patients, though there is again individual variation in their incidence and severity mediated by emotional coping style and other factors (Cherif et al. 2020; Kec et al. 2022; Damci et al. 2022).

The dynamics and potential neuroimmune substrates of individual differences in affective and motivational disturbance following nerve injury have been previously explored in rodent models (Walker et al. 2014; Fasick et al. 2015; Austin and Fiore 2019). For example, a subgroup of rats after spared nerve injury emerges with comorbid depressive-like behaviours as measured by the sucrose preference and forced swim tests (Xie et al. 2017). While these tests remain popular choices for investigating affective disturbances in rodents, they capture a limited component of possible affective pain behaviours and face increasing scrutiny of their applicability (Molendijk and de Kloet 2022; Markov 2022). Instead, multimodal, longitudinal, and ethological approaches that measure complex and spontaneous behaviours are increasingly favourable for their ability to detect nuanced behavioural pain signatures (Leite-Almeida et al. 2022; Bohic et al. 2023).

The modified radial arm maze paradigm allows for the measurement of complex behavioural patterns within a naturalistic foraging task, and distinguishes a subgroup of rats with disrupted foraging behaviours after nerve injury (Fiore and Austin 2018, 2019). These rats display a distinct behavioural phenotype of decreased motivation and exploratory behaviours, increased risk assessment and withdrawal, and reduced locomotion. This phenotype is associated with concomitant glial and neuronal activation changes in anatomically specific areas of the ventral hippocampus and medial prefrontal cortex, such as increases in pro-inflammatory mediators interleukin-1 beta (IL-1β), phosphorylated p38 mitogen-activated protein kinase (pp38 MAPK), interleukin-6 (IL-6), and chemokine ligand 2 (CCL2/MCP-1); an increase in neuronal activation marker FosB/ΔFosB; a decrease in brain-derived neurotrophic factor (BDNF); and deramification of microglia to a more reactive, pro-inflammatory morphology. This neuroimmune signature does not arise in nerve-injured rats without disrupted foraging behaviours despite a similar level of evoked mechanical hypersensitivity (Fiore and Austin 2019).

Minocycline elicits neuroprotective effects in both neuropathic pain and clinical depression (Rosenblat and McIntyre 2018; Cai et al. 2020; Shin et al. 2021), at least in part by promoting anti-inflammatory microglial polarisation (Lin et al. 2001). Minocycline has therefore been used as a pro-inflammatory microglia inhibitor to investigate microglial involvement in models of nerve injury and associated affective disturbances (Raghavendra et al. 2003). However, most studies to date have investigated affective disturbances at the group level, and those that have studied individual differences in such behaviours have typically relied on a limited set of measures to define the phenotype that may not capture a holistic view of the manner in which nerve injury disrupts affective responding. It is therefore not known whether the neuroinflammatory signature specific to individuals with complex behavioural disturbances following nerve injury can be attenuated by minocycline.

We aimed to determine whether individual differences in complex affective disturbances of nerve-injured rats could be normalised via the pharmacological repolarisation of microglia by chronic minocycline administration. We hypothesised that minocycline would both attenuate mechanical allodynia and reduce the proportion of nerve-injured rats expressing highly disrupted foraging behaviours, and that this would be associated with normalised supraspinal glial and neuronal activation.

## Materials and Methods

### Ethics and Subjects

Naïve outbred male Sprague-Dawley rats (*n* = 74; Animal Resource Centre, Perth, Australia) were aged 6 weeks and weighed 203 ± 7.0 g on arrival. Methods are reported according to the ARRIVE 2.0 guidelines (Percie du Sert et al. 2020). Male rats were used due to the previous validation of the radial maze affective disability paradigm in male rats only.

### Experimental Design

**Fig. 1:**
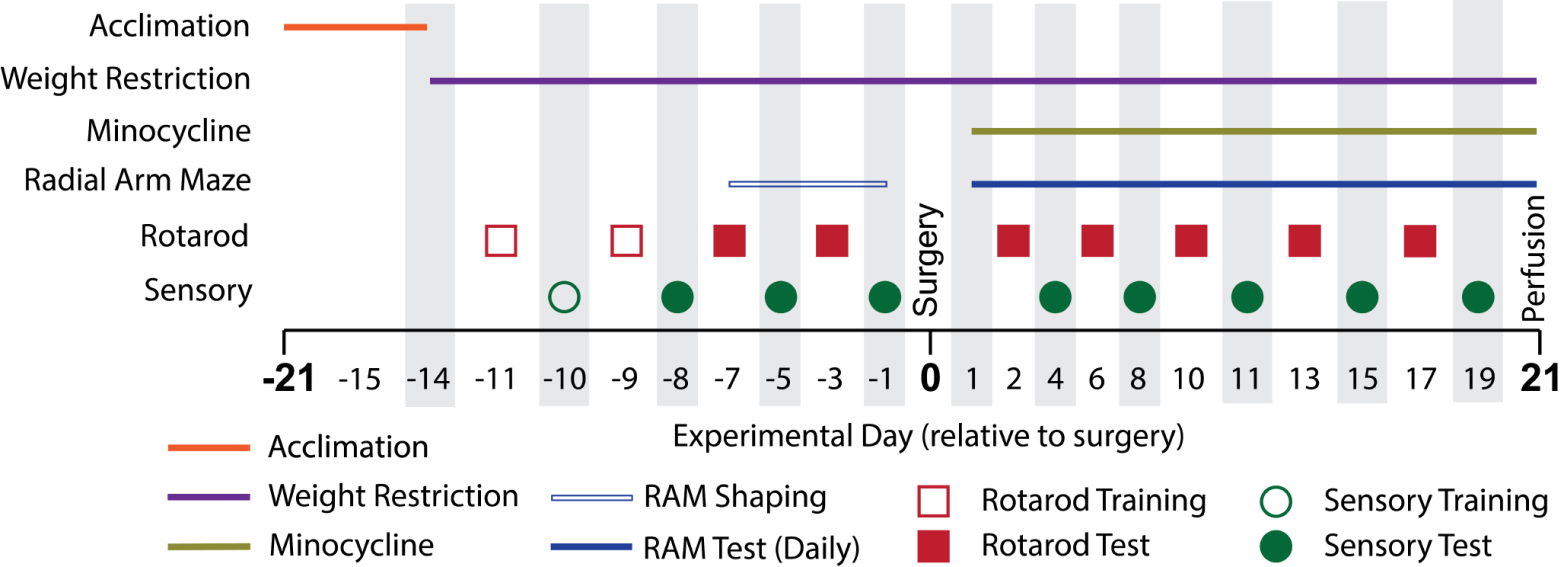
Experimental timeline for rat behaviour and interventions. The coloured bars and symbols indicate the experimental days on which the listed experimental conditions and paradigms took place. For the seven days following acclimation, all rats began weight restriction to 90% of their free-feeding weight to maximise motivation for sucrose pellet seeking, then underwent 7 days of daily shaping on the radial arm maze (RAM) task. Rats underwent chronic constriction injury (CCI) or sham surgery, and a subgroup of each were administered chronic minocycline from the first day following surgery (see *2.3*) or received vehicle only (tap water). A total of 21 days of behavioural post-testing followed, which included daily RAM testing and five days each of motor and sensory testing as indicated in the figure. Rats were perfused following RAM testing on day 21

Rats were singly housed in open-topped cages to allow rats to be in auditory and olfactory contact. A 12:12 reverse light-dark cycle was used. Rats were provided with nesting material and environmental enrichment. Rats acclimated to their housing conditions for seven days after arrival during which standard chow was available *ad libitum*. Cage position and experimental group were randomly assigned. Rats were all tested, underwent surgery, and were perfused in cage order, and all experimental procedures were blocked to ensure equal representation of experimental groups. Due to impracticalities, during husbandry the experimenter was only blinded to experimental groups prior to surgery. The experimental timeline is outlined in Fig. 1.

The optimal sample size, given a predicted effect size (*f*) of 0.8 and power (1-β) of 0.8, was calculated *a priori* to be *n* = 5 per group in G*Power 3.0. This was based on the primary outcome measure, the time spent in the central atrium of the radial maze, a highly consistent indicator of other measures of disrupted foraging behaviours in this paradigm (Fiore and Austin 2018). However, since only 20–40% of nerve-injured rats will go on to display affective disturbances (Fiore and Austin 2018, 2019), we included many more rats in the CCI vehicle (*n* = 37) and CCI minocycline (*n* = 21) groups than the sham vehicle (*n* = 9) and sham minocycline (*n* = 7) groups to allow this behaviourally distinct subgroup to develop.

### Chronic Constriction Injury and Minocycline Administration

Chronic constriction injury (CCI) of the sciatic nerve was conducted as previously described (Bennett and Xie 1988; see Fiore and Austin 2018 for a detailed protocol). The sham group had the sciatic nerve exposed under the same surgical conditions, but CCI was not performed. Sham and CCI minocycline groups were administered 40 mg/kg/day minocycline hydrochloride (M344800, Toronto Research Chemicals, Canada) dissolved daily in their drinking water from 24 h following surgery. This was chosen based on a previously published protocol (Hinwood et al. 2012).

### Quantitative Motor and Sensory Testing

Motor coordination was assessed using the rat rotarod (Ugo Basile, Varese, Italy), which has been used in CCI models previously to investigate the effect of injury on motor function (Hara et al. 2022). Rats were placed on the apparatus as it rotated at 5 rpm accelerating to 35 rpm over 3 min. The latency to fall off the apparatus (in seconds) was taken as the average of three trials. The placement order of rats on the rotarod was counterbalanced between trials.

Evoked mechanical hypersensitivity was measured using a dynamic plantar aesthesiometer (Ugo Basile, Varese, Italy). The filament was placed against the plantar surface of each hindpaw and allowed to ramp from 0 g at 1 g/s until the rat withdrew the paw or 50 g was reached. A trial was recorded if the rat was determined to have withdrawn the paw as a direct consequence of the mechanical stimulus. The withdrawal threshold (in grams) was taken as the mean of five technical replicates for each hindpaw on the days marked in Fig. 1.

### Affective and Spatial Memory Behaviours

**Fig. 2:**
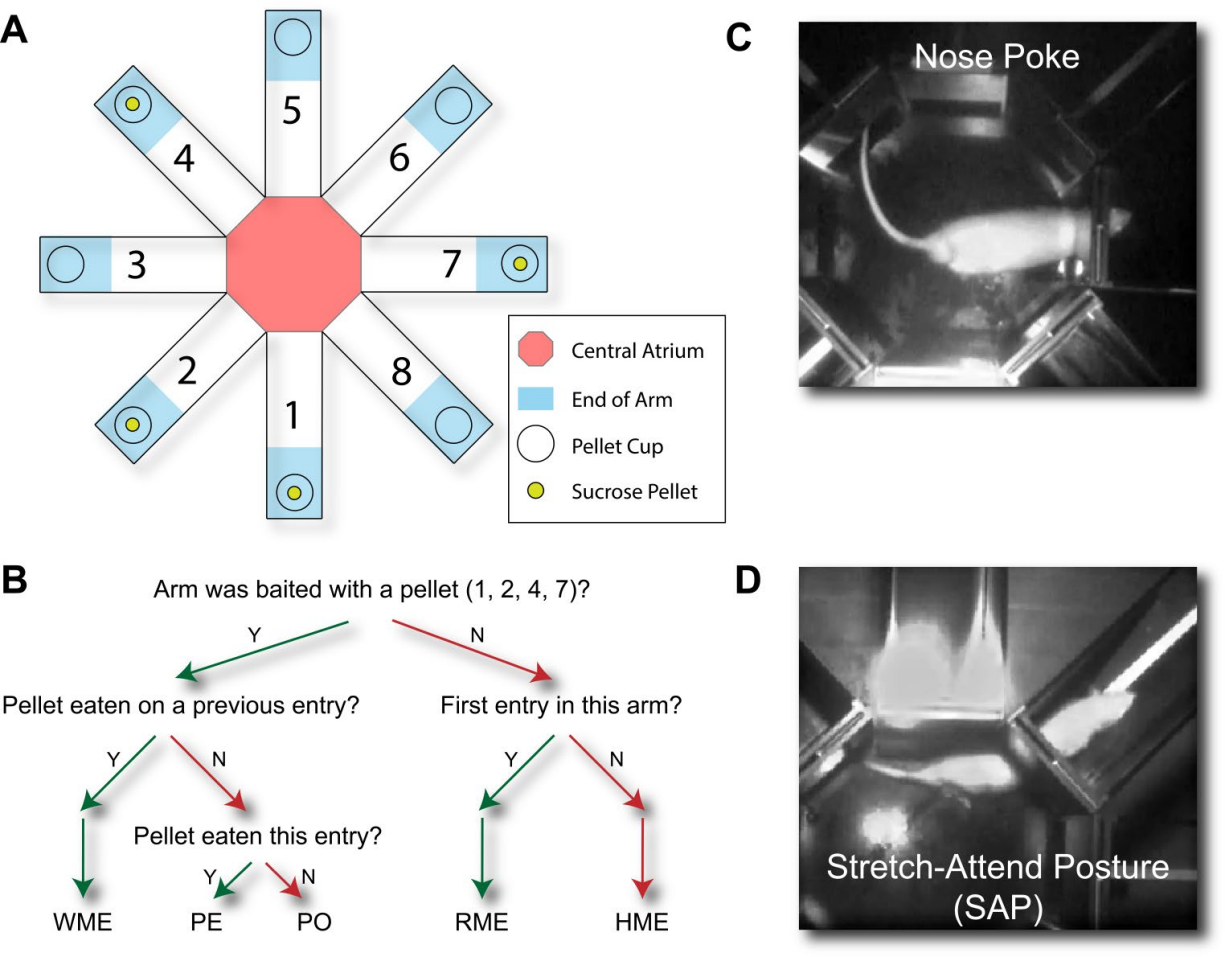
Description of the daily radial arm maze task for evaluating affective and cognitive changes following nerve injury. (**A**) Schematic of the radial arm maze apparatus and experimental setup. The apparatus consists of an elevated, open-air maze with a central walled octagonal chamber – the central atrium – from which extend eight arms. The walls of the arms decrease in height distally from the central atrium. A sunken cup, whose contents were not visible from the central atrium, was located at the end of each arm. A sucrose pellet was placed in the cup at the end of arms 1, 2, 4, and 7. (**B**) Flow diagram representing the logic of the definition of each arm entry performed by a rat in the radial arm maze. WME: working memory error; PE: pellet eaten; PO: pellet omission; RME: reference memory error; HME: hybrid memory error (counted as both a working and reference memory error). (**C**) Example of a nose poke: the rat remains in the central atrium while placing its snout into an arm before withdrawing. (**D**) Example of a stretch-attend posture (SAP): the rat keeps its hindpaws in the central atrium while extending its body into an arm, often paired with investigative whisking, before withdrawing

To assess affective disturbances following surgery, deep behavioural phenotyping of affective behaviours was performed by testing rats once each day for 21 days using the radial arm maze foraging paradigm (modified from Olton and Werz 1978) as reported previously (Fiore and Austin 2018, 2019). Rats underwent seven days of shaping with multiple sucrose pellets placed in each of the arms (Bio-Serv Dustless Precision Pellets, cat no. F0021) to motivate exploration. For all experimental post-surgery trials, only the cups of arms 1, 2, 4, and 7 were baited to allow assessment of memory performance (Fig. 2a).

Rats were placed singly in the central atrium of the maze and allowed to explore for five minutes. Video recordings were coded for affective- and memory-related behaviours using Solomon Coder (version 19.08.02, solomon.andraspeter.com). Each entry into an arm of the maze was given one of the following definitions: pellet eaten (PE), pellet omission (PO), working memory error (WME), reference memory error (RME), and hybrid memory error (HME; Fig. 2b). Affective behaviours, coded by either duration or frequency, included time spent in the central atrium (TICA), time spent in the end of arms (TIEOA), grooming, rearing, climbing, nose pokes (Fig. 2c), and stretch-attend postures (SAPs; Fig. 2d). These endpoints are well-attested in similar behavioural paradigms including the open field and elevated plus maze tests (Mikics et al. 2005), and are summarised in Table 1.

**Table 1:** Description and classification of affective-motivational endpoints measured daily in the radial arm maze paradigm

Parameter	Description	Classification
Time in central atrium (TICA)	Time spent in the central chamber of the maze, withdrawn from the foraging task. The key measure used to define behavioural subgroups.	Withdrawal; anhedonia-like behaviour
Time in end of arm (TIEOA)	Time spent in the distal third of an arm of the maze.	Exploratory behaviour
Nose pokes	Frequency with which the rat, while in the central atrium, poked its snout into an arm, then withdrew back to the atrium. Also referred to as head dipping.	Risk assessment
Stretch-attend postures (SAPs)	Frequency with which the rat, with its hindpaws in the central atrium, stretched its body out to place its forepaws in an arm of the maze, perhaps engaged in investigative whisking, and then withdrew back to the atrium.	Risk assessment
Grooming	Time spent engaging in ritualistic grooming behaviour.	Anxiety-like behaviour
Rearing	Time spent on its hindpaws with one or more forepaws against the walls of the central atrium or the proximal two-thirds of an arm.	Exploratory behaviour
Climbing	Time spent climbing on top of the apparatus.	Exploratory behaviour

Rats were categorised into groups based on affective phenotype in the radial maze. A CCI rat, either vehicle- or minocycline-administered, was classified as *affected* if, for at least three days between each of days 1–6 and 7–12 post-surgery, the time they spent in the central atrium of the maze normalised to the number of arm entries was greater than three standard deviations above the mean of the relevant sham group on that day. Rats not meeting this criterion were classified as *unaffected*.

### Tissue Processing and Immunofluorescence Staining

On post-surgery day 21, following radial arm maze testing, rats were deeply anaesthetised with 120 mg/kg i.p. sodium pentobarbital (Lethabarb) and transcardially perfused with 0.9% w/v cold saline followed by 4% w/v cold paraformaldehyde solution, pH 9.6. The brain was extracted and post-fixed for 1 h. Tissue was then transferred to 30% w/v sucrose solution in phosphate buffered saline, pH 7.4 (PBS, with 0.05% w/v sodium azide). Brains were cryosectioned and transferred to antifreeze for long-term storage at -20 °C. Sciatic nerves were inspected to verify the appropriate placement and tightness of ligatures.

Double- or triple-label immunofluorescence staining was performed on free-floating sections (*n* = 6 per group). All steps were carried out on an orbital shaker in a volume of 1.5mL per series, with three washes in PBS performed between all staining steps, and incubations performed at room temperature unless otherwise specified. Sections underwent heat-induced epitope retrieval in 0.01 M sodium citrate buffer, pH 6, with 0.05% v/v Tween 20 at 80 °C for 30 min. Sections were then incubated in 0.1% w/v sodium borohydride (NaBH_4_) in PBS for 30 min to reduce autofluorescence, permeabilised with 0.3% v/v Triton X-100 in PBS for 30 min, and blocked in 10% v/v normal horse serum (NHS) in 0.3% v/v Triton X-100 in PBS for 30 min. For experiments using tertiary streptavidin amplification, additional avidin (0.05 mg/mL in PBS for 30 min) and biotin (0.1 mg/mL in PBS for 30 min) incubations blocked endogenous biotin.

Sections were then incubated in the first primary antibody solution (one of: 1:300 rabbit anti-rat BDNF [Abcam ab108319, RRID: AB_10862052], 48 h; 1:750 rabbit anti-rat CD206 [Abcam ab64693, RRID: AB_1523910], 24 h; 1:400 rabbit anti-rat IL-1β [Abcam ab9722, RRID: AB_308765], 72 h; 1:300 rabbit anti-rat phospho-p38 MAPK [Cell Signaling Technologies #4511, RRID: AB_2139682], 72 h; 1:300 rabbit anti-rat FosB/ΔFosB [Invitrogen MA5-15056, RRID: AB_10983364], 72 h) in 0.15% v/v Triton X-100, and 2% NHS in PBS at 4 °C (see Supplementary Table A1 for details). Sections were incubated with biotin- or Alexa series fluorophore-conjugated fab fragment secondary antibodies produced in donkey (Jackson ImmunoResearch) in 2% NHS in PBS for 3 h. FosB and pp38 MAPK underwent fluorophore-conjugated streptavidin amplification in PBS for 2 h. The primary antibody was then further incubated in 1:100 unconjugated fab fragments (Jackson ImmunoResearch, cat no. 711-007-003, RRID: AB_2340587) to block crossreactivity with the second primary antibody raised in the same species. Second primary and secondary incubations were performed as before (one of: 1:1000 rabbit anti-rat IBA1 [Abcam ab178846, RRID: AB_2636859]; 1:2000 rabbit anti-rat GFAP [Abcam ab7260, RRID: AB_305808]; and 1:500 mouse anti-rat NeuN [Millipore MAB377, RRID: AB_2298772], 48 h), followed by nuclear staining with DAPI dilactate in PBS for 30 min. Sections were mounted on gelatinised slides and coverslipped with Prolong Gold anti-fade mounting medium (Invitrogen #P36934). Single-label and negative controls were performed to rule out cross-reactivity with other included primary antibodies and non-specific binding of the secondary antibodies.

### Confocal Microscopy and Image Processing

Fluorescence photomicrographs were acquired across up to 46 regions of interest across the hippocampus and medial prefrontal cortex (mPFC), regions associated with affective responses, and the ventroposterior lateral (VPL) thalamus to assess a supraspinal region associated with ascending nociceptive input. Bilateral dorsal, intermediate, ventral, and ventral pole dentate gyrus, cornu Ammonis (CA)3 and CA1; rostral, mid, and caudal cingulate cortex, prelimbic cortex, and infralimbic cortex; dorsolateral and ventromedial VPL thalamus; and zona incerta were defined according to the rat brain atlas (Paxinos and Watson 2004). Images were taken using a confocal microscope (Nikon C2+) as multichannel z-stacks with a 1 μm step size by an experimenter blinded to experimental group. All parameters were kept constant between subjects (see Supplementary Table A2). Single-channel control images were taken of a randomly selected slide from each staining run to rule out bleed-through between channels.

The images were processed using a custom macro in FIJI/ImageJ (Schindelin et al. 2012). Median immunofluorescent intensity data for the antibody of interest was measured on masks limited to the area defined by other channels (NeuN for neurons, IBA1 for microglia, and GFAP for astrocytes). This enabled protein expression measurements to be restricted to neurons, microglia, or astrocytes as relevant. Individual channel z-stacks were flattened using a maximum intensity projection, underwent background subtraction, and the mask channel was auto-thresholded using the ‘default’ method. For microglial pp38 MAPK, the mask was then limited to cell nuclei by performing an AND operation with a DAPI mask. Images were manually quality controlled for mask and quantification quality, and excluded from analysis where appropriate (details on final *n* for each comparison are available in Supplementary Tables B1- B8).

Microglial morphology in the hippocampus and mPFC was assessed by fractal analysis in line with previously published methods (Morrison et al. 2017; Fernandez-Arjona et al. 2019). Binarised masks from IBA1 + staining were segmented into single microglia using the particle analyser. All cells present in the field of view (minimum 20 cells per region of interest per rat) underwent fractal analysis in FracLac (Karperien 1999; Karperien et al. 2013) using the box counting method, with 12 grid positions and power series scaling with base 2 and exponent 2. Fractal dimension, cell area, and circularity were measured on each cell, and the final result for each region of interest was taken as the mean of all analysed cells in the field of view. The VPL thalamus was excluded from this analysis due to mean cell numbers in the field of view being insufficient to obtain robust measurements. The complete morphology data set is available in Supplementary Tables C1- C7.

### Data Analysis

Daily radial arm maze data was normalised to the total number of arm entries performed in the trial. Outliers greater than three times the interquartile range above the group mean for any measure were removed. Results were binned into three seven-day periods (days 1–7, 8–14, 15–21 post-surgery) by averaging the measure across the indicated seven days for each rat. One sham minocycline rat did not commence pellet-seeking behaviours pre-surgery and was excluded from radial maze results.

Behavioural and neuroanatomical comparisons were conducted using mixed or two-way analysis of variance (ANOVA) where appropriate. The Shapiro-Wilk test was used to determine whether data followed a normal distribution (Supplementary File D). Significant interaction or main effects were investigated with *post hoc* pairwise t tests with Holm multiple comparisons correction; the resulting adjusted p-values were used to assess statistical significance. Additional within-subjects comparisons were conducted with the Friedman test with the Wilcoxon post-hoc test and Holm multiple comparison correction (Supplementary File E). The covariance between radial maze measures was evaluated with Pearson’s correlation coefficient with Holm multiple comparisons correction. Linear regressions were conducted using general linear models. All individual data points on scatter plots denote biological, rather than technical, replicates. All results were generated in *R* (version 4.1.2 in RStudio 2022.07.2) to a significance level of α = 0.05.

## Results

### Radial Arm Maze

#### Minocycline Prevented the Development of Affective Disturbances Following CCI

**Fig. 3:**
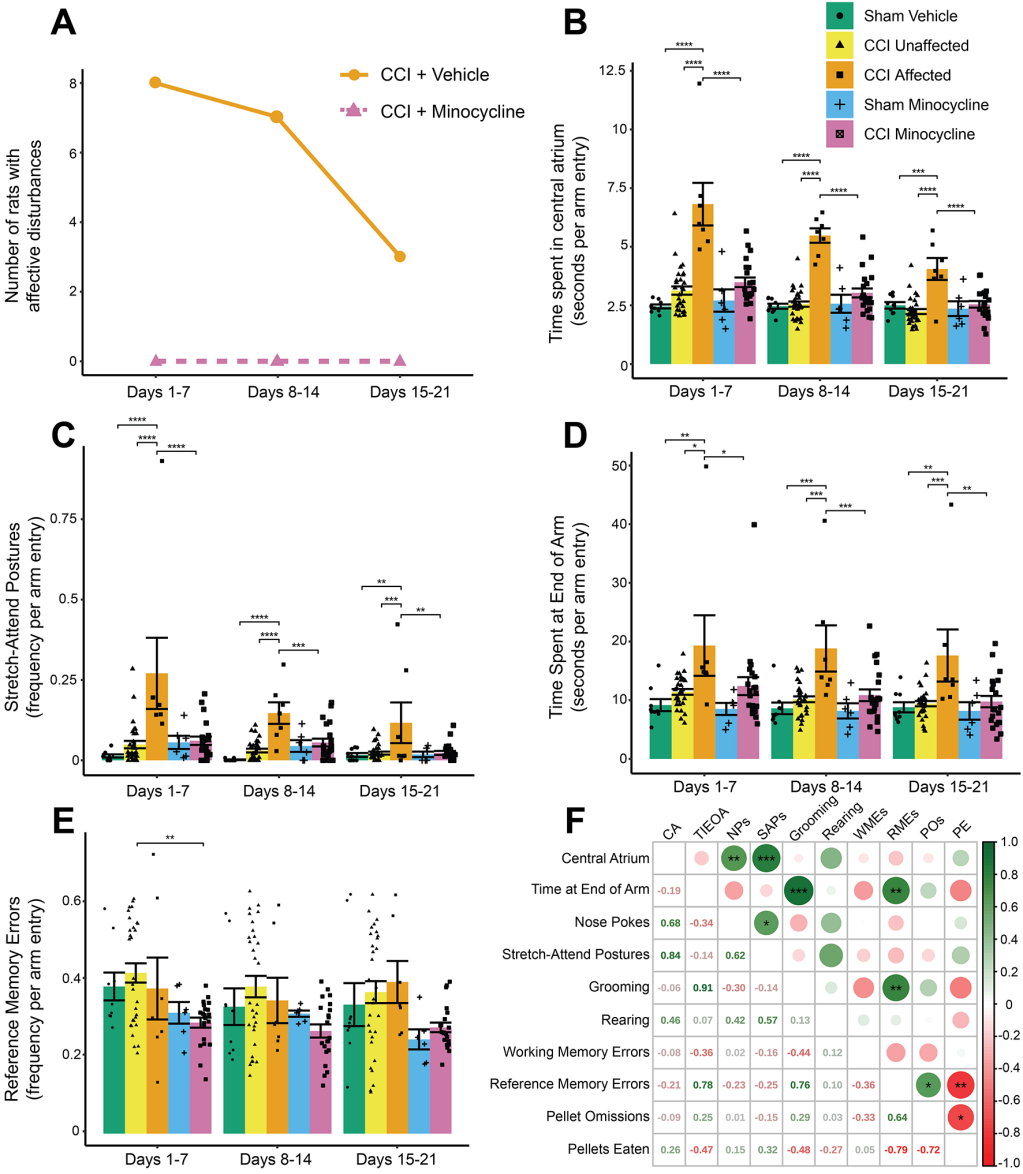
Affective and memory-related behavioural phenotyping of radial maze foraging behaviour in nerve-injured and minocycline-treated rats. (**A**) The number of rats classified as ‘*affected*’ in vehicle- and minocycline-administered groups. No minocycline-treated rats achieved the *affected* criteria. (**B**) The time spent in the central atrium of the radial maze, normalised per number of arm entries. This measure was used to define the *unaffected* and *affected* groups. (**C**) The frequency of stretch-attend postures was significantly elevated in *affected* rats at all time points compared with *unaffected* rats, CCI minocycline rats, and sham vehicle rats. (**D**) Time spent at the end of an arm was similarly elevated in *affected* rats at all time points. (**E**) The frequency of reference memory errors was lower in CCI minocycline rats than *unaffected* rats in days 1–7 post-surgery. (**F**) Correlation matrix of the Pearson correlation, and its statistical significance, between each radial maze parameter for *affected* rats. Heatmap-coloured circles above the diagonal represent the value of Pearson’s *r* where green is more positively correlated and red is more negatively correlated, with the size of the circle representing the *r* value. Values below the diagonal are the *r* values used to generate the heatmap. Statistically significant correlations are indicated with asterisks. Column graphs show group means ± standard error, with individual data points corresponding to the mean value for one rat over its daily trials in the indicated seven days. CA: time in central atrium; TIEOA: time at end of arm; NPs: nose pokes; SAPs: stretch-attend postures; WMEs: working memory errors; RMEs: reference memory errors; POs: pellet omissions; PE: pellets eaten. *P < 0.05; **P < 0.01 ***P < 0.001; ****P < 0.0001

Of the 37 vehicle-administered CCI rats, eight fulfilled criteria for affective disturbances by the end of the first week post-surgery (~ 22% of total). This number decreased to seven by the end of the second week and decreased again to three by the end of the third week. The rats fulfilling criteria by the end of the second week were taken as the *affected* group (*n* = 7), while those that never fulfilled the criteria (*n* = 21) were taken as the *unaffected* group for all subsequent analyses. If minocycline had no effect, we would have proportionally expected five minocycline-administered, nerve-injured rats to be considered *affected;* instead, in the minocycline-treated CCI group (*n* = 21), no rats fulfilled the criteria for affective disturbances at any time point. Minocycline administration was thus sufficient to prevent the development of affective disturbances following CCI (Fig. 3a).

Time spent in the central atrium of the maze was significantly different between groups at days 1–7 (group: *F*_*4,68*_ = 18.6, *P*_*adj*_ < 0.0001), days 8–14 (*F*_*4,68*_ = 25.1, *P*_*adj*_ < 0.0001) and days 15–21 (*F*_*4,68*_ = 10.2, *P*_*adj*_ < 0.0001) post-surgery (Fig. 3b). At all time points, the *affected* group spent more time in the central atrium than both CCI minocycline and sham vehicle rats (all *P*_*adj*_ < 0.001). This is expected given the definition of *affected* relied on this figure, though the value for *affected* rats decreased over the course of the experiment. The difference between sham minocycline and CCI minocycline rats did not reach significance at any time point (all *P*_*adj*_ > 0.05). In addition, the number of stretch-attend postures was significantly different between groups at all time points (days 1–7: *F*_*4,68*_ = 7.7, *P*_*adj*_ < 0.0001; days 8–14: *F*_*4,68*_ = 11.2, *P*_*adj*_ < 0.0001; days 15–21: *F*_*4,68*_ = 4.7, *P*_*adj*_ = 0.002), with higher frequency in *affected* rats than *unaffected* rats, CCI minocycline rats, and sham vehicle rats in all three post-surgery weeks (all *P*_*adj*_ < 0.01; Fig. 3c). Likewise, *affected* rats spent more time at the end of an arm (days 1–7: *F*_*4,68*_ = 3.7, *P*_*adj*_ = 0.008; days 8–14: *F*_*4,68*_ = 6.6, *P*_*adj*_ = 0.0004; days 15–21: *F*_*4,68*_ = 5.0, *P*_*adj*_ = 0.002) than *unaffected* rats, sham vehicle rats, and CCI minocycline rats in all three post-surgery time bins (all *P*_*adj*_ < 0.05). CCI minocycline rats were not different from sham monocycline rats at any time point (all *P*_*adj*_ > 0.05; Fig. 3d). There were no significant effects of experimental group in the frequency of nose pokes, time spent grooming, rearing, frequency of pellet omissions, or pellets eaten (all *P*_*adj*_ > 0.05; data not shown).

#### Minocycline Improved Reference Memory Performance in the First Week after CCI

Frequency of reference memory errors was significantly altered during the first seven days (group: *F*_*4,68*_ = 3.8, *P*_*adj*_ = 0.02), but not during days 8–14 (*F*_*4,68*_ = 2.6, *P*_*adj*_ = 0.09) or 15–21 (*F*_*4,68*_ = 2.6, *P*_*adj*_ = 0.09). CCI minocycline rats performed significantly fewer reference memory errors than did *unaffected* rats (*P*_*adj*_ = 0.003; Fig. 3e). Minocycline therefore improved cognitive function in nerve-injured animals, and the reduction in memory performance after nerve injury was independent of affective responding. There were no differences in working memory errors (all *P*_*adj*_ > 0.05).

#### Correlations between Radial Maze Measures

The covariance between each radial arm maze parameter was investigated using a correlation matrix. Independent of experimental group, there was a significant positive correlation between time in the central atrium and the number of stretch-attend postures (*r* = 0.75; *P* = 0.02) and the time spent at the end of an arm and time spent grooming (*r* = 0.80; *P* < 0.01; data not shown). This suggests that typically rats performed SAPs within the central atrium, while grooming typically took place at the end of an arm. By extension, *affected* rats, which spent significantly more time at the end of an arm, spend much of their time there grooming rather than investigating their environment.

When restricted to *affected* rats, there were additional positive relationships between time in central atrium and frequency of nose pokes (*r* = 0.68, *P* = 0.01), and between stretch-attend postures and nose pokes (*r* = 0.62, *P* = 0.03), which further suggests the increase of risk assessment behaviours taking place within the central atrium. There were also positive associations between reference memory errors and time spent at the end of an arm (*r* = 0.78, *P* < 0.01) and reference memory errors and grooming (*r* = 0.76, *P* < 0.01), indicating increased grooming at the end of an arm in arms where no sucrose pellet was found. The positive association between reference memory errors and pellet omissions (*r* = 0.64, *P* = 0.04) and negative association between reference memory errors and the number of pellets eaten (*r* = -0.79, *P* < 0.01; Fig. 3f) is an expected by the definition of a reference memory error, with no pellet available to consume.

Overall, these data suggest that minocycline was effective in preventing the emergence of a subgroup of rats with disrupted radial maze foraging behaviours after CCI.

### Minocycline Partially Attenuated Mechanical Allodynia in all Rats after CCI

**Fig. 4:**
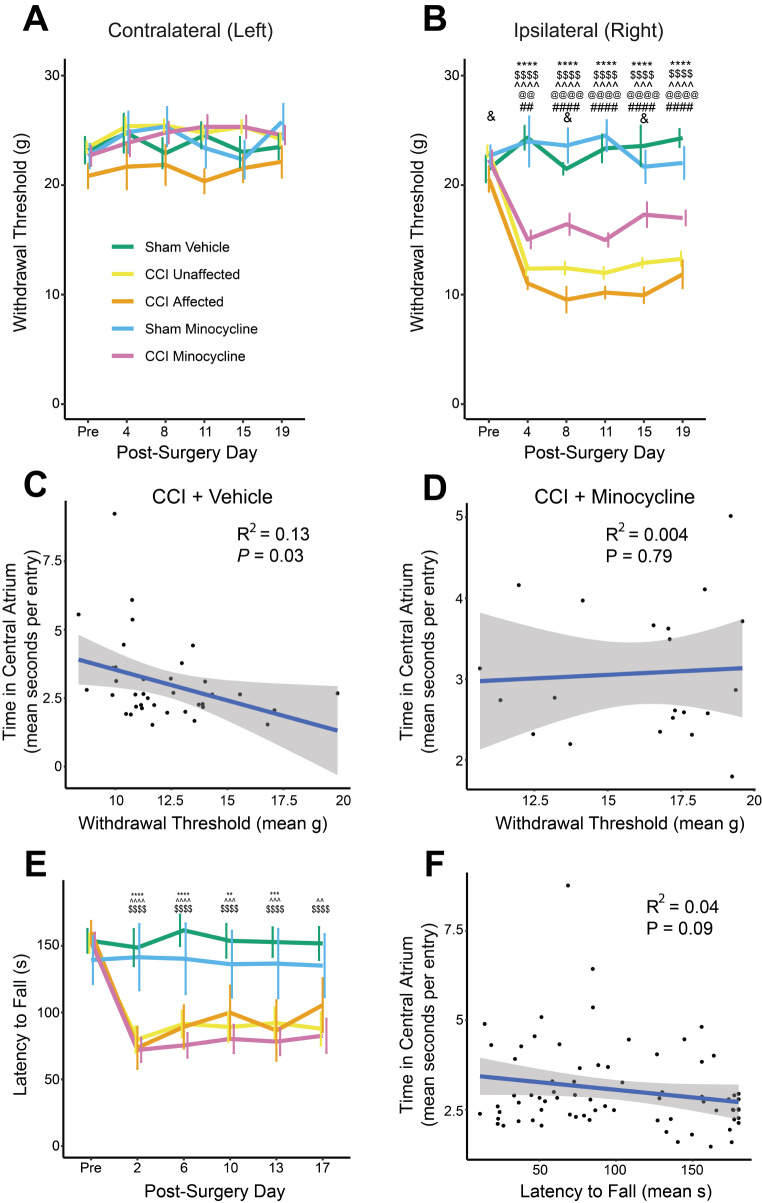
Evoked sensory and motor behaviours of nerve-injured and minocycline-treated rats. (**A**) Hindpaw withdrawal thresholds contralateral and (**B**) ipsilateral to the site of injury. Line represents the mean of all rats in the indicated experimental group ± standard error. The ‘pre’ time point represents the mean across three pre-testing days. (**C**) Linear regression between time spent in the central atrium of the radial maze and mechanical withdrawal thresholds in CCI vehicle rats (**D**) and in CCI minocycline rats. The line-of-best-fit is indicated in blue with the 95% confidence interval shaded in grey. (**E**) Latency to fall (in seconds) off the rotarod device. (**F**) Relationship between time in the central atrium and latency to fall off the rotarod device across all rats. Line graphs show group means ± standard error. Sham vehicle: *n* = 9; *unaffected*: *n* = 30; *affected*: *n* = 7; sham minocycline: *n* = 7; CCI minocycline: *n* = 21. *: Sham vehicle vs. *affected*; $: Sham vehicle vs. *unaffected*; ^: Sham minocycline vs. CCI minocycline; @: *Unaffected* vs. CCI minocycline; #: *Affected* vs. CCI minocycline; &: *Unaffected* vs. *affected*. *P_*adj*_ < 0.05; **P_*adj*_ < 0.01; ***P_*adj*_ < 0.001; ****P_*adj*_ < 0.0001

Withdrawal thresholds of the injured, ipsilateral hindpaw were significantly different (group × test day: *F*_*20,345*_ = 10.8, *P*_*adj*_ < 0.0001), but not the contralateral hindpaw (*F*_*17.51,302*_ = 1.3, *P*_*adj*_ = 0.4; Fig. 4a). There were significant decreases at all post-surgery time points in *unaffected* and *affected* rats, and CCI minocycline rats, compared to their respective sham controls (all *P*_*adj*_ < 0.001). However, CCI minocycline rats had significantly higher thresholds than both *unaffected* and *affected* rats at each post-surgery time point (all *P*_*adj*_ < 0.01). CCI therefore induced stable and long-lasting evoked mechanical sensitivity in the ipsilateral hindpaw of rats, which was partially attenuated by oral minocycline treatment. While withdrawal thresholds were significantly lower in the *affected* group compared to the *unaffected* group on days 8 and 15 post-surgery (both *P*_*adj*_ < 0.05), this difference was also present pre-surgery (*P*_*adj*_ = 0.02). This suggests that the impact of CCI on mechanical hypersensitivity was not different between affective subgroups (Fig. 4b) and potentially indicates a subtle difference in baseline sensitivity, perhaps due to hypervigilance pre-injury.

To determine whether a similar baseline difference exists in the CCI minocycline group, we performed a linear regression between pre-surgery ipsilateral withdrawal thresholds and mean time spent in the central atrium during days 1–7 post-surgery. There was no significant relationship (*R*^*2*^ = 0.05, *P* = 0.18), suggesting that pre-existing differences in mechanical allodynia did not predict later affective responses to minocycline administration after nerve injury. Additionally, specifically in CCI vehicle rats, there was a statistically significant, though small, negative association between time spent in the central atrium and evoked mechanical hypersensitivity following nerve injury (*R*^*2*^ = 0.13, *P* = 0.03; Fig. 4c). This relationship was not present in CCI minocycline rats (*R*^*2*^ = 0.004, *P* = 0.8; Fig. 4d). This observation suggests only a weak relationship between mechanical hypersensitivity and disrupted foraging behaviours after CCI, an effect abolished by minocycline.

### Minocycline did not Resolve CCI-Induced Loss of Motor Coordination

There was a significant decrease in latency to fall off the rotarod (group × test day: *F*_*10.87,187.48*_ = 4.3, *P*_*adj*_ < 0.0001) in *unaffected* compared to sham vehicle rats (all *P*_*adj*_ < 0.0001), and in sham minocycline compared to CCI minocycline rats (all *P*_*adj*_ < 0.01) at each post-surgery time point, while *affected* rats had significantly reduced latency to fall on all post-surgery days except for day 17 (all other *P*_*adj*_ < 0.01). There were no differences between CCI vehicle and CCI minocycline rats at any time point (all *P*_*adj*_ > 0.05). Minocycline could therefore not attenuate CCI-mediated disruptions in motor behaviour (Fig. 4e). There was no relationship between latency to fall off the rotarod and time spent in the central atrium (*R*^*2*^ = 0.04, *P* = 0.09; Fig. 4f).

### Supraspinal Activation Markers

To investigate the biological underpinnings of the emergence of a nerve injury-induced *affected* subgroup that was abolished by minocycline administration, we investigated supraspinal protein expression for key neuronal and glial activation and inflammatory markers.

#### Minocycline Reduced Hippocampal Neuronal Activation in Rats with Affective Disturbances

**Fig. 5:**
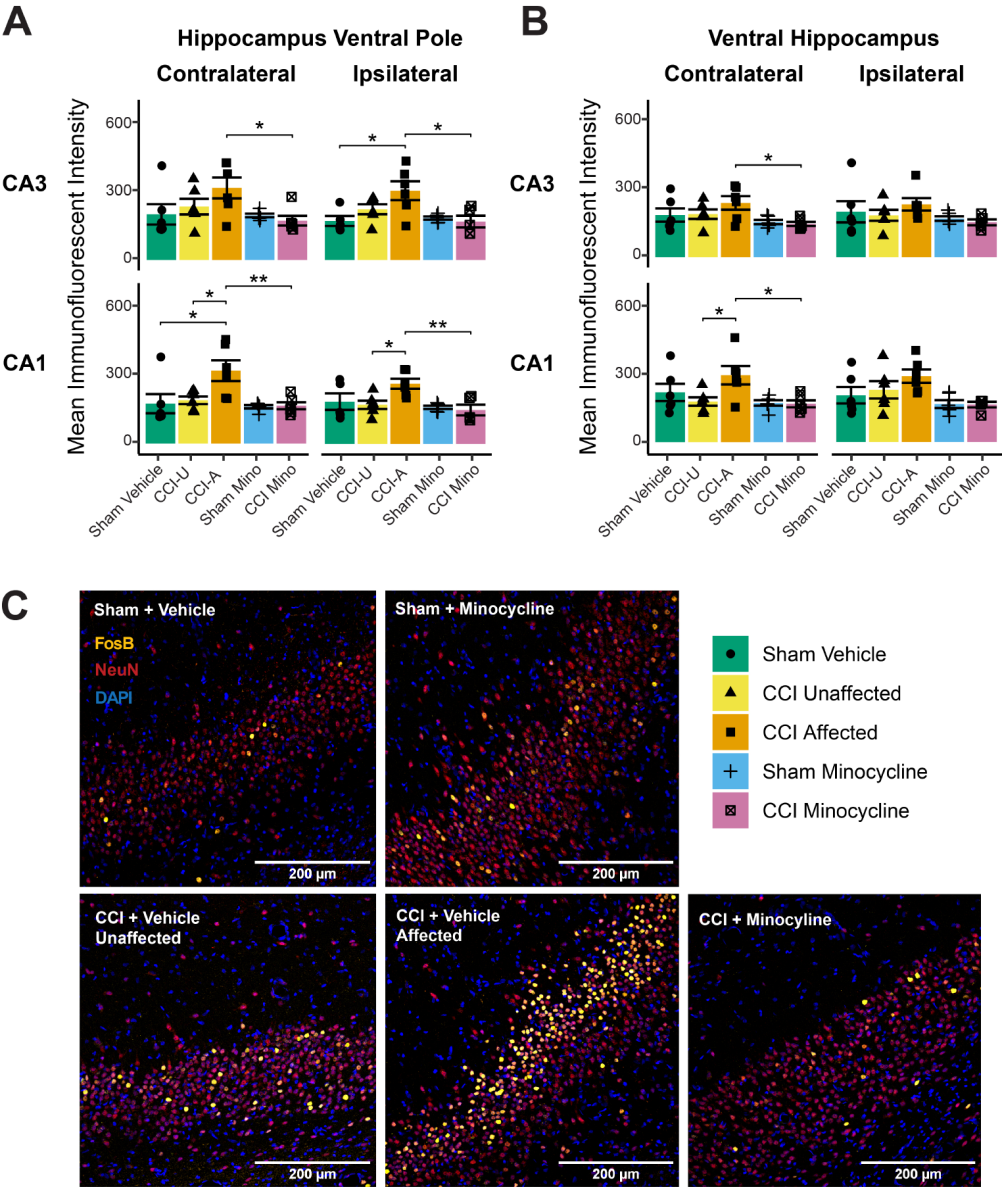
FosB immunoreactivity in the ventral hippocampus of nerve-injured and minocycline-administered rats, as measured by immunofluorescent intensity within the NeuN + portion of the image. (**A**) *Affected* rats (CCI-A) displayed significantly higher levels of FosB immunoreactivity than CCI minocycline rats in the bilateral hippocampal ventral pole CA3 and CA1 regions. In some regions, this was also significantly elevated compared to *unaffected* (CCI-U) rats. (**B**) This heightened expression was also significant in the contralateral CA3 and CA1 of the ventral hippocampus. (**C**) Representative photomicrographs of FosB/ΔFosB (yellow), NeuN (red), and DAPI (blue) staining of the contralateral ventral pole CA1 of a rat from each experimental group, showing increased FosB expression specifically in the *affected* rat. Column graphs show group means ± standard error, with individual data points showing the expression value for one rat. Immunofluorescent intensity values are expressed in arbitrary units. *P_*adj*_ < 0.05; **P_*adj*_ < 0.01

FosB immunoreactivity within NeuN + neurons was significantly altered by both CCI and minocycline (group × region of interest: *F*_*156,819*_ = 2.0, *P*_*adj*_ < 0.0001). There were significant FosB immunoreactivity increases in the hippocampal ventral pole of *affected* rats compared to sham vehicle rats in the ipsilateral CA3 (*P*_*adj*_ = 0.01) and contralateral CA1 (*P*_*adj*_ = 0.01) and compared to *unaffected* rats in the ipsilateral (*P*_*adj*_ = 0.03) and contralateral (*P*_*adj*_ = 0.02) CA1. CCI minocycline rats had significantly decreased FosB expression compared to *affected* rats in the ipsilateral (*P*_*adj*_ = 0.01) and contralateral (*P*_*adj*_ = 0.04) CA3, and the ipsilateral (*P*_*adj*_ = 0.01) and contralateral (*P*_*adj*_ = 0.008) CA1 of the hippocampal ventral pole (Fig. 5a). In the ventral hippocampus, FosB expression was elevated in *affected* rats compared to *unaffected* rats in the contralateral CA1 (*P*_*adj*_ = 0.04), and lower in CCI minocycline rats than *affected* rats in the contralateral CA3 (*P*_*adj*_ = 0.04) and CA1 (*P*_*adj*_ = 0.03; Fig. 5b). Additional FosB decreases in CCI minocycline rats compared to *affected* rats were detected in the contralateral dorsal CA3 (*P*_*adj*_ = 0.03) and CA1 (*P*_*adj*_ = 0.048) and the ipsilateral intermediate dentate gyrus (*P*_*adj*_ = 0.02; data not shown). No other hippocampal subregions showed significant differences (Fig. 5c). Neuronal FosB immunoreactivity was not significantly different between groups in any subregion of the mPFC and was not sufficiently detectable in the VPL thalamus (Supplementary Table B1). These findings indicate that minocycline administration inhibited the increase in FosB/ΔFosB expression observed in ventral hippocampal neurons of the *affected* group three weeks after CCI.

#### Lasting Morphological Alterations of Medial Prefrontal Cortex Microglia in *Affected* Rats

**Fig. 6:**
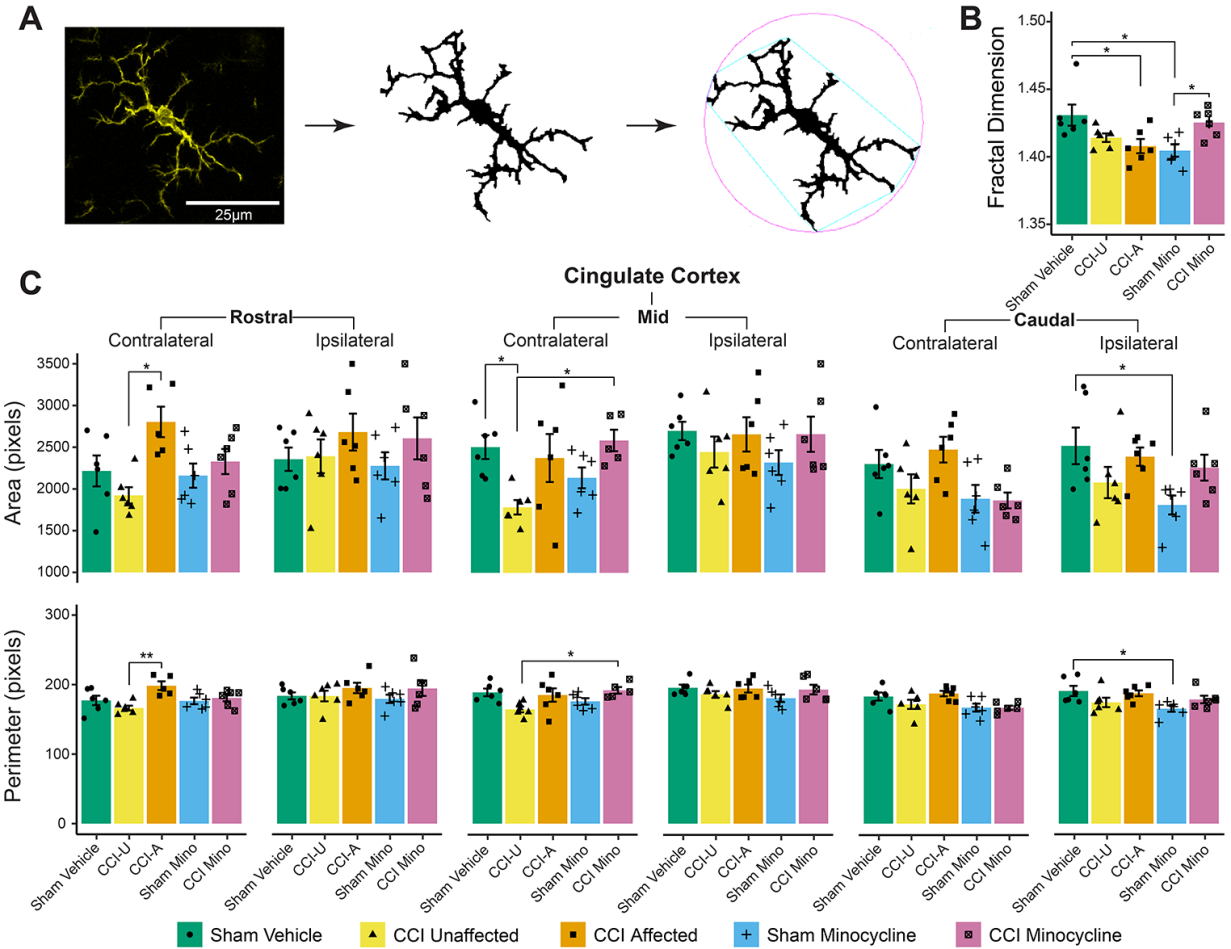
Microglia were less ramified in *affected* rats in distinct medial prefrontal cortex subregions, and minocycline alone caused morphological changes in some subregions. (**A**) Microglia were thresholded from IBA1 photomicrographs, converted to a binary mask, and underwent fractal analysis. (**B**) The mean fractal dimension in the contralateral mid infralimbic cortex. (**C**) Mean cell area and perimeter in the cingulate cortex across ipsilateral and contralateral sides and split by rostral, mid, and caudal extents. Column graphs show group means ± standard error, with individual data points showing the mean value of all technical replicates for one rat. CCI-U: CCI *unaffected*; CCI-A: CCI *affected*. *P_*adj*_ < 0.05; **P_*adj*_ < 0.01

Microglial morphology was analysed by fractal analysis (Fig. 6a). There was a significant overall effect of experimental group on microglial cell area (*F*_*4,978*_*=* 9.0, *P*_*adj*_ < 0.0001), circularity (*F*_*4,978*_*=* 8.3, *P*_*adj*_ < 0.0001), fractal dimension (a measure of geometric complexity: *F*_*4,978*_*=* 10.6, *P*_*adj*_ < 0.0001), radius (*F*_*4,978*_*=* 12.8, *P*_*adj*_ < 0.0001), and perimeter (*F*_*4,978*_*=* 13.4, *P*_*adj*_ < 0.0001), but not density (*F*_*4,978*_*=* 1.6, *P*_*adj*_ = 0.33) or lacunarity (*F*_*4,978*_*=* 1.9, *P*_*adj*_ = 0.22). When investigated in specific subregions, fractal dimension was reduced in the contralateral mid infralimbic cortex of *affected* (*P*_*adj*_ = 0.029) and sham minocycline (*P*_*adj*_ = 0.011) rats compared to sham vehicle rats, and in sham minocycline rats compared to CCI minocycline rats (*P*_*adj*_ = 0.049; Fig. 6b). Fractal dimension was also lower in the sham minocycline group compared to sham vehicle in the contralateral rostral prelimbic cortex (*P*_*adj*_ = 0.024). Fractal dimension was not significantly different in any other subregions (*P*_*adj*_ > 0.05).

Additional microglial complexity measures confirm these changes in other subregions, with cell circularity increased in the ipsilateral ventral CA1 of *affected* rats compared to sham vehicle rats (*P*_*adj*_ = 0.009). Cell area was higher in *affected* rats compared to *unaffected* rats with the contralateral rostral cingulate cortex (*P*_*adj*_ = 0.005) and contralateral intermediate CA1 (*P*_*adj*_ = 0.029); decreased in *unaffected* rats compared to sham vehicle rats within the contralateral caudal prelimbic cortex (*P*_*adj*_ = 0.035), contralateral mid cingulate cortex (*P*_*adj*_ = 0.036), and contralateral intermediate CA1 (*P*_*adj*_ = 0.047); increased in CCI minocycline rats compared to *unaffected* rats in the contralateral mid cingulate cortex (*P*_*adj*_ = 0.027); and decreased in sham minocycline rats compared to sham vehicle rats in the ipsilateral caudal cingulate (*P*_*adj*_ = 0.035) and prelimbic (*P*_*adj*_ = 0.039) cortices as well as the contralateral intermediate CA1 (*P*_*adj*_ = 0.047). Cell perimeter was also significantly higher in *affected* compared to *unaffected* rats in the contralateral rostral cingulate cortex (*P*_*adj*_ = 0.002), in CCI minocycline compared to *unaffected* rats in the contralateral mid cingulate cortex (*P*_*adj*_ = 0.037), and in sham vehicle compared to sham minocycline rats in the ipsilateral caudal cingulate (*P*_*adj*_ = 0.032) and prelimbic (*P*_*adj*_ = 0.009) cortices as well as the contralateral caudal infralimbic cortex (*P*_*adj*_ = 0.022). Results relating to the cingulate cortex are summarised in Fig. 6c. No other subregions showed significant differences for these morphological measures (*P*_*adj*_ > 0.05). The complete morphology dataset, including all non-significant comparisons, is presented in Supplementary Tables C1- C7. These results suggest lasting pro-inflammatory microglial polarisation is detectable in some neuroanatomical regions in *affected* rats at three weeks post injury, but not in CCI minocycline rats. Additionally, minocycline deramified supraspinal microglia in the absence of nerve injury.

#### Minocycline Altered Thalamic Expression of Microglial CD206 after CCI

**Fig. 7:**
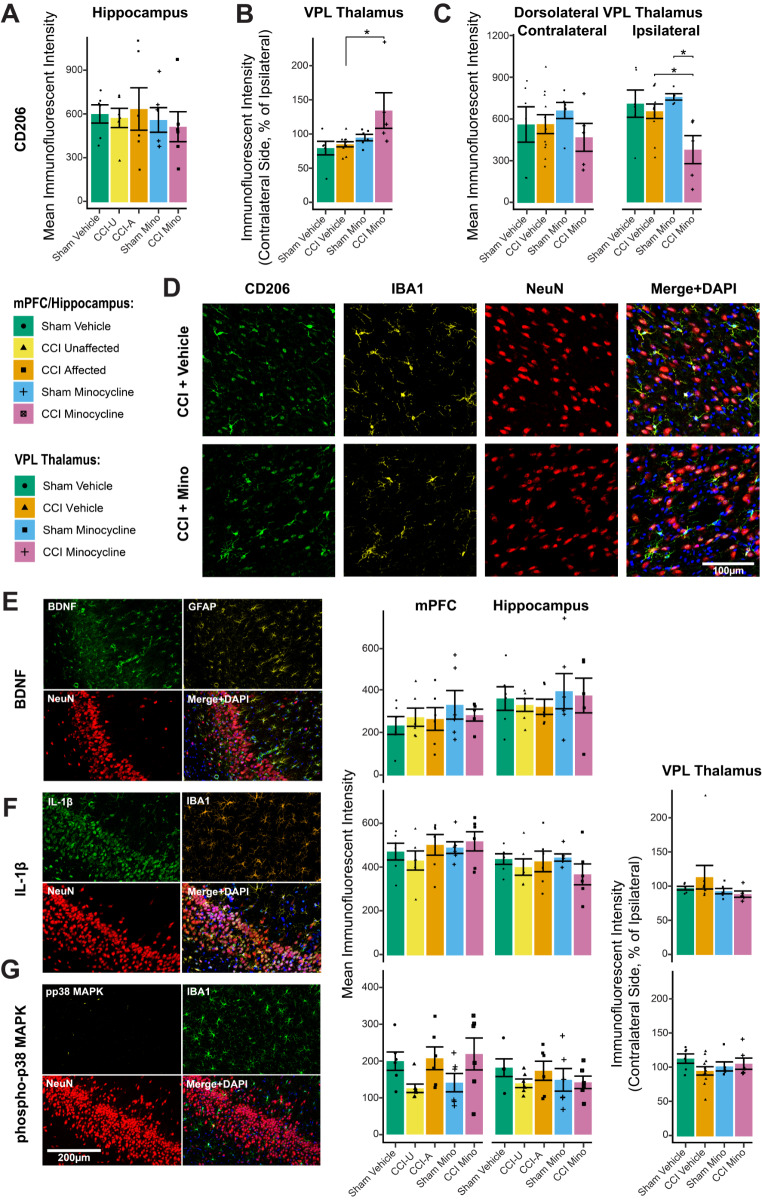
Mean immunofluorescent intensity results for glial markers. (**A**) CD206 expression on IBA1 + microglia in combined bilateral hippocampal subfields and (**B**) in the ventroposterior lateral (VPL) thalamus in the contralateral (left) side as a percentage of the ipsilateral side. (**C**) CD206 expression on IBA1 + microglia in the ipsilateral and contralateral dorsolateral VPL thalamus. (**D**) Representative photomicrographs of the ventromedial VPL thalamus contralateral to the surgery site. (**E**) Representative photomicrographs (contralateral ventral pole CA1 of an *affected* rat) and fluorescent intensity values for BDNF staining on GFAP-positive cells, (**F**) IL-1β on IBA1 + cells, and (**G**) phospho-p38 MAPK on IBA1 + cells. Column graphs show group means ± standard error, with individual data points showing the expression value for one rat. Immunofluorescent intensity values are expressed in arbitrary units for mPFC and hippocampus, and as a percentage of the ipsilateral side for the aggregated VPL thalamus. CCI: chronic constriction injury; LD: *unaffected*; HD: *affected*; mPFC: medial prefrontal cortex; CD206: cluster of differentiation 206 (mannose receptor); BDNF: brain-derived neurotrophic factor; IL-1β: interleukin-1 beta; p38 MAPK: p38 mitogen-activated protein kinase. *P_*adj*_ < 0.05; **P_*adj*_ < 0.01

At three weeks post-surgery, there were no significant differences in CD206 expression in combined bilateral hippocampal subfields when measured on IBA1 + microglia (group: *F*_*4,25*_ = 0.2, *P*_*adj*_ > 0.999) or NeuN + neurons (*F*_*4,25*_ = 0.2, *P*_*adj*_ > 0.999; Fig. 7a). However, in the VPL thalamus overall, there was a significant difference in the ratio of CD206 expression on IBA1 + cells on the contralateral side compared to the ipsilateral side (*F*_*3,24*_ = 4.4, *P*_*adj*_ = 0.03; Fig. 7b). Raw immunofluorescent intensity values were significantly lower for CCI minocycline rats in the dorsolateral extent of the ipsilateral VPL thalamus compared to CCI vehicle (*P*_*adj*_ = 0.03) and sham minocycline (*P*_*adj*_ = 0.02) rats, but not in the ventromedial extent or on the contralateral side (*P*_*adj*_ > 0.05; Fig. 7c). Representative photomicrographs are presented in Fig. 7d.

#### Other Microglia and Astrocyte Markers were not Differently Expressed Three Weeks after CCI

Three weeks after CCI, there was no effect of experimental group on BDNF expression in GFAP + astrocytes (hippocampus: *F*_*4,24*_ = 0.3, *P*_*adj*_ > 0.999; mPFC: *F*_*4,24*_ = 0.5, *P*_*adj*_ > 0.999), nor in NeuN + neurons (all *P*_*adj*_ > 0.05; Fig. 7e). BDNF expression was not observed in any measurable quantity in microglia, which is in line with previously reported mRNA expression patterns (De Felice et al. 2022). BDNF expression in IBA1 + microglia was therefore not measured. There were no significant differences in IL-1β expression on IBA1 + microglia or NeuN + neurons in the hippocampus (IBA1: *F*_*4,25*_ = 0.7, *P*_*adj*_ > 0.999; NeuN: *F*_*4,25*_ = 0.7, *P*_*adj*_ > 0.999), medial prefrontal cortex (IBA1: *F*_*4,25*_ = 0.7, *P*_*adj*_ > 0.999; NeuN: *F*_*4,25*_ = 1.9, *P*_*adj*_ = 0.426), or VPL thalamus (IBA1: *F*_*3,21*_ = 1.0, *P*_*adj*_ = 0.87; NeuN: *F*_*3,21*_ = 1.9, *P*_*adj*_ = 0.32; Fig. 7f), and no differences in phospho-p38 MAPK expression on IBA1 + DAPI + cell nuclei in the hippocampus (*F*_*4,24*_ = 0.7, *P*_*adj*_ > 0.999), medial prefrontal cortex (*F*_*4,26*_ = 2.3, *P*_*adj*_ = 0.26), or VPL thalamus (*F*_*3,25*_ = 1.3, *P*_*adj*_ = 0.60; Fig. 7g). There were no differences in GFAP or IBA1 expression between groups (all *P*_*adj*_ > 0.05).

## Discussion

Chronic oral minocycline administration completely prevented the emergence of the subgroup of rats that develop a series of disrupted foraging behaviours lasting at least two weeks after nerve injury. This is despite minocycline only achieving a partial attenuation of mechanical allodynia and having no effect on motor performance on the rotarod. Minocycline also prevented the upregulation of neuronal FosB/ΔFosB expression in the contralateral ventral hippocampus and bilateral hippocampal ventral pole seen in *affected* rats. *Affected* rats continued to express microglial morphologies consistent with low-level activation in anatomically specific subregions by three weeks post-injury, unlike CCI minocycline rats. However, we did not detect prolonged increases in microglial IL-1β and pp38 MAPK or decreased astrocytic and neuronal BDNF expression in *affected* rats at three weeks post-injury, as were previously reported at earlier time points (Fiore and Austin 2018, 2019); we therefore could not determine whether minocycline influenced these markers. Minocycline administration also resulted in microglial CD206 expression changes in the VPL thalamus, suggesting a potential supraspinal locus at which minocycline may mediate the attenuation of mechanical allodynia.

Chronic minocycline administration attenuates allodynia following sciatic nerve inflammation via key pro-inflammatory cytokines such as IL-1β and TNF-α in mouse (Ledeboer et al. 2005; Zychowska et al. 2013) and rat (Rojewska et al. 2014; Abbaszadeh et al. 2018) spinal cords. Minocycline prevents depressive-like behaviours by reducing cortical IL-1β and increasing anti-inflammatory mannose receptor (CD206/MRC2) in spinal nerve-ligated mice (Burke et al. 2014) and spared nerve-injured rats (Xu et al. 2017). Minocycline reduces depressive-like behaviours, as measured by the forced swim test, and allodynia in the streptozotocin model of type 1 diabetes mellitus (Amorim et al. 2017) and by the sucrose preference and forced swim tests in a bone cancer pain model by inhibiting hippocampal microglia (Dai et al. 2019). Here, we provide evidence in keeping with these studies that minocycline prevents the emergence of individual differences in the development of disrupted foraging behaviours and associated hippocampal-mPFC glial and neuronal activation following nerve injury.

### Affective Behaviours

The radial maze paradigm is multimodal in nature and measures multiple aspects of affective responding, including exploration, risk assessment, memory, and reward. Findings that only a subgroup of male rats that undergo CCI express altered affective responding despite similar mechanical withdrawal thresholds is consistent with previous reports in other paradigms. A subgroup of rats develop persistent disabilities in complex behaviours such as sleep-wake behaviours and social dominance in the 1–2 weeks following CCI, which relates to the coping style of the rat but not to levels of allodynia (Monassi et al. 2003; Keay et al. 2004; Mor et al. 2010; Austin et al. 2015). In a rat spared nerve injury model, rats separated by evoked mechanical hypersensitivity thresholds lower than (low-threshold) or similar to shams (high-threshold) found no differences in anxiety-like behaviours in the light/dark box, learned helplessness in the forced swim test, or contextual fear memory responses, despite some measures being different in nerve-injured animals overall (Guimarães et al. 2019). Furthermore, mice bred for high trait anxiety are differentially affected by minocycline and have their phenotype resolved in a large battery of tests including the social preference test, light-dark box, forced swim test, and elevated plus-maze (Schmidtner et al. 2019). The findings of the current study, and our previous studies, support this existing evidence that altered affective responding following nerve injury does not bear a strong direct relationship to mechanical allodynia (Fiore and Austin 2018, 2019). This is in line with the known weak relationship between pain and injury, and the conception of pain not as a sensation but rather a need state (Wall 1979). Minocycline further diminished the relationship between allodynia and affective responding within individual rats, which may result from effects in brain regions not directly involved in ascending nociceptive input.

With these other studies in mind, it is interesting to consider what the key differences between *affected* and *unaffected* rats might be in response to nerve injury. A candidate is the emotional coping style of the individual and their resulting behavioural flexibility; rats may be categorised as proactive or reactive in response to an external stressor (Coppens et al. 2010), and these rats have fundamental baseline changes in biological function across the nervous, immune, and endocrine systems (Koolhaas 2008). It is possible that the individual differences in affective responding detected in the radial maze after nerve injury are, at least in part, explained by such a difference in coping style. Investigating this further could move us toward an animal model of neuropathic pain that can better inform us why some people are more disabled by their pain than others, and are less responsive to frontline analgesics.

Though *affected* and *unaffected* rats had a similar impact of CCI on mechanical allodynia, *affected* rats had significantly lower bilateral withdrawal thresholds than *unaffected* rats at baseline. Pre-existing individual differences in nociception may exist that explain this baseline difference. Perhaps more likely, differences in emotional coping style, such as hypervigilance that drives reduced withdrawal thresholds in the environment in which the stimulus is evoked, may mediate the increased likelihood of expressing an *affected* phenotype. Given that CCI-induced mechanical allodynia takes several months to resolve (Kingery et al. 1994; Kim and Chung 1997), it would be interesting to investigate whether the time course of allodynia resolution is different between *unaffected* and *affected* rats in the weeks following nerve injury.

We attribute the absence of some anticipated glial differences between *unaffected* and *affected* CCI rats in this study to the time point at which neuroanatomical markers were evaluated at day 21 post-surgery. Previous work on individual differences in outbred Sprague-Dawley rats after CCI investigated persistent changes in complex behaviours for between 10 and 16 days post-injury (Monassi et al. 2003; Keay et al. 2004; Fiore and Austin 2018, 2019). This is the first time that investigation of radial maze foraging behaviour within the affected subgroup has been extended to the third week post-injury. Relative to the second week after nerve injury, we observed a reduction in the number of rats that met the criteria for *affected* animals. Nevertheless, *affected* rats still show significant differences in several radial maze measures at three weeks after CCI (e.g. time spent in the central atrium, stretch-attend postures, and time at the end of an arm; see Fig. 3) and subtle but persistent microglial morphological alterations in affective-related subregions such as the cingulate cortex.

The longevity of affective disturbances differs with other models. Mice continue to express depressive-like behaviours at least eight weeks after CCI, with persistent microglial changes in the hippocampus and medial prefrontal cortex (Barcelon et al. 2019). Likewise, anhedonia-like behaviours are increased, by virtue of reduced sucrose preference, for at least three weeks in a subgroup of rats following spinal nerve ligation (Xie et al. 2017). The longevity of changes in complex behaviours after nerve injury in rats is likely paradigm specific. A possible explanation is that the frequency of testing on the radial maze (daily) may contribute a retest or habituation effect where rats are less likely to express altered affective behaviours at later time points.

### Supraspinal Activation

FosB/ΔFosB is a long-half-life transcription factor which accumulates in neurons over days and weeks, and whose expression modulates hippocampal neuron excitability in the context of chronic stress, depression, and addiction (Nestler 2015; Eagle et al. 2018, 2020) and thereby influences the functional phenotype of nearby microglia (Nomaru et al. 2014). We therefore suggest that the increased neuronal FosB/ΔFosB at day 21 post-injury, after alterations in affective responding have peaked, reflects the strong pro-inflammatory environment occurring at day 14 and earlier, as previously reported (Fiore and Austin 2018, 2019). FosB/ΔFosB increases in the hippocampus and prelimbic and infralimbic cortices in a mouse model of social isolation (Noback et al. 2021), and minocycline decreases medial prefrontal FosB expression and microglial activation in the presence of chronic psychological stress (Hinwood et al. 2012). Intra-CA1 injection of minocycline reduced proinflammatory microglial polarisation in CCI rats, and mRNA profiling has revealed differences in cytokine and chemokine signalling, NFκB activation, and microglia/macrophage polarisation (He et al. 2019). These studies suggest that the presence of affective disturbances is sufficient for the upregulation of supraspinal FosB/ΔFosB expression, and the disruptions in foraging behaviour in the present study are therefore driven by factors other than, or at least additional to, modulation of ascending nociceptive signalling pathways.

The anatomically specific restriction of FosB/ΔFosB changes in affected rats to the ventral, but not dorsal, hippocampus is consistent with the well-described preferential localisation of neurons functionally connected with affective and behavioural control regions – such as the mPFC, amygdala, and nucleus accumbens – to the ventral extent of its longitudinal axis (Strange et al. 2014). We did not detect robust changes to spatial memory in this experiment, so it is therefore unsurprising to see no change in FosB/ΔFosB expression in the dorsal hippocampus, where instead spatial memory-related place neurons predominate.

Microglial morphological alterations persisted in specific subregions of the medial prefrontal cortex of *affected* rats at three weeks post-injury. We previously observed similar changes, in a greater number of subregions, at two weeks post-injury (Fiore and Austin 2018). Despite no significant differences between CCI minocycline rats and *affected* rats in these measures, the CCI minocycline group mean was consistently similar to sham vehicle rats. It is therefore possible that the promotion of a microglial pro-resolution phenotype by minocycline relates to its inhibitory effect on neuronal FosB/ΔFosB-related activation and thereby reduces affective disturbances. However, the mechanisms of action of minocycline are complex and manifold. Minocycline acts directly on microglia (Kielian et al. 2007; Kobayashi et al. 2013; Schmidtner et al. 2019), in particular by inhibiting p38 MAPK expression in vitro (Nikodemova et al. 2006) and in vivo (Cui et al. 2008). Despite this, minocycline prevents neuronal cell death via reduction of neuron-derived p38 MAPK in the absence of microglia (Lin et al. 2001; Pi et al. 2004). Minocycline also inhibits poly(ADP-ribose)-polymerase 1 (PARP-1; Alano et al. 2006), which promotes p65 NFκB-mediated transcription of TNF-α after spinal nerve ligation (Gao et al. 2020). Genetic and pharmacological PARP-1 downregulation blocks the TNF-α-mediated upregulation of matrix metalloprotease-9 (MMP-9). MMP-9 is expressed both in neurons and microglia, is transcriptionally regulated by NFκB (Kauppinen and Swanson 2005), is implicated in neuropathic pain facilitation through the cleavage of cytokines and chemokines, and is also inhibited by minocycline (Ji et al. 2009). Minocycline may therefore exert its pro-resolution effect on affective behaviours at multiple anatomical locations in both microglia and neurons simultaneously. Our observation of reduced microglial morphological complexity in minocycline-administered sham rats further suggests the effect of minocycline on microglia is highly context-dependent, attesting to the complexity of its mechanism of action and its broad-spectrum effects.

Unexpectedly, we found a decrease in microglial CD206 expression specifically localised to the ipsilateral VPL thalamus of minocycline-administered CCI rats, as opposed to an expected upregulation on the pain-affected contralateral side. Given that unilateral nerve injury can lead to favouring the contralateral side due to the emergence of protective guarding behaviours (Kawasaki-Yatsugi et al. 2012), minocycline acting at the thalamus may facilitate adaptations in the unaffected limb. This hypothesis requires further investigation.

### Study Limitations

There are some additional key considerations in interpreting these results. Most importantly, spinal pro-inflammatory mediators and glial cells, including microglia, are involved in the initiation and maintenance of neuropathic pain (Beggs and Salter 2010; Austin and Moalem-Taylor 2010; Grace et al. 2011; Fiore et al. 2022; Tansley et al. 2022a, b). The systemic administration of minocycline utilised in the current study therefore likely also engaged cells in the spinal cord and/or periphery in additional to the supraspinal modulations observed. Nevertheless, we observed that minocycline resolved affective disturbances without modulating mechanical allodynia differently between *affected* and *unaffected* rats, which provides support for the involvement of supraspinal processing. Other supraspinal areas are likely also involved.

While multiple measures of affective responding were measured in these rats, it is likely that the manual analysis of specific behaviours missed key aspects of the phenotype we describe here. Unsupervised methods of assessing spontaneous behaviours represent a future opportunity to better characterise this phenotype of affective responding (Bohic et al. 2023). The minocycline administration was chronic and immediate following CCI, and therefore attenuated neuropathic pain during establishment. Delayed administration studies suggest that minocycline must commence less than five days after nerve injury to effectively attenuate mechanical allodynia (Raghavendra et al. 2003; Ledeboer et al. 2005; Mei et al. 2011); it is unclear whether the same would be true for these affective disturbances.

Furthermore, we only performed this investigation in male rats. The known sex differences that exist in the role of spinal microglia in mechanical hypersensitivity in mice (Sorge et al. 2015; Lopes et al. 2017; Huck et al. 2021; Tu et al. 2022) and in affective responding to minocycline (Liu et al. 2023) mean that these individual differences following nerve injury must be investigated in female rats to determine whether alternate central inflammatory changes are involved. We also performed the study on individually housed rats, and we cannot rule out that reduced contact with other rats affected the development of altered affective responses to nerve injury. It is important to consider that activated microglia may not necessarily be facilitating these alterations in affective responding, and indeed may play some role in their resolution. Even so, the ‘anti-inflammatory’ spectrum of effects of minocycline may sometimes be deleterious, particularly in the initiation of pain where some pro-inflammatory contexts are important for resolution (Parisien et al. 2022). Finally, assessing the molecular effects of minocycline at earlier time points during the time of peak expression of affective disturbances will be important to more completely elucidate the relationship between minocycline, mechanical allodynia, and affective disturbances following nerve injury.

## Conclusions

Minocycline administration abrogates multiple measures of disrupted foraging behaviours and affective responding in the subgroup of rats that express such behaviours after nerve injury, and prevents related supraspinal neuronal activation. Moreover, minocycline partially attenuated mechanical allodynia, with changes in microglial morphology suggesting alterations in microglial polarisation. Future studies should investigate whether these neuromodulatory effects occur principally via direct interaction with hippocampal neurons or indirectly by repolarisation of surrounding microglia. Deepening our understanding of the supraspinal neuroinflammatory changes that occur when neuropathic pain and affective disabilities such as depression interact is critical to designing effective strategies that improve the quality of life of people living with neuropathic pain.

## Electronic Supplementary Material

Below is the link to the electronic supplementary material.

Supplementary Material 1

Supplementary Material 2

Supplementary Material 3

Supplementary Material 4

Supplementary Material 5

## Data Availability

Data is provided within the manuscript or supplementary information files.
