# Supplementary material for: Minocycline Abrogates Individual Differences in Nerve Injury-Evoked Affective Disturbances in Male Rats and Prevents Associated Supraspinal Neuroinflammation
Source: J Neuroimmune Pharmacol. 2024 Jun 15;19(1):30. doi: 10.1007/s11481-024-10132-y (PMC11180027; doi:10.1007/s11481-024-10132-y)
Supplement: Supplementary file 1 — Supplementary Material 1 [file 11481_2024_10132_MOESM1_ESM.docx]

**Supplementary File A. Immunolabelling and microscopy parameters.**

**Supplementary Table A1.** Immunofluorescence staining parameters, including specific details on each experiment’s incubation steps and antibodies used. All primary antibody incubations were performed at 4°C. All secondary antibody incubations were performed at RT for 3 hours, and streptavidin incubations at RT for 2 hours. BDNF: brain-derived neurotrophic factor; CD206: cluster of differentiation 206 (mannose receptor); GFAP: glial fibrillary acidic protein; IBA1: ionised calcium binding adaptor molecule 1; IL-1B: interleukin 1-beta; NeuN: mAb A60 (neuronal nuclei); pp38 MAPK: phosphorylated p38 mitogen-activated protein kinase.

| **Experiment** | **First Primary Antibody Incubation** | **First Secondary Antibody Incubation** | **Tertiary Streptavidin Amplification** | **Second Primary Antibody Incubation** | **Second Secondary Antibody Incubation** |
| --- | --- | --- | --- | --- | --- |
| FosB-NeuN | 1:300 rabbit anti-rat FosB/ΔFosB [Invitrogen MA5-15056, RRID:AB_10983364] and 1:500 mouse anti-rat NeuN [Millipore MAB377, RRID:AB_2298772], 3 nights (~72 hours) | 1:100 donkey anti-rabbit Fab fragment-biotin [Jackson 711-067-003, RRID:AB_2340595] and 1:200 donkey anti-mouse Fab fragment-Alexa Fluor 647 [Jackson 715-607-003, RRID:AB_2340867] | 1:500 streptavidin-Alexa Fluor 568 [Invitrogen S11226] | n/a | n/a |
| pp38 MAPK-IBA1-NeuN | 1:300 rabbit anti-rat phospho-p38 MAPK [Cell Signalling Technologies, #4511, RRID:AB_2139682], 3 nights (~72 hours) | 1:100 donkey anti-rabbit Fab fragment-biotin [Jackson 711-067-003, RRID:AB_2340595] | 1:500 streptavidin-Alexa Fluor 568 [Invitrogen S11226] | 1:1000 rabbit anti-rat IBA1 [Abcam ab178846, RRID:AB_2636859] and 1:500 mouse anti-rat NeuN [Millipore MAB377, RRID:AB_2298772], 2 nights (~48 hours) | 1:200 donkey anti-rabbit IgG-Alexa Fluor 488 [Jackson 711-545-152, RRID:AB_2313584] and 1:200 donkey anti-mouse Fab fragment-Alexa Fluor 647 [Jackson 715-607-003, RRID:AB_2340867] |
| IL-1B-IBA1-NeuN | 1:400 rabbit anti-rat IL-1B [Abcam ab9722, RRID:AB_308765], 3 nights (~72 hours) | 1:100 donkey anti-rabbit Fab fragment-Alexa Fluor 488 [Jackson 711-547-003, RRID:AB_2340620] | n/a | 1:1000 rabbit anti-rat IBA1 [Abcam ab178846, RRID:AB_2636859] and 1:500 mouse anti-rat NeuN [Millipore MAB377, RRID:AB_2298772], 2 nights (~48 hours) | 1:500 donkey anti-rabbit IgG-Alexa Fluor 568 [Invitrogen A10042, RRID:AB_2534017] and 1:200 donkey anti-mouse Fab fragment-Alexa Fluor 647 [Jackson 715-607-003, RRID:AB_2340867] |
| CD206-IBA1-NeuN | 1:750 rabbit anti-rat CD206 [Abcam ab64693, RRID:AB_1523910], 1 night (~24 hours) | 1:100 donkey anti-rabbit Fab fragment-Alexa Fluor 488 [Jackson 711-547-003, RRID:AB_2340620] | n/a | 1:1000 rabbit anti-rat IBA1 [Abcam ab178846, RRID:AB_2636859] and 1:500 mouse anti-rat NeuN [Millipore MAB377, RRID:AB_2298772], 2 nights (~48 hours) | 1:500 donkey anti-rabbit IgG-Alexa Fluor 568 [Invitrogen A10042, RRID:AB_2534017] and 1:200 donkey anti-mouse Fab fragment-Alexa Fluor 647 [Jackson 715-607-003, RRID:AB_2340867] |
| BDNF-GFAP-NeuN | 1:300 rabbit anti-rat BDNF [Abcam ab108319 RRID:AB_10862052], 2 nights (~48hrs) | 1:100 donkey anti-rabbit Fab fragment-Alexa Fluor 488 [Jackson 711-547-003, RRID:AB_2340620] | n/a | 1:2000 rabbit anti-rat GFAP [Abcam ab7260, RRID:AB_305808] and 1:500 mouse anti-rat NeuN [Millipore MAB377, RRID:AB_2298772], 2 nights (~48 hours) | 1:500 donkey anti-rabbit IgG-Alexa Fluor 568 [Invitrogen A10042, RRID:AB_2534017] and 1:200 donkey anti-mouse Fab fragment-Alexa Fluor 647 [Jackson 715-607-003, RRID:AB_2340867] |

**Supplementary Table A2.** Confocal immunofluorescence image acquisition parameters for frozen fixed coronal rat brain. Images taken at 60× magnification oil immersion objective as a 2×2 tile of adjacent fields of view, which were stitched with 5% overlap by optimal path to a final area of 408µm × 408µm ~0.17mm^2^ per region of interest. 20× magnification images were of a single field of view with an area of 512µm × 512µm (~0.26mm^2^). All images were acquired with a DAPI channel on the 405nm laser line. Two Nikon C2+ microscopes were used for different experiments (indicated by A and B). mPFC: medial prefrontal cortex; Hipp: hippocampus; Thal: ventroposterolateral nucleus of the thalamus; AF: Alexa Fluor.

| Stain | Region | Objective | Digital Zoom (×) | Image Resolution (px) | Pixel Dwell | Average Factor | Microscope | Antigen | Fluorophore | Laser Intensity | Digital Gain (HV) | Offset |
| --- | --- | --- | --- | --- | --- | --- | --- | --- | --- | --- | --- | --- |
| FosB-NeuN | Hipp, mPFC | 20× | 1.23 | 1024 | 2.4 | 1 | A | FosB | AF568 | 3.5 | 92 | -30 |
|  |  |  |  |  |  |  |  | NeuN | AF647 | 2.5 | 82 | -5 |
| IL-1β-IBA1-NeuN | Hipp | 60× | 1.0 | 1024 | 2.4 | 1 | B | IL-1β | AF488 | 2.5 | 83 | -10 |
|  |  |  |  |  |  |  |  | IBA1 | AF568 | 2.5 | 81 | 0 |
|  |  |  |  |  |  |  |  | NeuN | AF647 | 1.2 | 78 | 3 |
|  | mPFC | 20× | 1.0 | 1024 | 2.4 | 1 | B | IL-1β | AF488 | 3.0 | 85 | -55 |
|  |  |  |  |  |  |  |  | IBA1 | AF568 | 2.5 | 82 | -10 |
|  |  |  |  |  |  |  |  | NeuN | AF647 | 1.2 | 76 | 0 |
|  | Thal | 20× | 1.0 | 1024 | 2.4 | 1 | B | IL-1β | AF488 | 2.0 | 77 | -20 |
|  |  |  |  |  |  |  |  | IBA1 | AF568 | 2.5 | 80 | -15 |
|  |  |  |  |  |  |  |  | NeuN | AF647 | 2.0 | 80 | -10 |
| p38-IBA1-NeuN | Hipp | 20× | 1.23 | 1024 | 2.4 | 2 | A | p38 MAPK | AF568 | 5.0 | 90 | -30 |
|  |  |  |  |  |  |  |  | IBA1 | AF488 | 1.3 | 70 | -2 |
|  |  |  |  |  |  |  |  | NeuN | AF647 | 2.0 | 85 | 0 |
|  | mPFC | 20× | 1.23 | 1024 | 2.4 | 2 | A | p38 MAPK | AF568 | 5.0 | 90 | -40 |
|  |  |  |  |  |  |  |  | IBA1 | AF488 | 1.3 | 70 | -2 |
|  |  |  |  |  |  |  |  | NeuN | AF647 | 2.0 | 85 | 0 |
|  | Thal | 20× | 1.0 | 1024 | 2.4 | 2 | B | p38 MAPK | AF568 | 4.5 | 90 | -45 |
|  |  |  |  |  |  |  |  | IBA1 | AF488 | 1.5 | 74 | -10 |
|  |  |  |  |  |  |  |  | NeuN | AF647 | 1.8 | 77 | -2 |
| CD206-IBA1-NeuN | Hipp | 60× | 1.0 | 1024 | 2.4 | 1 | A | CD206 | AF488 | 2.0 | 80 | -40 |
|  |  |  |  |  |  |  |  | IBA1 | AF568 | 2.0 | 80 | -5 |
|  |  |  |  |  |  |  |  | NeuN | AF647 | 2.0 | 87 | 0 |
|  | Thal | 20× | 1.23 | 1024 | 2.4 | 1 | A | CD206 | AF488 | 2.2 | 70 | -25 |
|  |  |  |  |  |  |  |  | IBA1 | AF568 | 2.5 | 77 | -10 |
|  |  |  |  |  |  |  |  | NeuN | AF647 | 2.5 | 88 | -7 |
| BDNF-GFAP-NeuN | Hipp | 20× | 1.23 | 1024 | 2.4 | 1 | A | BDNF | AF488 | 1.5 | 70 | -20 |
|  |  |  |  |  |  |  |  | GFAP | AF568 | 2.2 | 80 | -12 |
|  |  |  |  |  |  |  |  | NeuN | AF647 | 1.5 | 82 | 0 |
|  | mPFC | 20× | 1.23 | 2048 | 2.6 | 1 | A | BDNF | AF488 | 1.2 | 66 | -12 |
|  |  |  |  |  |  |  |  | GFAP | AF568 | 2.2 | 80 | -20 |
|  |  |  |  |  |  |  |  | NeuN | AF647 | 1.5 | 82 | 0 |
|  | Thal | 20× | 1.23 | 2048 | 1.2 | 1 | A | BDNF | AF488 | 2.0 | 70 | -25 |
|  |  |  |  |  |  |  |  | GFAP | AF568 | 3.0 | 87 | -55 |
|  |  |  |  |  |  |  |  | NeuN | AF647 | 2.5 | 90 | -10 |
