# Supplementary material for: Minocycline Abrogates Individual Differences in Nerve Injury-Evoked Affective Disturbances in Male Rats and Prevents Associated Supraspinal Neuroinflammation
Source: J Neuroimmune Pharmacol. 2024 Jun 15;19(1):30. doi: 10.1007/s11481-024-10132-y (PMC11180027; doi:10.1007/s11481-024-10132-y)
Supplement: Supplementary file 2 — Supplementary Material 2 [file 11481_2024_10132_MOESM2_ESM.docx]

**Supplementary File B. Mean fluorescent intensity values and *n*-values (by anatomical region of interest and experimental group).**

**Supplementary Table B1**. Immunofluorescent intensity values of FosB/ΔFosB within NeuN+ areas of staining. Values represent group means ± standard deviation in arbitrary mean pixel intensity units. Results where *n* < 3 are excluded. Hipp: hippocampus; mPFC: medial prefrontal cortex; VPL: ventroposterior lateral nucleus; int: intermediate; DG: dentate gyrus; CG: cingulate gyrus; PL: prelimbic cortex; IL: infralimbic cortex; DL: dorsolateral; VM: ventromedial; ZI: zona incerta. *: Sham vehicle vs CCI affected; $: Sham vehicle vs CCI unaffected; ^: Sham minocycline vs CCI minocycline; @: CCI unaffected vs CCI minocycline; #: CCI affected vs CCI minocycline; &: CCI unaffected vs CCI affected.

|  | | | Left (Contralateral) | | | | | Right (Ipsilateral) | | | | |
| --- | --- | --- | --- | --- | --- | --- | --- | --- | --- | --- | --- | --- |
| Region | AP Level | Subregion | Sham Vehicle | CCI Unaffected | CCI Affected | Sham Minocycline | CCI Minocycline | Sham Vehicle | CCI Unaffected | CCI Affected | Sham Minocycline | CCI Minocycline |
| Hipp | Dorsal | DG | 300.3 ± 155.2  (*n*=6) | 387.1 ± 199.7  (*n*=6) | 470.5 ± 213.4  (*n*=6) | 302.6 ± 75.3  (*n*=6) | 279.3 ± 103.7  (*n*=6) | 269.1 ± 100.1  (*n*=6) | 385.6 ± 195.5  (*n*=6) | 510.7 ± 228.1  (*n*=6) | 317.1 ± 94.2  (*n*=6) | 256.9 ± 67.1  (*n*=6) |
|  |  | CA3 | 144.3 ± 53.1  (*n*=6) | 166.3 ± 60.0  (*n*=6) | 220.6 ± 80.0^#^  (*n*=6) | 125.6 ± 18.4  (*n*=6) | 127.1 ± 24.6  (*n*=6) | 158.3 ± 84.2  (*n*=6) | 156.7 ± 45.4  (*n*=6) | 207.1 ± 91.6  (*n*=6) | 123.5 ± 8.9  (*n*=6) | 130.2 ± 19.4  (*n*=6) |
|  |  | CA1 | 162.9 ± 76.8  (*n*=6) | 191.7 ± 78.7  (*n*=6) | 250.1 ± 90.5^#^  (*n*=6) | 135.3 ± 21.9  (*n*=6) | 138.4 ± 27.9  (*n*=6) | 184.1 ± 122.3  (*n*=6) | 216.2 ± 101.7  (*n*=6) | 243.3 ± 105.6  (*n*=6) | 127.5 ± 14.4  (*n*=6) | 138.4 ± 27.8  (*n*=6) |
|  | Int | DG | 280.3 ± 184.6  (*n*=6) | 324.0 ± 168.0  (*n*=6) | 438.5 ± 223.4  (*n*=6) | 264.6 ± 74.2  (*n*=6) | 211.4 ± 73.2  (*n*=6) | 244.6 ± 100.6  (*n*=5) | 328.8 ± 152.8  (*n*=6) | 447.5 ± 175.0^#^  (*n*=6) | 246.5 ± 58.6  (*n*=6) | 205.4 ± 49.6  (*n*=5) |
|  |  | CA3 | 170.4 ± 75.5  (*n*=6) | 207.5 ± 57.4  (*n*=6) | 225.4 ± 107.5  (*n*=6) | 155.3 ± 25.0  (*n*=6) | 149.2 ± 19.5  (*n*=6) | 159.6 ± 56.6  (*n*=5) | 189.6 ± 62.4  (*n*=6) | 229.0 ± 67.9  (*n*=6) | 151.0 ± 26.4  (*n*=6) | 139.7 ± 22.3  (*n*=5) |
|  |  | CA1 | 195.9 ± 94.3  (*n*=6) | 237.9 ± 129.2  (*n*=6) | 259.6 ± 120.7  (*n*=6) | 175.6 ± 34.6  (*n*=6) | 154.7 ± 26.9  (*n*=6) | 164.2 ± 51.1  (*n*=5) | 236.9 ± 113.2  (*n*=6) | 236.0 ± 90.7  (*n*=6) | 148.9 ± 21.6  (*n*=6) | 129.9 ± 25.6  (*n*=5) |
|  | Ventral | DG | 325.1 ± 303.5  (*n*=6) | 243.0 ± 109.4  (*n*=6) | 406.9 ± 114.4  (*n*=6) | 211.0 ± 33.2  (*n*=6) | 201.4 ± 48.0  (*n*=6) | 275.1 ± 191.6  (*n*=6) | 283.3 ± 152.9  (*n*=6) | 476.6 ± 185.3  (*n*=6) | 237.3 ± 90.0  (*n*=6) | 216.1 ± 97.2  (*n*=5) |
|  |  | CA3 | 185.6 ± 71.2  (*n*=6) | 189.7 ± 51.3  (*n*=6) | 239.2 ± 72.9^#^  (*n*=6) | 154.7 ± 23.4  (*n*=6) | 146.8 ± 22.4  (*n*=6) | 200.2 ± 114.3  (*n*=6) | 184.5 ± 59.3  (*n*=6) | 232.9 ± 67.1  (*n*=6) | 169.9 ± 24.6  (*n*=6) | 153.5 ± 29.0  (*n*=5) |
|  |  | CA1 | 219.0 ± 93.2  (*n*=6) | 178.8 ± 45.6  (*n*=6) | 294.8 ± 99.3^#/&^  (*n*=6) | 172.6 ± 30.0  (*n*=6) | 168.7 ± 37.8  (*n*=6) | 205.6 ± 88.7  (*n*=6) | 229.5 ± 93.0  (*n*=6) | 289.8 ± 72.0  (*n*=6) | 166.7 ± 42.6  (*n*=6) | 164.4 ± 27.6  (*n*=5) |
|  | Ventral Pole | CA3 | 201.2 ± 109.9  (*n*=6) | 235.3 ± 84.2  (*n*=6) | 317.6 ± 113.1^#^  (*n*=6) | 196.5 ± 19.4  (*n*=6) | 173.2 ± 52.4  (*n*=6) | 171.9 ± 49.2  (*n*=5) | 223.6 ± 54.0  (*n*=6) | 305.5 ± 101.7*^/#^  (*n*=6) | 184.9 ± 17.1  (*n*=6) | 169.3 ± 57.5  (*n*=5) |
|  |  | CA1 | 175.5 ± 102.9  (*n*=6) | 190.6 ± 41.7  (*n*=6) | 321.0 ± 112.1*^/##/&^  (*n*=6) | 162.1 ± 17.8  (*n*=6) | 166.5 ± 37.7  (*n*=6) | 185.9 ± 81.4  (*n*=5) | 172.1 ± 44.6  (*n*=6) | 264.7 ± 53.8^##/&^  (*n*=6) | 161.0 ± 17.7  (*n*=6) | 149.1 ± 52.5  (*n*=5) |
| mPFC | Rostral | CG | 138.5 ± 34.7  (*n*=6) | 178.2 ± 77.9  (*n*=6) | 167.6 ± 50.2  (*n*=6) | 165.7 ± 43.3  (*n*=6) | 153.3 ± 34.6  (*n*=6) | 192.2 ± 84.0  (*n*=6) | 185.5 ± 91.4  (*n*=6) | 217.0 ± 92.3  (*n*=6) | 166.4 ± 38.0  (*n*=6) | 165.8 ± 35.9  (*n*=6) |
|  |  | PL | 185.9 ± 93.6  (*n*=6) | 143.7 ± 25.0  (*n*=6) | 173.2 ± 49.9  (*n*=6) | 162.1 ± 29.3  (*n*=6) | 165.6 ± 44.9  (*n*=6) | 196.8 ± 78.1  (*n*=6) | 180.7 ± 56.2  (*n*=6) | 203.4 ± 72.6  (*n*=6) | 164.1 ± 37.9  (*n*=6) | 140.7 ± 27.1  (*n*=6) |
|  |  | IL | 179.0 ± 114.2  (*n*=6) | 145.6 ± 26.4  (*n*=6) | 189.6 ± 59.5  (*n*=6) | 158.6 ± 33.7  (*n*=6) | 162.8 ± 48.2  (*n*=6) | 197.1 ± 87.8  (*n*=6) | 154.3 ± 25.8  (*n*=6) | 203.5 ± 62.0  (*n*=6) | 148.0 ± 35.4  (*n*=6) | 139.8 ± 24.3  (*n*=6) |
|  | Mid | CG | 147.8 ± 47.2  (*n*=6) | 177.2 ± 86.3  (*n*=6) | 173.9 ± 57.0  (*n*=6) | 169.0 ± 29.5  (*n*=6) | 152.5 ± 57.6  (*n*=5) | 152.4 ± 60.8  (*n*=6) | 192.8 ± 90.1  (*n*=6) | 193.2 ± 84.3  (*n*=6) | 146.7 ± 24.2  (*n*=6) | 152.1 ± 39.3  (*n*=6) |
|  |  | PL | 177.8 ± 49.3  (*n*=6) | 157.0 ± 27.6  (*n*=6) | 183.1 ± 37.2  (*n*=6) | 163.4 ± 34.3  (*n*=6) | 155.9 ± 56.4  (*n*=5) | 155.6 ± 30.9  (*n*=6) | 169.4 ± 49.5  (*n*=6) | 169.8 ± 30.4  (*n*=6) | 153.0 ± 18.0  (*n*=6) | 144.1 ± 33.2  (*n*=6) |
|  |  | IL | 181.6 ± 34.7  (*n*=6) | 188.1 ± 38.8  (*n*=6) | 203.9 ± 38.2  (*n*=6) | 171.0 ± 33.7  (*n*=6) | 173.6 ± 76.1  (*n*=5) | 156.3 ± 33.3  (*n*=6) | 178.4 ± 43.5  (*n*=6) | 176.7 ± 43.7  (*n*=6) | 165.8 ± 28.1  (*n*=6) | 187.0 ± 69.1  (*n*=6) |
|  | Caudal | CG | 145.5 ± 38.1  (*n*=6) | 174.5 ± 72.5  (*n*=6) | 166.7 ± 54.0  (*n*=6) | 157.6 ± 35.3  (*n*=6) | 132.8 ± 33.9  (*n*=6) | 148.7 ± 19.5  (*n*=6) | 180.0 ± 74.2  (*n*=6) | 180.6 ± 90.0  (*n*=6) | 149.8 ± 36.3  (*n*=6) | 144.0 ± 37.1  (*n*=6) |
|  |  | PL | 148.9 ± 38.0  (*n*=6) | 155.6 ± 43.1  (*n*=6) | 158.3 ± 50.3  (*n*=6) | 153.5 ± 39.0  (*n*=6) | 132.0 ± 34.4  (*n*=6) | 151.6 ± 40.9  (*n*=6) | 159.6 ± 24.1  (*n*=6) | 167.8 ± 47.8  (*n*=6) | 155.7 ± 48.7  (*n*=6) | 122.3 ± 28.6  (*n*=6) |
|  |  | IL | 171.0 ± 39.9  (*n*=6) | 152.4 ± 28.7  (*n*=6) | 179.5 ± 36.6  (*n*=6) | 163.8 ± 24.2  (*n*=6) | 159.2 ± 52.8  (*n*=6) | 158.9 ± 32.4  (*n*=6) | 162.2 ± 13.6  (*n*=6) | 173.6 ± 51.3  (*n*=6) | 173.5 ± 42.3  (*n*=6) | 146.3 ± 40.7  (*n*=6) |
| VPL Thalamus | | DL | - | - | - | - | - | - | - | - | - | - |
|  |  | VM | - | - | - | - | - | - | - | - | - | - |
|  |  | ZI | - | - | - | - | - | - | - | - | - | - |

**Supplementary Table B2.** Immunofluorescent intensity values of BDNF within GFAP+ areas of staining. Values represent group means ± standard deviation in arbitrary mean pixel intensity units. Results where *n* < 3 are excluded. Hipp: hippocampus; mPFC: medial prefrontal cortex; VPL: ventroposterior lateral nucleus; int: intermediate; DG: dentate gyrus; CG: cingulate gyrus; PL: prelimbic cortex; IL: infralimbic cortex; DL: dorsolateral; VM: ventromedial; ZI: zona incerta.

|  | | | Left (Contralateral) | | | | | Right (Ipsilateral) | | | | |
| --- | --- | --- | --- | --- | --- | --- | --- | --- | --- | --- | --- | --- |
| Region | AP Level | Subregion | Sham Vehicle | CCI Unaffected | CCI Affected | Sham Minocycline | CCI Minocycline | Sham Vehicle | CCI Unaffected | CCI Affected | Sham Minocycline | CCI Minocycline |
| Hipp | Dorsal | DG | 511.4 ± 240.2  (*n*=6) | 406.4 ± 130.0  (*n*=6) | 410.2 ± 192.1  (*n*=6) | 590.4 ± 408.9  (*n*=6) | 418.9 ± 186.2  (*n*=5) | 547.0 ± 197.2  (*n*=6) | 423.4 ± 102.8  (*n*=6) | 441.6 ± 107.8  (*n*=6) | 551.8 ± 380.5  (*n*=6) | 410.5 ± 203.2  (*n*=5) |
|  |  | CA3 | 362.6 ± 165.8  (*n*=6) | 313.1 ± 128.9  (*n*=6) | 278.0 ± 169.6  (*n*=5) | 415.8 ± 248.8  (*n*=6) | 364.4 ± 213.9  (*n*=5) | 443.2 ± 211.5  (*n*=6) | 346.7 ± 124.3  (*n*=6) | 287.7 ± 151.3  (*n*=6) | 379.7 ± 182.3  (*n*=6) | 379.5 ± 230.5  (*n*=4) |
|  |  | CA1 | 378.7 ± 187.1  (*n*=5) | 269.0 ± 113.3  (*n*=6) | 250.0 ± 122.1  (*n*=6) | 358.2 ± 186.6  (*n*=6) | 314.6 ± 212.2  (*n*=4) | 481.4 ± 133.2  (*n*=4) | 280.3 ± 83.1  (*n*=6) | 297.0 ± 137.0  (*n*=5) | 345.8 ± 269.2  (*n*=6) | 371.1 ± 233.4  (*n*=4) |
|  | Int | DG | 452.1 ± 210.0  (*n*=6) | 405.2 ± 200.4  (*n*=6) | 365.6 ± 112.1  (*n*=6) | 446.0 ± 242.1  (*n*=6) | 411.5 ± 205.1  (*n*=5) | 419.2 ± 146.9  (*n*=6) | 440.4 ± 65.2  (*n*=6) | 417.6 ± 103.6  (*n*=6) | 410.5 ± 218.3  (*n*=6) | 386.4 ± 183.9  (*n*=5) |
|  |  | CA3 | 326.1 ± 146.6  (*n*=6) | 333.5 ± 140.9  (*n*=6) | 348.3 ± 110.2  (*n*=6) | 368.3 ± 199.1  (*n*=5) | 418.6 ± 174.8  (*n*=4) | 340.1 ± 114.6  (*n*=6) | 362.6 ± 75.6  (*n*=6) | 311.6 ± 107.2  (*n*=5) | 313.2 ± 156.0  (*n*=4) | 403.4 ± 197.7  (*n*=5) |
|  |  | CA1 | 286.1 ± 116.6  (*n*=4) | 203.8 ± 109.5  (*n*=6) | 239.8 ± 92.2  (*n*=5) | 257.2 ± 121.3  (*n*=6) | 274.9 ± 173.6  (*n*=4) | 277.3 ± 125.2  (*n*=5) | 209.8 ± 60.7  (*n*=6) | 236.9 ± 41.9  (*n*=5) | 295.4 ± 125.2  (*n*=5) | 282.4 ± 171.9  (*n*=4) |
|  | Ventral | DG | 437.2 ± 152.9  (*n*=5) | 552.0 ± 130.3  (*n*=5) | 542.9 ± 158.6  (*n*=6) | 697.2 ± 407.7  (*n*=4) | 527.4 ± 329.4  (*n*=4) | 425.3 ± 185.9  (*n*=5) | 445.0 ± 79.5  (*n*=6) | 435.2 ± 170.7  (*n*=5) | 535.6 ± 259.3  (*n*=6) | 419.8 ± 166.8  (*n*=5) |
|  |  | CA3 | 275.6 ± 84.6  (*n*=6) | 277.5 ± 62.1  (*n*=6) | 333.3 ± 87.6  (*n*=6) | 326.6 ± 153.5  (*n*=6) | 368.9 ± 206.9  (*n*=5) | 275.0 ± 142.4  (*n*=6) | 333.8 ± 123.9  (*n*=6) | 332.0 ± 117.9  (*n*=6) | 263.5 ± 135.9  (*n*=5) | 335.4 ± 205.0  (*n*=5) |
|  |  | CA1 | 241.3 ± 121.2  (*n*=6) | 228.0 ± 56.8  (*n*=6) | 211.4 ± 67.2  (*n*=6) | 290.7 ± 118.7  (*n*=6) | 321.3 ± 188.2  (*n*=5) | 216.8 ± 122.1  (*n*=6) | 242.3 ± 104.0  (*n*=6) | 229.8 ± 80.8  (*n*=6) | 341.1 ± 235.5  (*n*=6) | 341.2 ± 265.5  (*n*=5) |
|  | Ventral Pole | CA3 | 357.9 ± 160.5  (*n*=4) | - | 402.5 ± 109.5  (*n*=4) | 336.8 ± 203.1  (*n*=3) | - | 410.5 ± 129.2  (*n*=4) | 421.3 ± 102.8  (*n*=3) | 406.3 ± 115.6  (*n*=3) | 476.4 ± 145.6  (*n*=3) | 569.2 ± 96.1  (*n*=3) |
|  |  | CA1 | 291.4 ± 156.5  (*n*=6) | 273.2 ± 111.8  (*n*=5) | 255.6 ± 39.6  (*n*=6) | 416.0 ± 312.3  (*n*=5) | 416.4 ± 236.6  (*n*=5) | 373.3 ± 125.8  (*n*=6) | 300.1 ± 109.9  (*n*=6) | 237.1 ± 73.6  (*n*=5) | 398.8 ± 208.5  (*n*=5) | 395.7 ± 254.0  (*n*=5) |
| mPFC | Rostral | CG | 288.7 ± 156.5  (*n*=5) | 325.1 ± 161.2  (*n*=6) | 268.7 ± 148.6  (*n*=4) | 436.0 ± 280.3  (*n*=6) | 238.4 ± 101.1  (*n*=5) | 271.7 ± 123.1  (*n*=5) | 307.7 ± 132.7  (*n*=6) | 434.4 ± 145.7  (*n*=4) | 349.9 ± 110.4  (*n*=5) | 225.5 ± 97.5  (*n*=5) |
|  |  | PL | 248.9 ± 110.7  (*n*=5) | 282.1 ± 137.1  (*n*=6) | 272.1 ± 135.3  (*n*=5) | 336.1 ± 219.5  (*n*=6) | 267.2 ± 64.7  (*n*=5) | 272.1 ± 120.7  (*n*=5) | 262.1 ± 102.1  (*n*=6) | 289.5 ± 148.7  (*n*=6) | 445.2 ± 202.1  (*n*=6) | 251.2 ± 55.2  (*n*=5) |
|  |  | IL | 261.5 ± 113.6  (*n*=5) | 279.2 ± 133.7  (*n*=6) | 284.8 ± 161.5  (*n*=6) | 354.8 ± 176.3  (*n*=6) | 323.1 ± 78.4  (*n*=5) | 288.1 ± 139.3  (*n*=5) | 267.9 ± 88.2  (*n*=6) | 319.3 ±  175.8  (*n*=6) | 447.8 ± 183.0  (*n*=6) | 283.4 ± 27.9  (*n*=4) |
|  | Mid | CG | 195.5 ± 110.4  (*n*=6) | 256.2 ± 122.9  (*n*=6) | 271.1 ± 157.4  (*n*=5) | 363.2 ± 185.5  (*n*=5) | 269.5 ± 75.9  (*n*=5) | 255.2 ± 122.6  (*n*=5) | 312.0 ± 124.7  (*n*=6) | 239.1 ± 160.5  (*n*=5) | 282.4 ± 106.5  (*n*=5) | 303.8 ± 101.3  (*n*=5) |
|  |  | PL | 212.9 ± 106.5  (*n*=6) | 256.3 ± 143.8  (*n*=6) | 266.5 ± 140.9  (*n*=6) | 275.3 ± 165.3  (*n*=6) | 254.5 ± 79.6  (*n*=5) | 231.7 ± 103.7  (*n*=6) | 253.7 ± 156.6  (*n*=6) | 248.1 ± 148.3  (*n*=6) | 303.6 ± 186.1  (*n*=6) | 287.0 ± 95.8  (*n*=5) |
|  |  | IL | 219.9 ± 109.9  (*n*=6) | 241.5 ± 154.4  (*n*=6) | 298.1 ± 83.8  (*n*=4) | 239.6 ± 147.8  (*n*=6) | 249.2 ± 83.0  (*n*=5) | 226.7 ± 115.4  (*n*=6) | 144.4 ± 182.7  (*n*=6) | 235.8 ± 141.9  (*n*=6) | 245.4 ± 148.8  (*n*=6) | 251.6 ± 103.8  (*n*=5) |
|  | Caudal | CG | 258.6 ± 115.0  (*n*=5) | 277.8 ± 65.7  (*n*=6) | 319.9 ± 137.1  (*n*=6) | 385.9 ± 226.6  (*n*=6) | 347.4 ± 95.6  (*n*=4) | 224.2 ± 102.9  (*n*=6) | 324.6 ± 84.8  (*n*=6) | 281.3 ± 113.8  (*n*=5) | 297.8 ± 168.0  (*n*=6) | 320.3 ± 134.5  (*n*=4) |
|  |  | PL | 231.7 ± 138.7  (*n*=5) | 240.6 ± 92.8  (*n*=6) | 283.8 ± 141.2  (*n*=6) | 324.9 ± 164.4  (*n*=6) | 331.0 ± 90.5  (*n*=5) | 215.9 ± 109.4  (*n*=5) | 278.7 ± 88.8  (*n*=6) | 200.6 ± 84.4  (*n*=5) | 321.1 ± 160.2  (*n*=6) | 291.5 ± 90.0  (*n*=4) |
|  |  | IL | 241.2 ± 110.6  (*n*=6) | 257.4 ± 80.0  (*n*=6) | 235.1 ± 101.3  (*n*=6) | 335.4 ± 202.0  (*n*=6) | 349.5 ± 121.6  (*n*=5) | 205.5 ± 99.8  (*n*=6) | 296.0 ± 85.6  (*n*=6) | 256.9 ± 143.5  (*n*=6) | 296.1 ± 135.2  (*n*=6) | 307.5 ± 103.9  (*n*=5) |
| VPL Thalamus | | DL | - | - | - | - | - | - | - | - | - | - |
|  |  | VM | - | - | 239.5 ± 30.6  (*n*=3) | 398.5 ± 132.6  (*n*=3) | - | - | - | - | - | - |
|  |  | ZI | 350.9 ± 78.0  (*n*=3) | 262.5 ± 98.4  (*n*=3) | 233.6 ± 25.5  (*n*=4) | 358.6 ± 116.1  (*n*=4) | - | - | 241.3 ± 68.6  (*n*=3) | - | - | - |

**Supplementary Table B3.** Immunofluorescent intensity values of BDNF within NeuN+ areas of staining. Values represent group means ± standard deviation in arbitrary mean pixel intensity units. Results where *n* < 3 are excluded. Hipp: hippocampus; mPFC: medial prefrontal cortex; VPL: ventroposterior lateral nucleus; int: intermediate; DG: dentate gyrus; CG: cingulate gyrus; PL: prelimbic cortex; IL: infralimbic cortex; DL: dorsolateral; VM: ventromedial; ZI: zona incerta.

|  | | | Left (Contralateral) | | | | | Right (Ipsilateral) | | | | |
| --- | --- | --- | --- | --- | --- | --- | --- | --- | --- | --- | --- | --- |
| Region | AP Level | Subregion | Sham Vehicle | CCI Unaffected | CCI Affected | Sham Minocycline | CCI Minocycline | Sham Vehicle | CCI Unaffected | CCI Affected | Sham Minocycline | CCI Minocycline |
| Hipp | Dorsal | DG | 229.3 ± 132.6  (*n*=6) | 159.8 ± 62.5  (*n*=6) | 147.6 ± 70.4  (*n*=6) | 296.0 ± 212.9  (*n*=6) | 226.4 ± 134.0  (*n*=5) | 274.2 ± 143.8  (*n*=6) | 198.3 ± 60.0  (*n*=6) | 165.0 ± 52.6  (*n*=6) | 247.5 ± 202.8  (*n*=6) | 215.6 ± 126.5  (*n*=5) |
|  |  | CA3 | 221.2 ± 94.9  (*n*=6) | 207.9 ± 105.9  (*n*=6) | 153.9 ± 65.8  (*n*=5) | 263.9 ± 150.3  (*n*=6) | 249.3 ± 157.8  (*n*=5) | 260.6 ± 94.9  (*n*=6) | 236.1 ± 90.5  (*n*=6) | 160.8 ± 59.0  (*n*=6) | 250.0 ± 164.7  (*n*=6) | 292.6 ± 168.3  (*n*=4) |
|  |  | CA1 | 324.3 ± 137.0  (*n*=6) | 249.3 ± 114.6  (*n*=6) | 185.8 ± 66.4  (*n*=6) | 311.7 ± 146.9  (*n*=6) | 299.5 ± 215.4  (*n*=4) | 401.4 ± 83.9  (*n*=4) | 265.1 ± 74.5  (*n*=6) | 211.5 ± 69.1  (*n*=5) | 284.5 ± 183.7  (*n*=6) | 321.3 ± 208.9  (*n*=4) |
|  | Int | DG | 192.6 ± 87.5  (*n*=6) | 167.9 ± 85.5  (*n*=6) | 144.3 ± 57.8  (*n*=6) | 206.8 ± 129.4  (*n*=6) | 228.3 ± 116.3  (*n*=5) | 187.4 ± 64.6  (*n*=6) | 193.7 ± 20.7  (*n*=6) | 163.5 ± 42.5  (*n*=6) | 187.2 ± 132.8  (*n*=6) | 230.1 ± 134.6  (*n*=5) |
|  |  | CA3 | 245.4 ± 103.2  (*n*=6) | 224.4 ± 70.7  (*n*=6) | 218.5 ± 34.6  (*n*=6) | 249.0 ± 145.5  (*n*=5) | 313.9 ± 176.3  (*n*=4) | 239.5 ± 79.7  (*n*=6) | 241.0 ± 43.1  (*n*=6) | 196.9 ± 58.0  (*n*=5) | 207.6 ± 124.1  (*n*=4) | 290.0 ± 149.4  (*n*=5) |
|  |  | CA1 | 277.9 ± 122.6  (*n*=4) | 197.3 ± 86.2  (*n*=6) | 203.7 ± 19.3  (*n*=5) | 280.6 ± 85.6  (*n*=6) | 284.6 ± 161.2  (*n*=4) | 271.0 ± 91.8  (*n*=5) | 224.5 ± 77.4  (*n*=6) | 215.1 ± 37.2  (*n*=5) | 273.5 ± 102.0  (*n*=5) | 296.6 ± 162.7  (*n*=4) |
|  | Ventral | DG | 177.9 ± 106.6  (*n*=5) | 209.4 ± 40.5  (*n*=5) | 189.0 ± 31.4  (*n*=6) | 246.4 ± 117.1  (*n*=4) | 271.7 ± 84.3  (*n*=4) | 187.4 ± 124.6  (*n*=5) | 204.1 ± 58.1  (*n*=6) | 163.4 ± 28.1  (*n*=5) | 202.3 ± 140.5  (*n*=6) | 211.3 ± 115.8  (*n*=5) |
|  |  | CA3 | 224.7 ± 104.9  (*n*=6) | 201.5 ± 35.2  (*n*=6) | 234.7 ± 30.6  (*n*=6) | 267.6 ± 80.2  (*n*=6) | 250.5 ± 138.3  (*n*=5) | 207.0 ± 82.1  (*n*=6) | 224.1 ± 48.4  (*n*=6) | 211.4 ± 24.9  (*n*=6) | 191.6 ± 114.4  (*n*=5) | 240.4 ± 129.1  (*n*=5) |
|  |  | CA1 | 196.1 ± 96.7  (*n*=6) | 181.0 ± 47.7  (*n*=6) | 197.7 ± 21.6  (*n*=6) | 272.9 ± 90.3  (*n*=6) | 258.7 ± 156.5  (*n*=5) | 199.2 ± 83.0  (*n*=6) | 213.3 ± 66.6  (*n*=6) | 183.4 ± 26.7  (*n*=6) | 280.3 ± 143.6  (*n*=6) | 272.9 ± 162.3  (*n*=5) |
|  | Ventral Pole | CA3 | 235.0 ± 90.9  (*n*=4) | - | 220.4 ± 9.1  (*n*=4) | 247.4 ± 104.1  (*n*=3) | - | 259.0 ± 78.1  (*n*=4) | 269.6 ± 43.0  (*n*=3) | 212.4 ± 36.7  (*n*=3) | 347.4 ± 154.0  (*n*=3) | 397.2 ± 117.4  (*n*=3) |
|  |  | CA1 | 222.5 ± 101.2  (*n*=6) | 232.2 ± 91.7  (*n*=5) | 189.7 ± 37.6  (*n*=6) | 310.5 ± 223.6  (*n*=5) | 336.8 ± 176.2  (*n*=5) | 248.9 ± 65.0  (*n*=6) | 240.5 ± 54.1  (*n*=6) | 181.8 ± 43.1  (*n*=5) | 309.5 ± 138.7  (*n*=5) | 301.4 ± 167.9  (*n*=5) |
| mPFC | Rostral | CG | 122.0 ± 86.4  (*n*=5) | 169.1 ± 90.0  (*n*=6) | 107.0 ± 52.4  (*n*=4) | 213.0 ± 152.0  (*n*=6) | 142.1 ± 76.6  (*n*=5) | 119.2 ± 67.5  (*n*=5) | 157.9 ± 80.2  (*n*=6) | 190.9 ± 52.2  (*n*=4) | 180.8 ± 17.3  (*n*=5) | 105.5 ± 62.5  (*n*=5) |
|  |  | PL | 119.0 ± 64.3  (*n*=5) | 157.8 ± 81.5  (*n*=6) | 116.1 ± 66.8  (*n*=5) | 167.0 ± 126.4  (*n*=6) | 156.6 ± 44.4  (*n*=5) | 133.5 ± 75.7  (*n*=5) | 142.3 ± 59.0  (*n*=6) | 132.6 ± 48.3  (*n*=6) | 225.4 ± 85.7  (*n*=6) | 130.9 ± 35.2  (*n*=5) |
|  |  | IL | 122.2 ± 62.2  (*n*=5) | 163.4 ± 87.7  (*n*=6) | 121.9 ± 65.7  (*n*=6) | 169.0 ± 120.6  (*n*=6) | 189.6 ± 58.1  (*n*=5) | 141.8 ± 82.2  (*n*=5) | 155.3 ± 57.8  (*n*=6) | 137.8 ± 50.1  (*n*=6) | 227.9 ± 86.7  (*n*=6) | 151.4 ± 13.2  (*n*=4) |
|  | Mid | CG | 99.3 ± 65.8  (*n*=6) | 141.1 ± 82.2  (*n*=6) | 117.5 ± 78.7  (*n*=5) | 215.7 ± 118.8  (*n*=5) | 137.3 ± 30.7  (*n*=5) | 109.5 ± 53.1  (*n*=5) | 167.7 ± 90.8  (*n*=6) | 94.7 ± 59.0  (*n*=5) | 148.2 ± 74.1  (*n*=5) | 130.8 ± 45.4  (*n*=5) |
|  |  | PL | 102.5 ± 61.8  (*n*=6) | 138.4 ± 94.1  (*n*=6) | 112.3 ± 57.9  (*n*=6) | 131.4 ± 78.2  (*n*=6) | 139.3 ± 44.9  (*n*=5) | 108.6 ± 54.4  (*n*=6) | 130.4 ± 94.7  (*n*=6) | 113.9 ± 75.5  (*n*=6) | 136.6 ± 70.0  (*n*=6) | 148.5 ± 48.4  (*n*=5) |
|  |  | IL | 91.2 ± 45.9  (*n*=6) | 127.7 ± 84.3  (*n*=6) | 125.9 ± 48.2  (*n*=4) | 115.8 ± 76.9  (*n*=6) | 142.5 ± 52.0  (*n*=5) | 103.1 ± 56.5  (*n*=6) | 131.0 ± 89.1  (*n*=6) | 97.9 ± 60.6  (*n*=6) | 113.9 ± 62.2  (*n*=6) | 138.4 ± 47.5  (*n*=5) |
|  | Caudal | CG | 146.8 ± 76.3  (*n*=5) | 156.5 ± 49.3  (*n*=6) | 163.1 ± 86.6  (*n*=6) | 224.8 ± 145.4  (*n*=6) | 196.9 ± 52.5  (*n*=4) | 111.7 ± 55.5  (*n*=6) | 179.4 ± 72.7  (*n*=6) | 148.3 ± 86.7  (*n*=5) | 145.8 ± 82.9  (*n*=6) | 160.8 ± 59.1  (*n*=4) |
|  |  | PL | 128.9 ± 77.1  (*n*=5) | 143.9 ± 65.4  (*n*=6) | 148.8 ± 95.5  (*n*=6) | 172.7 ± 95.5  (*n*=6) | 177.9 ± 45.6  (*n*=5) | 117.0 ± 60.1  (*n*=5) | 158.1 ± 62.8  (*n*=6) | 92.9 ± 32.3  (*n*=5) | 165.6 ± 86.0  (*n*=6) | 170.2 ± 59.8  (*n*=4) |
|  |  | IL | 125.2 ± 62.9  (*n*=6) | 150.2 ± 57.9  (*n*=6) | 123.7 ± 64.8  (*n*=6) | 159.0 ± 82.7  (*n*=6) | 167.5 ± 43.3  (*n*=5) | 112.0 ± 57.2  (*n*=6) | 159.9 ± 64.4  (*n*=6) | 122.2 ± 85.9  (*n*=6) | 151.1 ± 73.5  (*n*=6) | 186.7 ± 81.5  (*n*=5) |
| VPL Thalamus | | DL | - | - | - | - | - | - | - | - | - | - |
|  |  | VM | - | - | 166.5 ± 15.4  (*n*=3) | 286.5 ± 91.8  (*n*=3) | - | - | - | - | - | - |
|  |  | ZI | 271.3 ± 80.4  (*n*=3) | 185.8 ± 86.9  (*n*=3) | 165.5 ± 16.4  (*n*=4) | 268.3 ± 83.3  (*n*=4) | - | - | 174.3 ± 83.4  (*n*=3) | - | - | - |

**Supplementary Table B4.** Immunofluorescent intensity values of CD206 within IBA1+ areas of staining. Values represent group means ± standard deviation in arbitrary mean pixel intensity units. Results where *n* < 3 are excluded. Hipp: hippocampus; mPFC: medial prefrontal cortex; VPL: ventroposterior lateral nucleus; int: intermediate; DG: dentate gyrus; CG: cingulate gyrus; PL: prelimbic cortex; IL: infralimbic cortex; DL: dorsolateral; VM: ventromedial; ZI: zona incerta.

|  | | | Left (Contralateral) | | | | | Right (Ipsilateral) | | | | |
| --- | --- | --- | --- | --- | --- | --- | --- | --- | --- | --- | --- | --- |
| Region | AP Level | Subregion | Sham Vehicle | CCI Unaffected | CCI Affected | Sham Minocycline | CCI Minocycline | Sham Vehicle | CCI Unaffected | CCI Affected | Sham Minocycline | CCI Minocycline |
| Hipp | Dorsal | DG | 737.1 ± 190.9  (*n*=6) | 766.2 ± 254.0  (*n*=6) | 750.6 ± 283.3  (*n*=5) | 684.2 ± 257.7  (*n*=6) | 705.2 ± 247.7  (*n*=6) | 770.1 ± 257.7  (*n*=6) | 677.7 ± 236.2  (*n*=6) | 776.4 ± 338.3  (*n*=5) | 684.4 ± 223.2  (*n*=6) | 531.3 ± 182.2  (*n*=6) |
|  |  | CA3 | 674.6 ± 264.5  (*n*=6) | 634.9 ± 250.7  (*n*=6) | 696.2 ± 287.6  (*n*=5) | 584.5 ± 200.5  (*n*=6) | 639.2 ± 348.9  (*n*=6) | 732.4 ± 246.3  (*n*=6) | 535.2 ± 259.3  (*n*=6) | 637.8 ± 397.5  (*n*=5) | 587.4 ± 194.9  (*n*=6) | 491.0 ± 343.2  (*n*=6) |
|  |  | CA1 | 646.5 ± 193.0  (*n*=6) | 642.2 ± 249.1  (*n*=6) | 656.3 ± 330.1  (*n*=5) | 586.3 ± 205.4  (*n*=6) | 603.7 ± 297.6  (*n*=6) | 654.9 ± 198.8  (*n*=6) | 546.1 ± 214.5  (*n*=6) | 699.1 ± 293.2  (*n*=5) | 592.9 ± 211.4  (*n*=6) | 482.0 ± 266.7  (*n*=6) |
|  | Int | DG | 659.1 ± 171.3  (*n*=6) | 640.2 ± 139.8  (*n*=6) | 702.6 ± 394.8  (*n*=6) | 667.8 ± 184.7  (*n*=6) | 659.4 ± 229.6  (*n*=6) | 649.4 ± 192.3  (*n*=6) | 614.0 ± 180.7  (*n*=6) | 682.2 ± 352.1  (*n*=6) | 612.7 ± 168.8  (*n*=6) | 621.9 ± 367.8  (*n*=6) |
|  |  | CA3 | 545.3 ± 150.5  (*n*=6) | 533.0 ± 146.1  (*n*=6) | 592.2 ± 365.5  (*n*=6) | 529.2 ± 209.2  (*n*=6) | 506.0 ± 211.3  (*n*=6) | 565.5 ± 123.3  (*n*=6) | 485.5 ± 112.3  (*n*=6) | 593.5 ± 431.9  (*n*=6) | 497.3 ± 215.9  (*n*=6) | 468.9 ± 266.4  (*n*=6) |
|  |  | CA1 | 515.1 ± 150.0  (*n*=6) | 512.6 ± 94.7  (*n*=6) | 522.2 ± 331.7  (*n*=6) | 497.5 ± 160.4  (*n*=6) | 447.4 ± 197.7  (*n*=6) | 533.3 ± 154.8  (*n*=6) | 420.7 ± 133.5  (*n*=6) | 524.2 ± 299.0  (*n*=6) | 513.6 ± 107.9  (*n*=6) | 422.1 ± 208.0  (*n*=6) |
|  | Ventral | DG | 598.0 ± 177.9  (*n*=6) | 789.7 ± 343.8  (*n*=6) | 860.5 ± 501.6  (*n*=6) | 620.4 ± 328.6  (*n*=6) | 612.4 ± 261.4  (*n*=6) | 733.1 ± 123.1  (*n*=6) | 698.5 ± 305.1  (*n*=6) | 875.9 ± 390.8  (*n*=5) | 612.0 ± 283.8  (*n*=6) | 553.8 ± 335.3  (*n*=6) |
|  |  | CA3 | 490.6 ± 224.4  (*n*=6) | 568.0 ± 313.6  (*n*=6) | 785.1 ± 606.0  (*n*=6) | 544.6 ± 354.8  (*n*=6) | 505.5 ± 268.7  (*n*=6) | 623.0 ± 170.8  (*n*=6) | 493.9 ± 205.4  (*n*=6) | 768.8 ± 402.7  (*n*=5) | 446.9 ± 229.5  (*n*=6) | 465.1 ± 362.3  (*n*=6) |
|  |  | CA1 | 411.0 ± 151.9  (*n*=6) | 436.5 ± 204.3  (*n*=6) | 630.6 ± 477.4  (*n*=6) | 474.7 ± 321.3  (*n*=6) | 357.7 ± 226.4  (*n*=6) | 536.3 ± 144.2  (*n*=6) | 433.0 ± 187.2  (*n*=6) | 649.9 ± 356.9  (*n*=5) | 415.3 ± 232.8  (*n*=6) | 382.1 ± 373.9  (*n*=6) |
|  | Ventral Pole | CA3 | 594.3 ± 191.1  (*n*=6) | 577.8 ± 169.0  (*n*=6) | 539.8 ± 275.5  (*n*=6) | 552.6 ± 245.6  (*n*=6) | 467.6 ± 197.4  (*n*=6) | 537.2 ± 114.4  (*n*=6) | 544.7 ± 287.6  (*n*=6) | 639.7 ± 428.4  (*n*=6) | 544.4 ± 220.9  (*n*=6) | 513.0 ± 312.3  (*n*=6) |
|  |  | CA1 | 503.8 ± 244.8  (*n*=6) | 562.9 ± 172.1  (*n*=6) | 512.5 ± 257.8  (*n*=6) | 564.9 ± 230.4  (*n*=6) | 443.8 ± 227.9  (*n*=6) | 479.5 ± 159.7  (*n*=6) | 485.3 ± 224.2  (*n*=6) | 584.6 ± 354.2  (*n*=6) | 484.6 ± 180.2  (*n*=6) | 406.4 ± 265.7  (*n*=6) |
| mPFC | Rostral | CG | - | - | - | - | - | - | - | - | - | - |
|  |  | PL | - | - | - | - | - | - | - | - | - | - |
|  |  | IL | - | - | - | - | - | - | - | - | - | - |
|  | Mid | CG | - | - | - | - | - | - | - | - | - | - |
|  |  | PL | - | - | - | - | - | - | - | - | - | - |
|  |  | IL | - | - | - | - | - | - | - | - | - | - |
|  | Caudal | CG | - | - | - | - | - | - | - | - | - | - |
|  |  | PL | - | - | - | - | - | - | - | - | - | - |
|  |  | IL | - | - | - | - | - | - | - | - | - | - |
| VPL Thalamus | | DL | 287.5 ± 205.8  (*n*=6) | 199.1 ± 101.6  (*n*=6) | 257.0 ± 157.7  (*n*=5) | 296.4 ± 157.9  (*n*=6) | 245.1 ± 154.7  (*n*=5) | 384.3 ± 166.5  (*n*=6) | 260.2 ± 113.3  (*n*=6) | 275.3 ± 162.6  (*n*=5) | 331.2 ± 142.9  (*n*=5) | 200.3 ± 134.8  (*n*=5) |
|  |  | VM | 181.6 ± 110.6  (*n*=6) | 154.8 ± 59.5  (*n*=6) | 207.5 ± 104.3  (*n*=5) | 248.7 ± 142.0  (*n*=6) | 154.5 ± 69.5  (*n*=5) | 307.9 ± 153.0  (*n*=6) | 195.6 ± 67.3  (*n*=6) | 270.1 ± 172.2  (*n*=5) | 217.4 ± 128.3  (*n*=6) | 150.4 ± 78.7  (*n*=5) |
|  |  | ZI | 195.5 ± 107.7  (*n*=6) | 196.0 ± 105.2  (*n*=6) | 233.0 ± 140.2  (*n*=5) | 236.4 ± 92.2  (*n*=6) | 209.0 ± 145.5  (*n*=5) | 301.6 ± 163.6  (*n*=6) | 191.3 ± 85.0  (*n*=6) | 264.1 ± 166.6  (*n*=5) | 225.6 ± 104.4  (*n*=6) | 220.3 ± 138.6  (*n*=4) |

**Supplementary Table B5.** Immunofluorescent intensity values of CD206 within NeuN+ areas of staining. Values represent group means ± standard deviation in arbitrary mean pixel intensity units. Results where *n* < 3 are excluded. Hipp: hippocampus; mPFC: medial prefrontal cortex; VPL: ventroposterior lateral nucleus; int: intermediate; DG: dentate gyrus; CG: cingulate gyrus; PL: prelimbic cortex; IL: infralimbic cortex; DL: dorsolateral; VM: ventromedial; ZI: zona incerta.

|  | | | Left (Contralateral) | | | | | Right (Ipsilateral) | | | | |
| --- | --- | --- | --- | --- | --- | --- | --- | --- | --- | --- | --- | --- |
| Region | AP Level | Subregion | Sham Vehicle | CCI Unaffected | CCI Affected | Sham Minocycline | CCI Minocycline | Sham Vehicle | CCI Unaffected | CCI Affected | Sham Minocycline | CCI Minocycline |
| Hipp | Dorsal | DG | 480.1 ± 128.0  (*n*=6) | 505.0 ± 133.2  (*n*=6) | 469.4 ± 193.2  (*n*=5) | 493.7 ± 174.2  (*n*=6) | 454.9 ± 165.2  (*n*=6) | 506.3 ± 147.7  (*n*=6) | 450.4 ± 82.6  (*n*=6) | 482.8 ± 214.2  (*n*=5) | 483.9 ± 177.9  (*n*=6) | 416.1 ± 174.5  (*n*=6) |
|  |  | CA3 | 541.7 ± 176.7  (*n*=6) | 542.9 ± 144.0  (*n*=6) | 444.6 ± 132.9  (*n*=5) | 546.4 ± 172.7  (*n*=6) | 510.0 ± 178.3  (*n*=6) | 592.3 ± 161.6  (*n*=6) | 456.4 ± 134.2  (*n*=6) | 474.0 ± 178.0  (*n*=5) | 521.2 ± 214.4  (*n*=6) | 430.3 ± 253.2  (*n*=6) |
|  |  | CA1 | 631.2 ± 207.3  (*n*=6) | 639.4 ± 135.1  (*n*=6) | 669.3 ± 320.3  (*n*=5) | 627.1 ± 220.1  (*n*=6) | 589.8 ± 242.3  (*n*=6) | 666.1 ± 192.7  (*n*=6) | 570.2 ± 64.8  (*n*=6) | 686.6 ± 277.7  (*n*=5) | 627.8 ± 215.3  (*n*=6) | 519.4 ± 294.0  (*n*=6) |
|  | Int | DG | 392.3 ± 105.4  (*n*=6) | 422.7 ± 42.4  (*n*=6) | 398.3 ± 165.5  (*n*=6) | 468.5 ± 189.0  (*n*=6) | 405.8 ± 145.7  (*n*=6) | 415.9 ± 99.8  (*n*=6) | 379.2 ± 52.5  (*n*=6) | 437.8 ± 222.5  (*n*=6) | 451.1 ± 142.9  (*n*=6) | 406.7 ± 196.2  (*n*=6) |
|  |  | CA3 | 403.4 ± 162.4  (*n*=6) | 455.2 ± 47.9  (*n*=6) | 446.5 ± 251.0  (*n*=6) | 543.9 ± 205.2  (*n*=6) | 441.1 ± 167.0  (*n*=6) | 487.9 ± 136.1  (*n*=6) | 430.0 ± 62.3  (*n*=6) | 480.5 ± 248.0  (*n*=6) | 502.3 ± 216.4  (*n*=6) | 425.9 ± 208.7  (*n*=6) |
|  |  | CA1 | 458.4 ± 131.3  (*n*=6) | 449.9 ± 120.4  (*n*=6) | 477.3 ± 279.0  (*n*=6) | 573.0 ± 230.9  (*n*=6) | 461.9 ± 218.0  (*n*=6) | 472.0 ± 117.9  (*n*=6) | 424.4 ± 115.1  (*n*=6) | 497.4 ± 276.7  (*n*=6) | 555.4 ± 186.3  (*n*=6) | 432.3 ± 237.0  (*n*=6) |
|  | Ventral | DG | 440.1 ± 154.7  (*n*=6) | 467.7 ± 176.5  (*n*=6) | 534.1 ± 285.1  (*n*=6) | 523.9 ± 377.7  (*n*=6) | 382.2 ± 119.3  (*n*=6) | 509.5 ± 129.2  (*n*=6) | 461.2 ± 201.3  (*n*=6) | 547.7 ± 240.5  (*n*=5) | 492.6 ± 211.9  (*n*=6) | 388.3 ± 228.5  (*n*=6) |
|  |  | CA3 | 376.3 ± 137.6  (*n*=6) | 442.3 ± 154.6  (*n*=6) | 529.5 ± 355.3  (*n*=6) | 487.2 ± 364.2  (*n*=6) | 396.9 ± 214.1  (*n*=6) | 449.0 ± 141.0  (*n*=6) | 387.4 ± 50.7  (*n*=6) | 519.4 ± 248.4  (*n*=5) | 413.9 ± 221.6  (*n*=6) | 404.5 ± 368.0  (*n*=6) |
|  |  | CA1 | 385.6 ± 150.9  (*n*=6) | 371.6 ± 148.5  (*n*=6) | 511.3 ± 310.6  (*n*=6) | 526.7 ± 375.2  (*n*=6) | 385.7 ± 181.7  (*n*=6) | 474.4 ± 115.4  (*n*=6) | 340.7 ± 120.5  (*n*=6) | 476.7 ± 201.3  (*n*=5) | 495.5 ± 255.8  (*n*=6) | 355.1 ± 342.7  (*n*=6) |
|  | Ventral Pole | CA3 | 506.6 ± 154.5  (*n*=6) | 483.4 ± 93.8  (*n*=6) | 466.4 ± 222.9  (*n*=6) | 537.5 ± 245.1  (*n*=6) | 438.4 ± 190.1  (*n*=6) | 458.0 ± 98.8  (*n*=6) | 551.8 ± 356.2  (*n*=6) | 502.3 ± 258.7  (*n*=6) | 526.0 ± 234.2  (*n*=6) | 430.8 ± 249.8  (*n*=6) |
|  |  | CA1 | 453.5 ± 203.3  (*n*=6) | 514.5 ± 133.9  (*n*=6) | 459.0 ± 204.1  (*n*=6) | 532.9 ± 208.8  (*n*=6) | 451.1 ± 235.4  (*n*=6) | 424.9 ± 132.9  (*n*=6) | 421.3 ± 155.5  (*n*=6) | 473.8 ± 186.1  (*n*=6) | 470.6 ± 211.0  (*n*=6) | 370.2 ± 257.4  (*n*=6) |
| mPFC | Rostral | CG | - | - | - | - | - | - | - | - | - | - |
|  |  | PL | - | - | - | - | - | - | - | - | - | - |
|  |  | IL | - | - | - | - | - | - | - | - | - | - |
|  | Mid | CG | - | - | - | - | - | - | - | - | - | - |
|  |  | PL | - | - | - | - | - | - | - | - | - | - |
|  |  | IL | - | - | - | - | - | - | - | - | - | - |
|  | Caudal | CG | - | - | - | - | - | - | - | - | - | - |
|  |  | PL | - | - | - | - | - | - | - | - | - | - |
|  |  | IL | - | - | - | - | - | - | - | - | - | - |
| VPL Thalamus | | DL | 214.5 ± 137.4  (*n*=6) | 185.9 ± 49.4  (*n*=6) | 239.3 ± 155.4  (*n*=5) | 280.7 ± 70.0  (*n*=6) | 225.9 ± 131.7  (*n*=5) | 263.8 ± 156.4  (*n*=6) | 258.7 ± 87.6  (*n*=6) | 235.4 ± 136.4  (*n*=5) | 298.1 ± 74.3  (*n*=5) | 220.5 ± 103.0  (*n*=5) |
|  |  | VM | 241.6 ± 154.0  (*n*=6) | 212.1 ± 59.8  (*n*=6) | 247.3 ± 165.2  (*n*=5) | 286.8 ± 75.5  (*n*=6) | 270.9 ± 152.9  (*n*=5) | 281.4 ± 157.3  (*n*=6) | 291.5 ± 114.0  (*n*=6) | 232.6 ± 141.3  (*n*=5) | 306.4 ± 49.1  (*n*=6) | 252.4 ± 186.0  (*n*=5) |
|  |  | ZI | 216.6 ± 137.4  (*n*=6) | 227.8 ± 78.1  (*n*=6) | 207.0 ± 133.1  (*n*=5) | 254.8 ± 68.8  (*n*=6) | 255.4 ± 153.7  (*n*=5) | 246.2 ± 137.3  (*n*=6) | 263.5 ± 98.3  (*n*=6) | 229.6 ± 136.9  (*n*=5) | 275.2 ± 56.3  (*n*=6) | 291.8 ± 188.2  (*n*=4) |

**Supplementary Table B6.** Immunofluorescent intensity values of IL-1β within IBA1+ areas of staining. Values represent group means ± standard deviation in arbitrary mean pixel intensity units. Results where *n* < 3 are excluded. Hipp: hippocampus; mPFC: medial prefrontal cortex; VPL: ventroposterior lateral nucleus; int: intermediate; DG: dentate gyrus; CG: cingulate gyrus; PL: prelimbic cortex; IL: infralimbic cortex; DL: dorsolateral; VM: ventromedial; ZI: zona incerta.

|  | | | Left (Contralateral) | | | | | Right (Ipsilateral) | | | | |
| --- | --- | --- | --- | --- | --- | --- | --- | --- | --- | --- | --- | --- |
| Region | AP Level | Subregion | Sham Vehicle | CCI Unaffected | CCI Affected | Sham Minocycline | CCI Minocycline | Sham Vehicle | CCI Unaffected | CCI Affected | Sham Minocycline | CCI Minocycline |
| Hipp | Dorsal | DG | 673.2 ± 157.8  (*n*=6) | 735.6 ± 221.2  (*n*=6) | 593.4 ± 239.2  (*n*=6) | 688.8 ± 131.8  (*n*=6) | 567.7 ± 254.6  (*n*=6) | 648.0 ± 184.5  (*n*=6) | 608.3 ± 197.9  (*n*=6) | 599.1 ± 211.1  (*n*=6) | 725.0 ± 127.3  (*n*=6) | 568.8 ± 250.9  (*n*=6) |
|  |  | CA3 | 514.7 ± 141.0  (*n*=6) | 460.8 ± 149.7  (*n*=6) | 503.3 ± 190.8  (*n*=6) | 571.9 ± 117.7  (*n*=6) | 481.6 ± 305.5  (*n*=5) | 539.0 ± 121.6  (*n*=6) | 437.2 ± 150.9  (*n*=6) | 482.4 ± 163.7  (*n*=6) | 587.8 ± 177.1  (*n*=6) | 383.1 ± 235.3  (*n*=6) |
|  |  | CA1 | 443.6 ± 110.9  (*n*=6) | 417.2 ± 109.1  (*n*=6) | 413.2 ± 182.3  (*n*=6) | 471.7 ± 115.1  (*n*=6) | 421.1 ± 246.5  (*n*=5) | 453.5 ± 91.9  (*n*=6) | 381.7 ± 117.9  (*n*=6) | 414.5 ± 188.9  (*n*=6) | 505.1 ± 73.5  (*n*=6) | 357.2 ± 219.8  (*n*=6) |
|  | Int | DG | 592.7 ± 59.5  (*n*=6) | 640.5 ± 285.4  (*n*=6) | 578.7 ± 204.8  (*n*=6) | 496.0 ± 110.3  (*n*=6) | 433.1 ± 72.9  (*n*=6) | 520.2 ± 152.4  (*n*=6) | 531.4 ± 146.1  (*n*=6) | 542.1 ± 189.7  (*n*=6) | 569.6 ± 153.6  (*n*=6) | 437.9 ± 93.7  (*n*=6) |
|  |  | CA3 | 449.9 ± 136.9  (*n*=6) | 353.8 ± 33.7  (*n*=6) | 400.9 ± 142.9  (*n*=6) | 358.8 ± 44.6  (*n*=6) | 327.4 ± 80.5  (*n*=6) | 378.9 ± 97.4  (*n*=6) | 359.4 ± 69.3  (*n*=6) | 388.4 ± 130.9  (*n*=6) | 465.2 ± 211.1  (*n*=6) | 332.1 ± 103.2  (*n*=6) |
|  |  | CA1 | 363.4 ± 68.1  (*n*=6) | 354.7 ± 102.2  (*n*=6) | 378.5 ± 164.6  (*n*=6) | 344.7 ± 79.0  (*n*=6) | 312.9 ± 83.0  (*n*=6) | 359.1 ± 75.0  (*n*=6) | 337.4 ± 88.9  (*n*=6) | 399.9 ± 170.5  (*n*=6) | 359.3 ± 70.3  (*n*=6) | 306.3 ± 80.2  (*n*=6) |
|  | Ventral | DG | 370.0 ± 110.2  (*n*=6) | 445.7 ± 170.6  (*n*=6) | 379.2 ± 101.8  (*n*=6) | 313.8 ± 63.9  (*n*=5) | 340.6 ± 129.2  (*n*=5) | 412.5 ± 58.1  (*n*=6) | 319.7 ± 108.2  (*n*=6) | 403.1 ± 170.5  (*n*=6) | 439.6 ± 116.4  (*n*=6) | 393.9 ± 176.9  (*n*=5) |
|  |  | CA3 | 331.9 ± 117.3  (*n*=6) | 341.3 ± 79.8  (*n*=6) | 366.5 ± 130.2  (*n*=6) | 320.1 ± 47.8  (*n*=5) | 309.7 ± 62.7  (*n*=5) | 357.0 ± 55.6  (*n*=6) | 288.8 ± 108.1  (*n*=6) | 365.0 ± 163.9  (*n*=5) | 382.1 ± 106.7  (*n*=6) | 305.5 ± 128.7  (*n*=5) |
|  |  | CA1 | 294.1 ± 116.3  (*n*=6) | 262.0 ± 81.8  (*n*=6) | 276.7 ± 91.4  (*n*=6) | 278.9 ± 67.2  (*n*=5) | 255.7 ± 88.6  (*n*=5) | 311.2 ± 76.1  (*n*=6) | 233.1 ± 89.4  (*n*=6) | 290.5 ± 122.9  (*n*=5) | 354.0 ± 144.1  (*n*=6) | 289.5 ± 156.5  (*n*=5) |
|  | Ventral Pole | CA3 | 416.3 ± 121.0  (*n*=6) | 322.9 ± 74.1  (*n*=6) | 362.8 ± 99.9  (*n*=6) | 345.1 ± 61.7  (*n*=6) | 297.6 ± 67.2  (*n*=6) | 370.5 ± 108.6  (*n*=5) | 322.3 ± 106.6  (*n*=6) | 375.7 ± 90.0  (*n*=6) | 403.6 ± 115.2  (*n*=5) | 299.9 ± 103.2  (*n*=6) |
|  |  | CA1 | 379.8 ± 125.1  (*n*=6) | 328.7 ± 123.8  (*n*=6) | 395.8 ± 171.5  (*n*=6) | 300.3 ± 62.3  (*n*=5) | 330.5 ± 112.6  (*n*=6) | 340.3 ± 79.7  (*n*=5) | 293.4 ± 112.2  (*n*=6) | 385.4 ± 164.7  (*n*=6) | 318.3 ± 55.7  (*n*=4) | 287.0 ± 112.3  (*n*=6) |
| mPFC | Rostral | CG | 542.4 ± 142.8  (*n*=6) | 587.9 ± 238.8  (*n*=6) | 716.4 ± 144.0  (*n*=5) | 556.3 ± 150.9  (*n*=6) | 661.9 ± 194.6  (*n*=6) | 514.2 ± 109.4  (*n*=6) | 585.0 ± 246.6  (*n*=6) | 624.2 ± 241.0  (*n*=6) | 562.3 ± 183.6  (*n*=6) | 718.3 ± 167.5  (*n*=6) |
|  |  | PL | 399.2 ± 170.5  (*n*=6) | 394.5 ± 88.1  (*n*=6) | 671.4 ± 208.1  (*n*=5) | 509.5 ± 202.2  (*n*=6) | 551.5 ± 132.1  (*n*=6) | 406.5 ± 161.3  (*n*=6) | 425.0 ± 246.6  (*n*=6) | 512.7 ± 243.8  (*n*=6) | 438.7 ± 69.6  (*n*=6) | 523.6 ± 211.2  (*n*=6) |
|  |  | IL | 377.2 ± 140.1  (*n*=6) | 423.8 ± 138.9  (*n*=6) | 493.4 ± 192.7  (*n*=6) | 504.1 ± 141.0  (*n*=6) | 535.9 ± 133.6  (*n*=6) | 467.3 ± 217.9  (*n*=6) | 349.5 ± 154.9  (*n*=6) | 465.3 ± 185.1  (*n*=6) | 456.8 ± 55.4  (*n*=6) | 507.5 ± 190.4  (*n*=6) |
|  | Mid | CG | 597.1 ± 244.8  (*n*=6) | 481.0 ± 127.9  (*n*=6) | 668.5 ± 272.2  (*n*=6) | 572.5 ± 53.6  (*n*=6) | 700.5 ± 240.6  (*n*=6) | 595.3 ± 134.6  (*n*=6) | 562.1 ± 335.3  (*n*=6) | 487.3 ± 184.0  (*n*=6) | 657.1 ± 156.1  (*n*=6) | 647.3 ± 172.9  (*n*=6) |
|  |  | PL | 424.7 ± 117.4  (*n*=6) | 380.5 ± 90.0  (*n*=6) | 527.0 ± 121.3  (*n*=6) | 578.9 ± 427.3  (*n*=6) | 439.5 ± 144.9  (*n*=6) | 418.4 ± 151.1  (*n*=6) | 436.6 ± 229.8  (*n*=6) | 396.7 ± 98.8  (*n*=6) | 397.8 ± 35.4  (*n*=6) | 518.8 ± 172.4  (*n*=6) |
|  |  | IL | 447.3 ± 252.5  (*n*=6) | 363.2 ± 95.6  (*n*=6) | 450.7 ± 57.5  (*n*=6) | 439.4 ± 170.1  (*n*=6) | 391.9 ± 53.1  (*n*=6) | 487.5 ± 243.5  (*n*=6) | 335.8 ± 93.8  (*n*=6) | 356.1 ± 81.7  (*n*=6) | 361.6 ± 40.8  (*n*=6) | 435.5 ± 155.2  (*n*=6) |
|  | Caudal | CG | 553.2 ± 140.9  (*n*=6) | 471.3 ± 153.4  (*n*=6) | 524.5 ± 256.1  (*n*=6) | 553.7 ± 44.2  (*n*=6) | 475.3 ± 110.5  (*n*=6) | 538.5 ± 132.7  (*n*=6) | 563.5 ± 181.6  (*n*=6) | 510.8 ± 60.6  (*n*=6) | 543.8 ± 54.8  (*n*=6) | 592.2 ± 103.7  (*n*=6) |
|  |  | PL | 450.5 ± 132.9  (*n*=6) | 367.4 ± 85.4  (*n*=6) | 443.0 ± 84.5  (*n*=6) | 448.8 ± 68.5  (*n*=6) | 406.4 ± 82.8  (*n*=6) | 442.4 ± 135.0  (*n*=6) | 376.0 ± 158.8  (*n*=6) | 427.4 ±  94.0  (*n*=6) | 431.0 ± 77.4  (*n*=6) | 417.7 ± 81.9  (*n*=6) |
|  |  | IL | 398.6 ± 181.8  (*n*=6) | 284.9 ± 79.6  (*n*=6) | 398.4 ± 113.0  (*n*=6) | 407.9 ± 58.1  (*n*=6) | 384.9 ± 109.6  (*n*=6) | 389.9 ± 139.2  (*n*=6) | 332.8 ± 154.9  (*n*=6) | 450.1 ± 65.3  (*n*=6) | 352.9 ± 91.6  (*n*=6) | 374.0 ± 149.9  (*n*=6) |
| VPL Thalamus | | DL | 406.0 ± 199.5  (*n*=6) | 423.7 ± 297.7  (*n*=3) | 410.8 ± 159.3  (*n*=5) | 484.1 ± 125.4  (*n*=6) | 384.6 ± 153.7  (*n*=5) | 396.9 ± 149.9  (*n*=6) | 421.8 ± 291.7  (*n*=3) | 422.2 ± 211.9  (*n*=5) | 541.1 ± 137.6  (*n*=6) | 434.2 ± 113.2  (*n*=5) |
|  |  | VM | 363.1 ± 183.0  (*n*=6) | 423.0 ± 298.6  (*n*=3) | 395.4 ± 178.4  (*n*=5) | 441.9 ± 141.1  (*n*=6) | 324.7 ± 166.4  (*n*=5) | 368.8 ± 174.6  (*n*=6) | 413.0 ± 302.3  (*n*=3) | 361.0 ± 242.2  (*n*=5) | 445.4 ± 124.1  (*n*=6) | 358.2 ± 143.1  (*n*=5) |
|  |  | ZI | 349.3 ± 164.5  (*n*=6) | 366.3 ± 253.4  (*n*=3) | 384.8 ± 87.8  (*n*=5) | 408.0 ± 94.0  (*n*=6) | 315.3 ± 134.3  (*n*=5) | 367.5 ± 174.5  (*n*=6) | 337.5 ± 214.2  (*n*=3) | 384.6 ± 158.2  (*n*=5) | 451.4 ± 116.9  (*n*=6) | 339.4 ± 137.4  (*n*=5) |

**Supplementary Table B7.** Immunofluorescent intensity values of IL-1β within NeuN+ areas of staining. Values represent group means ± standard deviation in arbitrary mean pixel intensity units. Results where *n* < 3 are excluded. Hipp: hippocampus; mPFC: medial prefrontal cortex; VPL: ventroposterior lateral nucleus; int: intermediate; DG: dentate gyrus; CG: cingulate gyrus; PL: prelimbic cortex; IL: infralimbic cortex; DL: dorsolateral; VM: ventromedial; ZI: zona incerta.

|  | | | Left (Contralateral) | | | | | Right (Ipsilateral) | | | | |
| --- | --- | --- | --- | --- | --- | --- | --- | --- | --- | --- | --- | --- |
| Region | AP Level | Subregion | Sham Vehicle | CCI Unaffected | CCI Affected | Sham Minocycline | CCI Minocycline | Sham Vehicle | CCI Unaffected | CCI Affected | Sham Minocycline | CCI Minocycline |
| Hipp | Dorsal | DG | 555.9 ± 179.1  (*n*=6) | 613.4 ± 238.1  (*n*=6) | 528.0 ± 209.5  (*n*=6) | 646.2 ± 230.7  (*n*=6) | 422.8 ± 178.9  (*n*=6) | 519.0 ± 187.0  (*n*=6) | 531.1 ± 254.3  (*n*=6) | 538.8 ± 156.7  (*n*=6) | 649.7 ± 231.1  (*n*=6) | 408.9 ± 184.1  (*n*=6) |
|  |  | CA3 | 328.0 ± 68.4  (*n*=6) | 361.0 ± 174.1  (*n*=6) | 347.1 ± 150.1  (*n*=6) | 427.2 ± 147.8  (*n*=6) | 352.3 ± 166.5  (*n*=5) | 376.0 ± 122.0  (*n*=6) | 346.1 ± 151.2  (*n*=6) | 354.0 ± 101.4  (*n*=6) | 415.1 ± 155.2  (*n*=6) | 261.9 ±  73.8  (*n*=6) |
|  |  | CA1 | 431.8 ± 179.4  (*n*=6) | 479.6 ± 282.1  (*n*=6) | 431.9 ± 276.9  (*n*=6) | 599.4 ± 294.3  (*n*=6) | 387.7 ± 208.7  (*n*=5) | 447.4 ± 192.3  (*n*=6) | 481.7 ± 281.8  (*n*=6) | 425.1 ± 179.7  (*n*=6) | 638.4 ± 294.1  (*n*=6) | 300.5 ± 95.7  (*n*=6) |
|  | Int | DG | 403.0 ± 70.5  (*n*=6) | 472.1 ± 284.6  (*n*=6) | 451.2 ± 137.1  (*n*=6) | 394.3 ± 186.7  (*n*=6) | 333.0 ± 101.8  (*n*=6) | 404.1 ± 146.8  (*n*=6) | 401.9 ± 179.5  (*n*=6) | 430.4 ± 128.3  (*n*=6) | ­482.6 ± 238.0  (*n*=6) | 332.7 ± 140.9  (*n*=6) |
|  |  | CA3 | 322.7 ± 96.4  (*n*=6) | 300.4 ± 120.0  (*n*=6) | 315.5 ± 106.0  (*n*=6) | 301.5 ± 107.6  (*n*=6) | 283.1 ± 115.6  (*n*=6) | 293.4 ± 84.8  (*n*=6) | 295.7 ± 146.6  (*n*=6) | 316.1 ± 90.6  (*n*=6) | 358.0 ± 160.3  (*n*=6) | 246.8 ± 81.3  (*n*=6) |
|  |  | CA1 | 318.8 ± 116.3  (*n*=6) | 371.9 ± 232.9  (*n*=6) | 338.9 ± 112.5  (*n*=6) | 380.0 ± 221.5  (*n*=6) | 314.6 ± 123.6  (*n*=6) | 329.6 ± 125.7  (*n*=6) | 351.3 ± 202.3  (*n*=6) | 352.7 ± 141.3  (*n*=6) | 403.5 ± 227.0  (*n*=6) | 304.5 ± 127.7  (*n*=6) |
|  | Ventral | DG | 281.6 ± 103.0  (*n*=6) | 371.3 ± 214.9  (*n*=6) | 311.2 ± 86.8  (*n*=6) | 236.2 ± 31.1  (*n*=5) | 240.7 ± 107.3  (*n*=5) | 338.2 ± 94.5  (*n*=6) | 248.2 ± 97.9  (*n*=6) | 345.9 ± 168.6  (*n*=6) | 411.7 ± 206.6  (*n*=6) | 284.2 ± 100.0  (*n*=5) |
|  |  | CA3 | 240.3 ± 115.2  (*n*=6) | 251.3 ± 78.5  (*n*=6) | 265.2 ± 97.9  (*n*=6) | 239.0 ± 55.8  (*n*=5) | 235.9 ± 66.3  (*n*=5) | 257.1 ± 85.5  (*n*=6) | 212.6 ± 86.3  (*n*=6) | 277.0 ± 113.1  (*n*=5) | 299.0 ± 115.4  (*n*=6) | 238.3 ± 113.4  (*n*=5) |
|  |  | CA1 | 252.2 ± 150.7  (*n*=6) | 217.5 ± 91.3  (*n*=6) | 259.9 ± 104.1  (*n*=6) | 231.8 ± 63.0  (*n*=5) | 227.8 ± 102.1  (*n*=5) | 264.2 ± 99.2  (*n*=6) | 186.3 ± 58.1  (*n*=6) | 283.1 ± 112.0  (*n*=5) | 330.3 ± 190.7  (*n*=6) | 251.8 ± 120.8  (*n*=5) |
|  | Ventral Pole | CA3 | 345.3 ± 126.3  (*n*=6) | 279.0 ± 115.3  (*n*=6) | 350.0 ± 91.8  (*n*=6) | 285.4 ± 47.7  (*n*=6) | 267.0 ± 111.9  (*n*=6) | 337.6 ± 165.1  (*n*=5) | 271.3 ± 105.2  (*n*=6) | 351.3 ± 117.4  (*n*=6) | 328.4 ± 129.8  (*n*=5) | 233.6 ± 101.5  (*n*=6) |
|  |  | CA1 | 323.9 ± 120.6  (*n*=6) | 275.7 ± 150.8  (*n*=6) | 364.7 ± 130.2  (*n*=6) | 235.3 ± 77.4  (*n*=5) | 296.8 ± 145.4  (*n*=6) | 311.5 ± 86.7  (*n*=5) | 235.7 ± 106.4  (*n*=6) | 369.1 ± 145.6  (*n*=6) | 271.4 ± 98.6  (*n*=4) | 230.4 ± 89.4  (*n*=6) |
| mPFC | Rostral | CG | 648.7 ± 240.5  (*n*=6) | 693.0 ± 354.7  (*n*=6) | 1102.5 ± 180.3  (*n*=5) | 721.3 ± 329.3  (*n*=6) | 849.0 ± 310.6  (*n*=6) | 713.0 ± 187.6  (*n*=6) | 688.7 ± 406.8  (*n*=6) | 905.8 ± 341.1  (*n*=6) | 690.3 ± 253.1  (*n*=6) | 936.3 ± 229.4  (*n*=6) |
|  |  | PL | 552.0 ± 238.3  (*n*=6) | 416.5 ± 140.6  (*n*=6) | 805.0 ± 258.9  (*n*=5) | 628.9 ± 375.8  (*n*=6) | 688.7 ±  138.8  (*n*=6) | 549.4 ± 178.5  (*n*=6) | 444.8 ± 259.8  (*n*=6) | 657.8 ± 321.7  (*n*=6) | 520.2 ± 63.0  (*n*=6) | 658.0 ± 297.4  (*n*=6) |
|  |  | IL | 553.5 ± 193.0  (*n*=6) | 449.1 ± 210.1  (*n*=6) | 620.4 ± 245.8  (*n*=6) | 643.2 ± 293.5  (*n*=6) | 706.3 ± 143.2  (*n*=6) | 603.6 ± 181.5  (*n*=6) | 360.3 ± 159.9  (*n*=6) | 563.3 ± 205.7  (*n*=6) | 559.0 ± 77.2  (*n*=6) | 646.9 ± 295.1  (*n*=6) |
|  | Mid | CG | 694.0 ± 288.9  (*n*=6) | 523.6 ± 232.3  (*n*=6) | 790.6 ± 301.6  (*n*=6) | 626.7 ± 116.4  (*n*=6) | 887.8 ± 238.8  (*n*=6) | 739.3 ± 239.8  (*n*=6) | 624.7 ± 370.7  (*n*=6) | 624.4 ± 248.0  (*n*=6) | 834.9 ± 284.9  (*n*=6) | 893.4 ± 298.7  (*n*=6) |
|  |  | PL | 510.1 ± 128.6  (*n*=6) | 383.4 ± 118.4  (*n*=6) | 580.1 ± 133.7  (*n*=6) | 623.7 ± 364.1  (*n*=6) | 532.1 ± 270.4  (*n*=6) | 476.0 ± 194.8  (*n*=6) | 466.2 ± 163.5  (*n*=6) | 469.6 ± 171.9  (*n*=6) | 462.1 ± 75.7  (*n*=6) | 661.9 ± 283.2  (*n*=6) |
|  |  | IL | 581.5 ± 372.3  (*n*=6) | 371.5 ± 106.9  (*n*=6) | 502.7 ± 159.5  (*n*=6) | 507.3 ± 267.9  (*n*=6) | 487.9 ± 85.6  (*n*=6) | 595.2 ± 301.9  (*n*=6) | 382.5 ± 122.2  (*n*=6) | 420.2 ± 167.4  (*n*=6) | 425.0 ± 85.6  (*n*=6) | 533.9 ±  183.7  (*n*=6) |
|  | Caudal | CG | 619.2 ± 275.9  (*n*=6) | 512.0 ± 162.2  (*n*=6) | 632.1 ± 272.1  (*n*=6) | 611.0 ± 144.1  (*n*=6) | 553.0 ± 137.5  (*n*=6) | 596.6 ± 238.5  (*n*=6) | 565.5 ± 224.9  (*n*=6) | 580.3 ± 112.2  (*n*=6) | 660.4 ± 131.9  (*n*=6) | 775.6 ± 130.3  (*n*=6) |
|  |  | PL | 547.7 ± 215.1  (*n*=6) | 426.3 ± 131.6  (*n*=6) | 532.8 ± 192.7  (*n*=6) | 577.2 ± 115.0  (*n*=6) | 536.9 ± 146.6  (*n*=6) | 544.5 ± 254.8  (*n*=6) | 431.4 ± 184.4  (*n*=6) | 561.5 ± 173.9  (*n*=6) | 573.6 ± 140.7  (*n*=6) | 535.0 ± 130.5  (*n*=6) |
|  |  | IL | 473.9 ± 295.5  (*n*=6) | 334.5 ± 136.2  (*n*=6) | 473.5 ± 224.8  (*n*=6) | 501.2 ± 124.6  (*n*=6) | 472.7 ± 133.1  (*n*=6) | 464.7 ± 239.2  (*n*=6) | 381.3 ± 182.7  (*n*=6) | 538.3 ± 203.2  (*n*=6) | 441.4 ± 136.9  (*n*=6) | 472.3 ± 159.4  (*n*=6) |
| VPL Thalamus | | DL | 379.3 ± 114.7  (*n*=6) | 475.2 ± 277.9  (*n*=3) | 366.8 ± 87.6  (*n*=5) | 433.7 ± 102.6  (*n*=6) | 267.3 ± 105.8  (*n*=5) | 369.9 ± 83.5  (*n*=6) | 448.7 ± 259.8  (*n*=3) | 346.8 ± 85.1  (*n*=5) | 472.4 ± 107.5  (*n*=6) | 400.9 ± 88.3  (*n*=5) |
|  |  | VM | 325.4 ± 71.1  (*n*=6) | 426.4 ± 248.7  (*n*=3) | 336.4 ± 79.2  (*n*=5) | 370.4 ± 58.1  (*n*=6) | 310.3 ± 87.6  (*n*=5) | 317.5 ± 55.3  (*n*=6) | 395.3 ± 231.7  (*n*=3) | 299.8 ± 82.4  (*n*=5) | 367.2 ± 72.5  (*n*=6) | 351.9 ± 113.9  (*n*=5) |
|  |  | ZI | 369.7 ± 71.3  (*n*=6) | 409.5 ± 243.2  (*n*=3) | 334.5 ± 48.3  (*n*=5) | 387.0 ± 75.7  (*n*=6) | 329.8 ± 80.3  (*n*=5) | 352.4 ± 58.3  (*n*=6) | 415.3 ± 224.7  (*n*=3) | 304.7 ± 80.3  (*n*=5) | 398.3 ± 72.5  (*n*=6) | 360.7 ± 92.1  (*n*=5) |

**Supplementary Table B8.** Immunofluorescent intensity values of phospho-p38 MAPK within IBA1+DAPI+ cells. Values represent group means ± standard deviation in arbitrary mean pixel intensity units. Results where *n* < 3 are excluded. Hipp: hippocampus; mPFC: medial prefrontal cortex; VPL: ventroposterior lateral nucleus; int: intermediate; DG: dentate gyrus; CG: cingulate gyrus; PL: prelimbic cortex; IL: infralimbic cortex; DL: dorsolateral; VM: ventromedial; ZI: zona incerta.

|  | | | Left (Contralateral) | | | | | Right (Ipsilateral) | | | | |
| --- | --- | --- | --- | --- | --- | --- | --- | --- | --- | --- | --- | --- |
| Region | AP Level | Subregion | Sham Vehicle | CCI Unaffected | CCI Affected | Sham Minocycline | CCI Minocycline | Sham Vehicle | CCI Unaffected | CCI Affected | Sham Minocycline | CCI Minocycline |
| Hipp | Dorsal | DG | 249.6 ± 175.2  (*n*=5) | 199.0 ± 113.6  (*n*=6) | 261.6 ± 185.2  (*n*=6) | 249.4 ± 181.8  (*n*=6) | 170.4 ± 71.1  (*n*=6) | 297.0 ± 173.6  (*n*=5) | 220.8 ± 143.4  (*n*=6) | 329.3 ± 233.7  (*n*=6) | 222.2 ± 115.0  (*n*=6) | 145.4 ± 35.8  (*n*=6) |
|  |  | CA3 | 211.8 ± 170.1  (*n*=5) | 125.8 ± 70.2  (*n*=6) | 173.7 ± 102.3  (*n*=6) | 154.6 ± 77.8  (*n*=6) | 176.8 ± 88.8  (*n*=6) | 169.9 ± 66.9  (*n*=5) | 126.1 ± 23.8  (*n*=6) | 167.3 ± 88.3  (*n*=6) | 127.6 ± 66.7  (*n*=6) | 108.4 ± 58.8  (*n*=6) |
|  |  | CA1 | 138.5 ± 69.3  (*n*=5) | 107.9 ± 32.9  (*n*=6) | 147.7 ± 93.1  (*n*=6) | 152.0 ± 91.0  (*n*=6) | 134.7 ± 46.7  (*n*=6) | 132.1 ± 55.6  (*n*=5) | 117.2 ± 37.3  (*n*=6) | 156.3 ± 66.5  (*n*=6) | 143.9 ±  64.4  (*n*=6) | 104.8 ± 54.4  (*n*=6) |
|  | Int | DG | 181.0 ± 84.4  (*n*=5) | 170.5 ± 53.2  (*n*=6) | 210.9 ± 73.5  (*n*=6) | 140.0 ± 77.6  (*n*=6) | 160.8 ± 63.4  (*n*=6) | 193.4 ± 87.8  (*n*=5) | 189.1 ± 54.2  (*n*=6) | 221.3 ± 129.5  (*n*=6) | 270.9 ± 282.6  (*n*=6) | 158.5 ± 80.9  (*n*=6) |
|  |  | CA3 | 174.4 ± 77.2  (*n*=5) | 121.9 ± 35.9  (*n*=6) | 180.1 ± 98.0  (*n*=6) | 84.1 ± 20.0  (*n*=6) | 154.1 ± 66.0  (*n*=6) | 153.7 ± 83.8  (*n*=5) | 132.3 ± 32.5  (*n*=6) | 127.5 ± 30.4  (*n*=6) | 222.6 ± 308.3  (*n*=6) | 140.5 ±  65.5  (*n*=6) |
|  |  | CA1 | 118.6 ± 82.0  (*n*=5) | 140.4 ± 62.3  (*n*=6) | 108.5 ± 35.5  (*n*=6) | 99.0 ± 57.9  (*n*=6) | 115.2 ± 42.0  (*n*=6) | 101.9 ± 26.2  (*n*=5) | 125.8 ± 54.3  (*n*=6) | 125.9 ± 30.7  (*n*=6) | 134.0 ± 57.9  (*n*=6) | 117.6 ± 62.9  (*n*=6) |
|  | Ventral | DG | 247.4 ± 172.0  (*n*=5) | 166.3 ± 60.7  (*n*=6) | 232.8 ± 148.0  (*n*=5) | 152.3 ± 85.1  (*n*=6) | 152.5 ± 45.0  (*n*=6) | 217.4 ± 113.6  (*n*=5) | 183.4 ±  102.5  (*n*=6) | 259.6 ± 132.4  (*n*=6) | 227.9 ±  205.9  (*n*=6) | 215.3 ± 167.8  (*n*=6) |
|  |  | CA3 | 139.6 ± 44.4  (*n*=5) | 127.5 ± 66.7  (*n*=6) | 157.9 ± 61.4  (*n*=5) | 82.4 ± 20.1  (*n*=6) | 115.6 ± 41.7  (*n*=6) | 168.2 ± 113.0  (*n*=5) | 120.4 ± 60.0  (*n*=6) | 109.8 ± 35.9  (*n*=6) | 145.9 ± 150.6  (*n*=6) | 112.5 ±  82.1  (*n*=6) |
|  |  | CA1 | 117.9 ± 30.0  (*n*=5) | 103.6 ± 25.4  (*n*=6) | 140.1 ± 49.6  (*n*=6) | 104.3 ± 26.9  (*n*=6) | 115.3 ± 43.0  (*n*=6) | 173.1 ± 117.3  (*n*=5) | 97.1 ± 28.6  (*n*=6) | 107.5 ± 45.8  (*n*=6) | 93.2 ± 38.3  (*n*=6) | 124.3 ± 76.1  (*n*=6) |
|  | Ventral Pole | CA3 | 231.1 ± 100.7  (*n*=5) | 169.8 ± 68.4  (*n*=6) | 183.5 ± 64.4  (*n*=6) | 121.8 ± 46.8  (*n*=6) | 247.1 ± 138.2  (*n*=6) | 206.1 ± 77.8  (*n*=5) | 154.4 ±  61.2  (*n*=6) | 209.7 ± 73.5  (*n*=6) | 208.2 ± 185.8  (*n*=6) | 174.7 ± 99.6  (*n*=6) |
|  |  | CA1 | 229.7 ± 202.1  (*n*=5) | 114.8 ± 31.8  (*n*=6) | 120.0 ± 56.5  (*n*=6) | 102.7 ± 52.5  (*n*=6) | 145.2 ± 62.6  (*n*=6) | 226.8 ± 267.3  (*n*=5) | 114.1 ± 31.7  (*n*=6) | 150.4 ± 80.5  (*n*=6) | 98.2 ± 41.0  (*n*=6) | 97.3 ± 23.1  (*n*=6) |
| mPFC | Rostral | CG | 444.3 ± 239.4  (*n*=6) | 202.2 ± 131.0  (*n*=7) | 384.7 ± 137.6  (*n*=6) | 265.4 ± 222.2  (*n*=6) | 571.4 ± 546.6  (*n*=6) | 381.0 ± 154.8  (*n*=6) | 234.8 ± 188.2  (*n*=7) | 484.3 ± 258.0  (*n*=6) | 287.6 ± 361.6  (*n*=6) | 404.4 ± 295.6  (*n*=6) |
|  |  | PL | 216.1 ± 70.5  (*n*=6) | 102.5 ± 23.4  (*n*=7) | 255.4 ± 89.8  (*n*=6) | 278.5 ± 365.3  (*n*=6) | 118.2 ± 53.9  (*n*=6) | 195.2 ± 80.7  (*n*=6) | 112.6 ± 41.9  (*n*=7) | 211.4 ± 67.3  (*n*=6) | 96.4 ± 50.8  (*n*=6) | 265.5 ± 251.4  (*n*=6) |
|  |  | IL | 187.0 ± 67.8  (*n*=6) | 94.9 ± 18.0  (*n*=7) | 211.2 ± 122.9  (*n*=6) | 182.4 ± 218.2  (*n*=6) | 163.2 ± 112.5  (*n*=6) | 197.2 ± 50.8  (*n*=6) | 94.7 ± 38.9  (*n*=7) | 149.9 ± 84.5  (*n*=6) | 78.8 ± 22.0  (*n*=6) | 219.8 ±  188.1  (*n*=6) |
|  | Mid | CG | 262.1 ± 207.7  (*n*=6) | 137.3 ± 37.2  (*n*=7) | 277.8 ± 277.9  (*n*=6) | 148.9 ± 113.3  (*n*=6) | 330.7 ± 188.1  (*n*=6) | 258.4 ± 142.9  (*n*=6) | 183.2 ± 56.8  (*n*=7) | 297.7 ± 266.0  (*n*=6) | 259.7 ± 248.2  (*n*=6) | 486.9 ± 559.3  (*n*=6) |
|  |  | PL | 132.1 ± 59.8  (*n*=6) | 103.1 ± 69.1  (*n*=7) | 187.6 ± 140.2  (*n*=6) | 101.5 ± 58.9  (*n*=6) | 126.0 ± 126.9  (*n*=6) | 167.3 ± 79.3  (*n*=6) | 111.6 ± 76.6  (*n*=7) | 132.7 ± 66.9  (*n*=6) | 74.8 ± 14.7  (*n*=6) | 128.1 ± 103.2  (*n*=6) |
|  |  | IL | 172.9 ± 113.5  (*n*=6) | 88.5 ± 33.3  (*n*=7) | 153.5 ± 90.4  (*n*=6) | 76.4 ± 26.1  (*n*=6) | 112.2 ± 75.6  (*n*=6) | 163.4 ± 99.6  (*n*=6) | 85.1 ± 39.4  (*n*=7) | 116.0 ± 39.1  (*n*=6) | 68.2 ± 26.1  (*n*=6) | 102.7 ±  44.7  (*n*=6) |
|  | Caudal | CG | 174.3 ± 127.1  (*n*=6) | 135.0 ± 44.9  (*n*=7) | 238.0 ± 259.8  (*n*=6) | 127.4 ± 81.8  (*n*=6) | 174.2 ± 125.4  (*n*=6) | 153.1 ± 132.3  (*n*=6) | 255.2 ± 192.0  (*n*=7) | 167.1 ±  89.4  (*n*=6) | 246.7 ± 361.4  (*n*=6) | 306.1 ± 212.3  (*n*=6) |
|  |  | PL | 154.6 ± 128.3  (*n*=6) | 82.2 ± 36.8  (*n*=7) | 137.3 ± 70.5  (*n*=6) | 77.4 ± 28.3  (*n*=6) | 129.1 ± 89.0  (*n*=6) | 145.0 ± 80.5  (*n*=6) | 96.8 ± 45.7  (*n*=7) | 124.4 ± 50.7  (*n*=6) | 61.2 ± 20.3  (*n*=6) | 148.1 ± 81.8  (*n*=6) |
|  |  | IL | 126.4 ± 78.9  (*n*=6) | 89.3 ± 53.8  (*n*=7) | 153.4 ± 106.0  (*n*=6) | 76.7 ± 21.9  (*n*=6) | 111.7 ± 52.2  (*n*=6) | 128.7 ± 43.1  (*n*=6) | 96.0 ± 36.5  (*n*=7) | 119.2 ± 70.6  (*n*=6) | 67.5 ± 20.6  (*n*=6) | 119.4 ± 50.9  (*n*=6) |
| VPL Thalamus | | DL | 295.4 ± 160.5  (*n*=6) | 203.1 ± 113.6  (*n*=6) | 252.9 ± 180.8  (*n*=5) | 302.9 ± 231.2  (*n*=6) | 289.0 ± 195.2  (*n*=6) | 271.7 ± 137.6  (*n*=6) | 263.9 ± 120.0  (*n*=6) | 290.5 ± 217.5  (*n*=5) | 277.4 ± 216.8  (*n*=6) | 231.2 ± 128.8  (*n*=6) |
|  |  | VM | 281.3 ± 151.4  (*n*=6) | 183.4 ± 56.7  (*n*=6) | 286.2 ± 212.6  (*n*=5) | 256.5 ± 174.6  (*n*=6) | 234.5 ± 146.4  (*n*=6) | 275.3 ± 173.9  (*n*=6) | 218.4 ± 156.4  (*n*=6) | 289.6 ± 229.9  (*n*=5) | 249.3 ± 176.3  (*n*=6) | 223.4 ± 113.6  (*n*=6) |
|  |  | ZI | 255.7 ± 176.3  (*n*=6) | 166.9 ± 67.3  (*n*=6) | 270.3 ± 309.1  (*n*=5) | 223.2 ± 161.1  (*n*=6) | 172.0 ± 85.3  (*n*=6) | 223.7 ± 150.8  (*n*=6) | 169.1 ± 56.6  (*n*=6) | 275.1 ± 199.1  (*n*=5) | 269.5 ± 200.5  (*n*=6) | 193.1 ± 77.9  (*n*=6) |
