# Supplementary material for: Minocycline Abrogates Individual Differences in Nerve Injury-Evoked Affective Disturbances in Male Rats and Prevents Associated Supraspinal Neuroinflammation
Source: J Neuroimmune Pharmacol. 2024 Jun 15;19(1):30. doi: 10.1007/s11481-024-10132-y (PMC11180027; doi:10.1007/s11481-024-10132-y)
Supplement: Supplementary file 3 — Supplementary Material 3 [file 11481_2024_10132_MOESM3_ESM.docx]

**Supplementary File C. Morphology measures and *n*-values (by anatomical region of interest and experimental group).**

**Supplementary Table C1**. Area values from fractal morphological analysis of IBA1+ microglia. Values represent group means ± standard deviation (SD) in pixels. The mean value from all microglia measured within the field of view was used to calculate the individual value for each animal. These values were then used to compute means and SD within experimental groups (i.e. *n* refers to biological, rather than technical, replicates). Hippocampal microglia have larger pixel area values due to higher image resolution compared to mPFC images. Results where *n* < 3 are excluded. Hipp: hippocampus; mPFC: medial prefrontal cortex; VPL: ventroposterior lateral nucleus; DG: dentate gyrus; CG: cingulate gyrus; PL: prelimbic cortex; IL: infralimbic cortex; DL: dorsolateral; VM: ventromedial; ZI: zona incerta. *: Sham vehicle vs CCI affected; $: Sham vehicle vs CCI unaffected; ^: Sham minocycline vs CCI minocycline; @: CCI unaffected vs CCI minocycline; #: CCI affected vs CCI minocycline; &: CCI unaffected vs CCI affected; +: Sham vehicle vs Sham minocycline. **P_adj_* < 0.05*; ** P_adj_* < 0.01.

|  | | | Left (Contralateral) | | | | | Right (Ipsilateral) | | | | |
| --- | --- | --- | --- | --- | --- | --- | --- | --- | --- | --- | --- | --- |
| Region | AP Level | Subregion | Sham Vehicle | CCI Unaffected | CCI Affected | Sham Minocycline | CCI Minocycline | Sham Vehicle | CCI Unaffected | CCI Affected | Sham Minocycline | CCI Minocycline |
| Hipp | Dorsal | DG | 18314 ± 2774  (*n*=6) | 18381 ± 1698  (*n*=6) | 19673 ± 4877  (*n*=6) | 16521 ± 2064  (*n*=6) | 18914 ± 4118  (*n*=6) | 19562 ± 3214  (*n*=6) | 18054 ± 4615  (*n*=6) | 20533 ± 3365  (*n*=6) | 18747 ± 4439  (*n*=6) | 18362 ± 2791  (*n*=6) |
|  |  | CA3 | 23130 ± 2835  (*n*=6) | 20299 ± 4499  (*n*=6) | 20390 ± 4287  (*n*=6) | 20064 ± 3475  (*n*=6) | 22000 ± 3255  (*n*=5) | 22079 ± 3125  (*n*=6) | 19406 ± 3707  (*n*=6) | 20747 ± 4304  (*n*=6) | 20841 ± 3630  (*n*=6) | 16348 ± 3475  (*n*=6) |
|  |  | CA1 | 17904 ± 1053  (*n*=6) | 20301 ± 3744  (*n*=6) | 19156 ± 5396  (*n*=6) | 18603 ± 3250  (*n*=6) | 19074 ± 897.3  (*n*=5) | 18789 ± 1925  (*n*=6) | 18472 ± 4336  (*n*=6) | 20816 ± 4087  (*n*=6) | 17806 ± 1568  (*n*=6) | 18677 ± 4536  (*n*=6) |
|  | Intermediate | DG | 20248 ± 3581  (*n*=6) | 18557 ± 3053  (*n*=6) | 21427 ± 4190  (*n*=6) | 18077 ± 5072  (*n*=6) | 21086 ± 4442  (*n*=6) | 20339 ± 3468  (*n*=6) | 20557 ± 3281  (*n*=6) | 23101 ± 5351  (*n*=6) | 17728 ± 4749  (*n*=6) | 17240 ± 1395  (*n*=6) |
|  |  | CA3 | 19376 ± 3427  (*n*=6) | 17731 ± 1672  (*n*=6) | 20261 ± 3364  (*n*=6) | 17549 ± 3301  (*n*=6) | 18853 ± 3672  (*n*=6) | 20547 ± 5705  (*n*=6) | 18408 ± 3539  (*n*=6) | 19697 ± 2400  (*n*=6) | 18109 ± 7098  (*n*=6) | 16083 ± 2563  (*n*=6) |
|  |  | CA1 | 21688 ± 1998  (*n*=6) | 17684 ± 816.9^$^  (*n*=6) | 22069 ± 3265^&^  (*n*=6) | 17688 ± 2281^+^  (*n*=6) | 19025 ± 2881  (*n*=6) | 19280 ± 3574  (*n*=6) | 20217 ± 2591  (*n*=6) | 21387 ± 4562  (*n*=6) | 19574 ± 4203  (*n*=6) | 21837 ± 3855  (*n*=6) |
|  | Ventral | DG | 22738 ± 7193  (*n*=6) | 19665 ± 3388  (*n*=6) | 21982 ± 3545  (*n*=6) | 18192 ± 2519  (*n*=5) | 20995 ± 5218  (*n*=5) | 21109 ± 4041  (*n*=6) | 19194 ± 3951  (*n*=6) | 19348 ± 6772  (*n*=6) | 21775 ± 3402  (*n*=6) | 21500 ± 6807  (*n*=5) |
|  |  | CA3 | 19221 ± 2612  (*n*=6) | 17410 ± 4904  (*n*=6) | 19707 ± 2158  (*n*=6) | 19122 ± 3156  (*n*=5) | 19872 ± 4670  (*n*=5) | 21572 ± 4734  (*n*=6) | 19893 ± 3396  (*n*=6) | 19279 ± 1298  (*n*=5) | 20704 ± 4959  (*n*=6) | 22374 ± 9839  (*n*=5) |
|  |  | CA1 | 19327 ± 4901  (*n*=6) | 17111 ± 2454  (*n*=6) | 19738 ± 3361  (*n*=6) | 17266 ± 2244  (*n*=5) | 17531 ± 5577  (*n*=5) | 18331 ± 3930  (*n*=6) | 17949 ± 4753  (*n*=6) | 20205 ± 3152  (*n*=5) | 18059 ± 3769  (*n*=6) | 21616 ± 2022  (*n*=5) |
|  | Ventral Pole | CA3 | 19406 ± 3585  (*n*=6) | 20133 ± 3402  (*n*=6) | 23311 ± 4084  (*n*=6) | 18632 ± 2161  (*n*=6) | 18327 ± 3724  (*n*=6) | 22044 ± 4088  (*n*=5) | 17868 ± 3962  (*n*=6) | 18283 ± 6503  (*n*=6) | 15586 ± 5894  (*n*=5) | 16397 ± 2590  (*n*=6) |
|  |  | CA1 | 25596 ± 6342  (*n*=6) | 21807 ± 4727  (*n*=6) | 20512 ± 3506  (*n*=6) | 20695 ± 3097  (*n*=6) | 19362 ± 3259  (*n*=6) | 20441 ± 3087  (*n*=5) | 22024 ± 5072  (*n*=6) | 21060 ± 3390  (*n*=6) | 17155 ± 2505  (*n*=4) | 19999 ± 1576  (*n*=6) |
| mPFC | Rostral | CG | 2215.6 ± 454.0  (*n*=6) | 1923.1 ± 238.5  (*n*=6) | 2802.9 ± 409.0^&&^  (*n*=5) | 2159.3 ± 354.9  (*n*=6) | 2328.3 ± 369.2  (*n*=6) | 2355.9 ± 343.0  (*n*=6) | 2391.5 ± 495.4  (*n*=6) | 2718.8 ± 611.7  (*n*=6) | 2276.8 ± 399.0  (*n*=6) | 2712.9 ± 822.7  (*n*=6) |
|  |  | PL | 2338.9 ± 191.8  (*n*=6) | 1908.5 ± 325.6  (*n*=6) | 2492.0 ± 477.1  (*n*=5) | 1949.2 ± 373.4  (*n*=6) | 2399.9 ± 450.9  (*n*=6) | 2330.3 ± 623.4  (*n*=6) | 2100.6 ± 671.4  (*n*=6) | 2551.9 ± 749.3  (*n*=6) | 1935.1 ± 170.9  (*n*=6) | 2197.4 ± 349.8  (*n*=6) |
|  |  | IL | 2315.0 ± 133.6  (*n*=5) | 2063.9 ± 251.3  (*n*=6) | 2375.4 ± 431.2  (*n*=6) | 2200.1 ± 660.7  (*n*=6) | 2213.5 ± 532.3  (*n*=6) | 2507.6 ± 475.3  (*n*=6) | 2057.1 ± 499.4  (*n*=6) | 2678.2 ± 798.1  (*n*=6) | 1818.1 ± 293.8  (*n*=6) | 2356.0 ± 530.3  (*n*=6) |
|  | Mid | CG | 2506.0 ± 349.9  (*n*=6) | 1785.3 ± 211.9^$/@^  (*n*=6) | 2375.8 ± 704.5  (*n*=6) | 2138.5 ± 305.2  (*n*=6) | 2586.2 ± 288.1  (*n*=5) | 2695.4 ± 269.7  (*n*=6) | 2441.8 ± 454.6  (*n*=6) | 2653.5 ± 503.1  (*n*=6) | 2315.9 ± 364.4  (*n*=6) | 2657.1 ± 519.0  (*n*=6) |
|  |  | PL | 2559.9 ± 655.8  (*n*=6) | 2236.2 ± 481.9  (*n*=6) | 2429.7 ± 598.9  (*n*=6) | 2137.4 ± 336.1  (*n*=6) | 2206.6 ± 347.6  (*n*=6) | 2307.5 ± 516.2  (*n*=6) | 1905.1 ± 296.7  (*n*=6) | 2375.0 ± 842.8  (*n*=6) | 2014.1 ± 174.1  (*n*=6) | 2232.4 ± 405.4  (*n*=6) |
|  |  | IL | 2584.1 ± 539.1  (*n*=6) | 2263.5 ± 272.1  (*n*=6) | 2507.4 ± 460.1  (*n*=6) | 1920.2 ± 424.9  (*n*=6) | 2202.6 ± 782.5  (*n*=6) | 2527.2 ± 795.2  (*n*=6) | 2092.3 ± 317.6  (*n*=6) | 2587.8 ± 816.1  (*n*=6) | 1967.9 ± 208.4  (*n*=6) | 2526.6 ± 664.4  (*n*=6) |
|  | Caudal | CG | 2298.2 ± 413.3  (*n*=6) | 2000.4 ± 428.3  (*n*=6) | 2470.9 ± 377.1  (*n*=6) | 1882.1 ± 405.8  (*n*=6) | 1861.5 ± 232.3  (*n*=6) | 2516.2 ± 537.0  (*n*=6) | 2076.5 ± 458.3  (*n*=6) | 2387.0 ± 268.8  (*n*=6) | 1807.3 ± 277.5^+^  (*n*=6) | 2254.9 ± 381.1  (*n*=6) |
|  |  | PL | 2304.3 ± 431.2  (*n*=6) | 1744.6 ± 261.3^$^  (*n*=6) | 2121.2 ± 300.4  (*n*=6) | 1811.9 ± 273.7  (*n*=6) | 1723.6 ± 276.4  (*n*=6) | 2388.1 ± 465.4  (*n*=6) | 2288.9 ± 519.3  (*n*=6) | 2375.0 ± 549.1  (*n*=6) | 1627.7 ± 196.1^+^  (*n*=6) | 1777.7 ± 342.9  (*n*=6) |
|  |  | IL | 2686.2 ± 605.8  (*n*=6) | 2265.5 ± 525.8  (*n*=6) | 2442.6 ± 426.4  (*n*=6) | 1739.4 ± 330.4  (*n*=6) | 2191.8 ± 970.2  (*n*=6) | 2617.2 ± 644.0  (*n*=6) | 2527.3 ± 660.6  (*n*=6) | 1959.0 ± 368.4  (*n*=6) | 1959.2 ± 405.3  (*n*=6) | 2070.9 ± 678.9  (*n*=6) |

**Supplementary Table C2**. Circularity values from fractal morphological analysis of IBA1+ microglia. Values represent group means ± standard deviation (SD). The mean value from all microglia measured within the field of view was used to calculate the individual value for each animal. These values were then used to compute means and SD within experimental groups (i.e. *n* refers to biological, rather than technical, replicates). Results where *n* < 3 are excluded. Hipp: hippocampus; mPFC: medial prefrontal cortex; VPL: ventroposterior lateral nucleus; DG: dentate gyrus; CG: cingulate gyrus; PL: prelimbic cortex; IL: infralimbic cortex; DL: dorsolateral; VM: ventromedial; ZI: zona incerta. *: Sham vehicle vs CCI affected; $: Sham vehicle vs CCI unaffected; ^: Sham minocycline vs CCI minocycline; @: CCI unaffected vs CCI minocycline; #: CCI affected vs CCI minocycline; &: CCI unaffected vs CCI affected; +: Sham vehicle vs Sham minocycline. **P_adj_* < 0.05*; ** P_adj_* < 0.01.

|  | | | Left (Contralateral) | | | | | Right (Ipsilateral) | | | | |
| --- | --- | --- | --- | --- | --- | --- | --- | --- | --- | --- | --- | --- |
| Region | AP Level | Subregion | Sham Vehicle | CCI Unaffected | CCI Affected | Sham Minocycline | CCI Minocycline | Sham Vehicle | CCI Unaffected | CCI Affected | Sham Minocycline | CCI Minocycline |
| Hipp | Dorsal | DG | 0.763 ± 0.020  (*n*=6) | 0.766 ± 0.016  (*n*=6) | 0.776 ± 0.012  (*n*=6) | 0.769 ± 0.009  (*n*=6) | 0.776 ± 0.017  (*n*=6) | 0.773 ± 0.019  (*n*=6) | 0.768 ± 0.017  (*n*=6) | 0.783 ± 0.010  (*n*=6) | 0.770 ± 0.010  (*n*=6) | 0.770 ± 0.017  (*n*=6) |
|  |  | CA3 | 0.752 ± 0.014  (*n*=6) | 0.761 ± 0.015  (*n*=6) | 0.755 ± 0.017  (*n*=6) | 0.762 ± 0.022  (*n*=6) | 0.763 ± 0.017  (*n*=5) | 0.771 ± 0.007  (*n*=6) | 0.754 ± 0.021  (*n*=6) | 0.762 ± 0.013  (*n*=6) | 0.761 ± 0.023  (*n*=6) | 0.748 ± 0.018  (*n*=6) |
|  |  | CA1 | 0.776 ± 0.013  (*n*=6) | 0.777 ± 0.011  (*n*=6) | 0.759 ± 0.021  (*n*=6) | 0.773 ± 0.017  (*n*=6) | 0.767 ± 0.011  (*n*=5) | 0.761 ± 0.023  (*n*=6) | 0.758 ± 0.015  (*n*=6) | 0.774 ± 0.014  (*n*=6) | 0.760 ± 0.017  (*n*=6) | 0.777 ± 0.013  (*n*=6) |
|  | Intermediate | DG | 0.770 ± 0.016  (*n*=6) | 0.762 ± 0.011  (*n*=6) | 0.770 ± 0.016  (*n*=6) | 0.766 ± 0.022  (*n*=6) | 0.780 ± 0.012  (*n*=6) | 0.772 ± 0.007  (*n*=6) | 0.773 ± 0.013  (*n*=6) | 0.781 ± 0.009  (*n*=6) | 0.754 ± 0.031  (*n*=6) | 0.758 ± 0.015  (*n*=6) |
|  |  | CA3 | 0.770 ± 0.015  (*n*=6) | 0.759 ± 0.017  (*n*=6) | 0.775 ± 0.013  (*n*=6) | 0.760 ± 0.023  (*n*=6) | 0.764 ± 0.018  (*n*=6) | 0.766 ± 0.019  (*n*=6) | 0.763 ± 0.018  (*n*=6) | 0.762 ± 0.010  (*n*=6) | 0.759 ± 0.023  (*n*=6) | 0.768 ± 0.019  (*n*=6) |
|  |  | CA1 | 0.769 ± 0.010  (*n*=6) | 0.759 ± 0.017  (*n*=6) | 0.779 ± 0.017  (*n*=6) | 0.759 ± 0.022  (*n*=6) | 0.755 ± 0.023  (*n*=6) | 0.764 ± 0.024  (*n*=6) | 0.767 ± 0.015  (*n*=6) | 0.762 ± 0.032  (*n*=6) | 0.758 ± 0.019  (*n*=6) | 0.780 ± 0.018  (*n*=6) |
|  | Ventral | DG | 0.772 ± 0.015  (*n*=6) | 0.773 ± 0.014  (*n*=6) | 0.791 ± 0.008  (*n*=6) | 0.772 ± 0.009  (*n*=5) | 0.775 ± 0.015  (*n*=5) | 0.777 ± 0.011  (*n*=6) | 0.776 ± 0.011  (*n*=6) | 0.771 ± 0.022  (*n*=6) | 0.780 ± 0.010  (*n*=6) | 0.776 ± 0.031  (*n*=5) |
|  |  | CA3 | 0.767 ± 0.015  (*n*=6) | 0.762 ± 0.015  (*n*=6) | 0.767 ± 0.010  (*n*=6) | 0.771 ± 0.020  (*n*=5) | 0.766 ± 0.022  (*n*=5) | 0.767 ± 0.013  (*n*=6) | 0.768 ± 0.016  (*n*=6) | 0.769 ± 0.018  (*n*=5) | 0.775 ± 0.004  (*n*=6) | 0.776 ± 0.024  (*n*=5) |
|  |  | CA1 | 0.759 ± 0.016  (*n*=6) | 0.767 ± 0.017  (*n*=6) | 0.772 ± 0.015  (*n*=6) | 0.758 ± 0.024  (*n*=5) | 0.759 ± 0.011  (*n*=5) | 0.755 ± 0.010  (*n*=6) | 0.769 ± 0.012  (*n*=6) | 0.781 ± 0.009**  (*n*=5) | 0.762 ± 0.011  (*n*=6) | 0.765 ± 0.016  (*n*=5) |
|  | Ventral Pole | CA3 | 0.773 ± 0.013  (*n*=6) | 0.772 ± 0.007  (*n*=6) | 0.781 ± 0.011  (*n*=6) | 0.775 ± 0.015  (*n*=6) | 0.775 ± 0.013  (*n*=6) | 0.771 ± 0.014  (*n*=5) | 0.762 ± 0.028  (*n*=6) | 0.767 ± 0.014  (*n*=6) | 0.753 ± 0.038  (*n*=5) | 0.773 ± 0.010  (*n*=6) |
|  |  | CA1 | 0.778 ± 0.012  (*n*=6) | 0.775 ± 0.021  (*n*=6) | 0.773 ± 0.016  (*n*=6) | 0.772 ± 0.018  (*n*=6) | 0.782 ± 0.011  (*n*=6) | 0.776 ± 0.019  (*n*=5) | 0.774 ± 0.016  (*n*=6) | 0.773 ± 0.010  (*n*=6) | 0.759 ± 0.010  (*n*=4) | 0.771 ± 0.013  (*n*=6) |
| mPFC | Rostral | CG | 0.755 ± 0.010  (*n*=6) | 0.756 ± 0.024  (*n*=6) | 0.758 ± 0.006  (*n*=5) | 0.748 ± 0.012  (*n*=6) | 0.753 ± 0.012  (*n*=6) | 0.752 ± 0.020  (*n*=6) | 0.757 ± 0.006  (*n*=6) | 0.752 ± 0.007  (*n*=6) | 0.757 ± 0.017  (*n*=6) | 0.759 ± 0.010  (*n*=6) |
|  |  | PL | 0.763 ± 0.006  (*n*=6) | 0.758 ± 0.023  (*n*=6) | 0.758 ± 0.016  (*n*=5) | 0.748 ± 0.011  (*n*=6) | 0.761 ± 0.015  (*n*=6) | 0.760 ± 0.010  (*n*=6) | 0.759 ± 0.023  (*n*=6) | 0.759 ± 0.019  (*n*=6) | 0.761 ± 0.011  (*n*=6) | 0.752 ± 0.015  (*n*=6) |
|  |  | IL | 0.765 ± 0.011  (*n*=5) | 0.761 ± 0.012  (*n*=6) | 0.761 ± 0.020  (*n*=6) | 0.760 ± 0.012  (*n*=6) | 0.758 ± 0.015  (*n*=6) | 0.766 ± 0.019  (*n*=6) | 0.769 ± 0.017  (*n*=6) | 0.773 ± 0.014  (*n*=6) | 0.759 ± 0.013  (*n*=6) | 0.768 ± 0.016  (*n*=6) |
|  | Mid | CG | 0.759 ± 0.012  (*n*=6) | 0.742 ± 0.014  (*n*=6) | 0.753 ± 0.024  (*n*=6) | 0.744 ± 0.009  (*n*=6) | 0.752 ± 0.013  (*n*=5) | 0.765 ± 0.008  (*n*=6) | 0.750 ± 0.018  (*n*=6) | 0.755 ± 0.019  (*n*=6) | 0.750 ± 0.010  (*n*=6) | 0.752 ± 0.020  (*n*=6) |
|  |  | PL | 0.766 ± 0.022  (*n*=6) | 0.749 ± 0.022  (*n*=6) | 0.749 ± 0.021  (*n*=6) | 0.753 ± 0.015  (*n*=6) | 0.753 ± 0.014  (*n*=6) | 0.758 ± 0.016  (*n*=6) | 0.747 ± 0.022  (*n*=6) | 0.756 ± 0.028  (*n*=6) | 0.742 ± 0.015  (*n*=6) | 0.756 ± 0.006  (*n*=6) |
|  |  | IL | 0.763 ± 0.019  (*n*=6) | 0.765 ± 0.014  (*n*=6) | 0.764 ± 0.017  (*n*=6) | 0.753 ± 0.014  (*n*=6) | 0.754 ± 0.027  (*n*=6) | 0.769 ± 0.020  (*n*=6) | 0.764 ± 0.016  (*n*=6) | 0.772 ± 0.019  (*n*=6) | 0.749 ± 0.011  (*n*=6) | 0.765 ± 0.016  (*n*=6) |
|  | Caudal | CG | 0.754 ± 0.020  (*n*=6) | 0.736 ± 0.024  (*n*=6) | 0.749 ± 0.021  (*n*=6) | 0.744 ± 0.014  (*n*=6) | 0.735 ± 0.017  (*n*=6) | 0.757 ± 0.015  (*n*=6) | 0.747 ± 0.022  (*n*=6) | 0.745 ± 0.022  (*n*=6) | 0.740 ± 0.006  (*n*=6) | 0.751 ± 0.012  (*n*=6) |
|  |  | PL | 0.764 ± 0.011  (*n*=6) | 0.739 ± 0.017  (*n*=6) | 0.744 ± 0.006  (*n*=6) | 0.740 ± 0.013  (*n*=6) | 0.743 ± 0.024  (*n*=6) | 0.755 ± 0.013  (*n*=6) | 0.755 ± 0.017  (*n*=6) | 0.756 ± 0.010  (*n*=6) | 0.736 ± 0.017  (*n*=6) | 0.743 ± 0.013  (*n*=6) |
|  |  | IL | 0.768 ± 0.011  (*n*=6) | 0.763 ± 0.012  (*n*=6) | 0.768 ± 0.005  (*n*=6) | 0.754 ± 0.005  (*n*=6) | 0.760 ± 0.017  (*n*=6) | 0.770 ± 0.013  (*n*=6) | 0.761 ± 0.011  (*n*=6) | 0.756 ± 0.013  (*n*=6) | 0.755 ± 0.010  (*n*=6) | 0.750 ± 0.020  (*n*=6) |

**Supplementary Table C3**. Density values from fractal morphological analysis of IBA1+ microglia. Values represent group means ± standard deviation (SD). The mean value from all microglia measured within the field of view was used to calculate the individual value for each animal. These values were then used to compute means and SD within experimental groups (i.e. *n* refers to biological, rather than technical, replicates). Results where *n* < 3 are excluded. Hipp: hippocampus; mPFC: medial prefrontal cortex; VPL: ventroposterior lateral nucleus; DG: dentate gyrus; CG: cingulate gyrus; PL: prelimbic cortex; IL: infralimbic cortex; DL: dorsolateral; VM: ventromedial; ZI: zona incerta. *: Sham vehicle vs CCI affected; $: Sham vehicle vs CCI unaffected; ^: Sham minocycline vs CCI minocycline; @: CCI unaffected vs CCI minocycline; #: CCI affected vs CCI minocycline; &: CCI unaffected vs CCI affected; +: Sham vehicle vs Sham minocycline. **P_adj_* < 0.05*; ** P_adj_* < 0.01.

|  | | | Left (Contralateral) | | | | | Right (Ipsilateral) | | | | |
| --- | --- | --- | --- | --- | --- | --- | --- | --- | --- | --- | --- | --- |
| Region | AP Level | Subregion | Sham Vehicle | CCI Unaffected | CCI Affected | Sham Minocycline | CCI Minocycline | Sham Vehicle | CCI Unaffected | CCI Affected | Sham Minocycline | CCI Minocycline |
| Hipp | Dorsal | DG | 0.281 ± 0.022  (*n*=6) | 0.269 ± 0.014  (*n*=6) | 0.286 ± 0.022  (*n*=6) | 0.292 ± 0.024  (*n*=6) | 0.269 ± 0.027  (*n*=6) | 0.282 ± 0.026  (*n*=6) | 0.271 ± 0.010  (*n*=6) | 0.272 ± 0.010  (*n*=6) | 0.287 ± 0.014  (*n*=6) | 0.269 ± 0.027  (*n*=6) |
|  |  | CA3 | 0.257 ± 0.019  (*n*=6) | 0.249 ± 0.015  (*n*=6) | 0.275 ± 0.018  (*n*=6) | 0.248 ± 0.019  (*n*=6) | 0.267 ± 0.049  (*n*=5) | 0.255 ± 0.017  (*n*=6) | 0.250 ± 0.008  (*n*=6) | 0.262 ± 0.018  (*n*=6) | 0.248 ± 0.022  (*n*=6) | 0.269 ± 0.016  (*n*=6) |
|  |  | CA1 | 0.267 ± 0.012  (*n*=6) | 0.262 ± 0.011  (*n*=6) | 0.274 ± 0.022  (*n*=6) | 0.255 ± 0.012  (*n*=6) | 0.273 ± 0.024  (*n*=5) | 0.275 ± 0.018  (*n*=6) | 0.271 ± 0.022  (*n*=6) | 0.260 ± 0.018  (*n*=6) | 0.262 ± 0.017  (*n*=6) | 0.267 ± 0.025  (*n*=6) |
|  | Intermediate | DG | 0.286 ± 0.028  (*n*=6) | 0.280 ± 0.019  (*n*=6) | 0.272 ± 0.022  (*n*=6) | 0.278 ± 0.026  (*n*=6) | 0.279 ± 0.025  (*n*=6) | 0.280 ± 0.018  (*n*=6) | 0.273 ± 0.008  (*n*=6) | 0.272 ± 0.025  (*n*=6) | 0.277 ± 0.017  (*n*=6) | 0.273 ± 0.012  (*n*=6) |
|  |  | CA3 | 0.263 ± 0.019  (*n*=6) | 0.259 ± 0.010  (*n*=6) | 0.260 ± 0.010  (*n*=6) | 0.260 ± 0.015  (*n*=6) | 0.271 ± 0.019  (*n*=6) | 0.262 ± 0.028  (*n*=6) | 0.271 ± 0.022  (*n*=6) | 0.264 ± 0.013  (*n*=6) | 0.295 ± 0.056  (*n*=6) | 0.273 ± 0.018  (*n*=6) |
|  |  | CA1 | 0.268 ± 0.024  (*n*=6) | 0.269 ± 0.023  (*n*=6) | 0.268 ± 0.016  (*n*=6) | 0.262 ± 0.015  (*n*=6) | 0.256 ± 0.015  (*n*=6) | 0.266 ± 0.031  (*n*=6) | 0.257 ± 0.014  (*n*=6) | 0.268 ± 0.017  (*n*=6) | 0.263 ± 0.021  (*n*=6) | 0.252 ± 0.017  (*n*=6) |
|  | Ventral | DG | 0.290 ± 0.033  (*n*=6) | 0.302 ± 0.022  (*n*=6) | 0.291 ± 0.018  (*n*=6) | 0.294 ± 0.006  (*n*=5) | 0.274 ± 0.011  (*n*=5) | 0.283 ± 0.018  (*n*=6) | 0.295 ± 0.031  (*n*=6) | 0.319 ± 0.058  (*n*=6) | 0.278 ± 0.016  (*n*=6) | 0.276 ± 0.017  (*n*=5) |
|  |  | CA3 | 0.268 ± 0.022  (*n*=6) | 0.280 ± 0.020  (*n*=6) | 0.272 ± 0.019  (*n*=6) | 0.268 ± 0.009  (*n*=5) | 0.266 ± 0.016  (*n*=5) | 0.273 ± 0.023  (*n*=6) | 0.274 ± 0.003  (*n*=6) | 0.261 ± 0.012  (*n*=5) | 0.260 ± 0.018  (*n*=6) | 0.267 ± 0.029  (*n*=5) |
|  |  | CA1 | 0.266 ± 0.016  (*n*=6) | 0.274 ± 0.021  (*n*=6) | 0.274 ± 0.019  (*n*=6) | 0.269 ± 0.026  (*n*=5) | 0.285 ± 0.040  (*n*=5) | 0.278 ± 0.017  (*n*=6) | 0.275 ± 0.015  (*n*=6) | 0.259 ± 0.016  (*n*=5) | 0.275 ± 0.017  (*n*=6) | 0.268 ± 0.014  (*n*=5) |
|  | Ventral Pole | CA3 | 0.288 ± 0.033  (*n*=6) | 0.264 ± 0.026  (*n*=6) | 0.269 ± 0.015  (*n*=6) | 0.277 ± 0.011  (*n*=6) | 0.273 ± 0.018  (*n*=6) | 0.273 ± 0.028  (*n*=5) | 0.270 ± 0.024  (*n*=6) | 0.306 ± 0.052  (*n*=6) | 0.282 ± 0.034  (*n*=5) | 0.280 ± 0.021  (*n*=6) |
|  |  | CA1 | 0.251 ± 0.018  (*n*=6) | 0.273 ± 0.026  (*n*=6) | 0.269 ± 0.008  (*n*=6) | 0.263 ± 0.009  (*n*=6) | 0.273 ± 0.018  (*n*=6) | 0.281 ± 0.024  (*n*=5) | 0.268 ± 0.022  (*n*=6) | 0.275 ± 0.013  (*n*=6) | 0.271 ± 0.018  (*n*=4) | 0.274 ± 0.013  (*n*=6) |
| mPFC | Rostral | CG | 0.411 ± 0.019  (*n*=6) | 0.432 ± 0.023  (*n*=6) | 0.390 ± 0.015  (*n*=5) | 0.416 ± 0.015  (*n*=6) | 0.412 ± 0.017  (*n*=6) | 0.410 ± 0.020  (*n*=6) | 0.403 ± 0.024  (*n*=6) | 0.400 ± 0.029  (*n*=6) | 0.417 ± 0.016  (*n*=6) | 0.392 ± 0.020  (*n*=6) |
|  |  | PL | 0.411 ± 0.017  (*n*=6) | 0.408 ± 0.019  (*n*=6) | 0.392 ± 0.020  (*n*=5) | 0.408 ± 0.022  (*n*=6) | 0.401 ± 0.017  (*n*=6) | 0.414 ± 0.020  (*n*=6) | 0.411 ± 0.026  (*n*=6) | 0.410 ± 0.024  (*n*=6) | 0.411 ± 0.021  (*n*=6) | 0.402 ± 0.010  (*n*=6) |
|  |  | IL | 0.413 ± 0.008  (*n*=5) | 0.409 ± 0.019  (*n*=6) | 0.406 ± 0.015  (*n*=6) | 0.397 ± 0.029  (*n*=6) | 0.413 ± 0.027  (*n*=6) | 0.401 ± 0.012  (*n*=6) | 0.412 ± 0.029  (*n*=6) | 0.406 ± 0.021  (*n*=6) | 0.416 ± 0.020  (*n*=6) | 0.406 ± 0.024  (*n*=6) |
|  | Mid | CG | 0.397 ± 0.016  (*n*=6) | 0.417 ± 0.013  (*n*=6) | 0.391 ± 0.025  (*n*=6) | 0.408 ± 0.014  (*n*=6) | 0.403 ± 0.022  (*n*=5) | 0.392 ± 0.013  (*n*=6) | 0.412 ± 0.016  (*n*=6) | 0.396 ± 0.022  (*n*=6) | 0.411 ± 0.016  (*n*=6) | 0.393 ± 0.023  (*n*=6) |
|  |  | PL | 0.404 ± 0.011  (*n*=6) | 0.394 ± 0.032  (*n*=6) | 0.386 ± 0.019  (*n*=6) | 0.395 ± 0.032  (*n*=6) | 0.392 ± 0.015  (*n*=6) | 0.405 ± 0.016  (*n*=6) | 0.418 ± 0.032  (*n*=6) | 0.401 ± 0.014  (*n*=6) | 0.399 ± 0.025  (*n*=6) | 0.401 ± 0.023  (*n*=6) |
|  |  | IL | 0.403 ± 0.014  (*n*=6) | 0.391 ± 0.016  (*n*=6) | 0.376 ± 0.006  (*n*=6) | 0.394 ± 0.017  (*n*=6) | 0.410 ± 0.022  (*n*=6) | 0.413 ± 0.027  (*n*=6) | 0.413 ± 0.038  (*n*=6) | 0.398 ± 0.018  (*n*=6) | 0.391 ± 0.019  (*n*=6) | 0.392 ± 0.028  (*n*=6) |
|  | Caudal | CG | 0.409 ± 0.019  (*n*=6) | 0.412 ± 0.015  (*n*=6) | 0.407 ± 0.022  (*n*=6) | 0.408 ± 0.016  (*n*=6) | 0.413 ± 0.009  (*n*=6) | 0.394 ± 0.008  (*n*=6) | 0.402 ± 0.016  (*n*=6) | 0.385 ± 0.014  (*n*=6) | 0.410 ± 0.012  (*n*=6) | 0.410 ± 0.020  (*n*=6) |
|  |  | PL | 0.405 ± 0.022  (*n*=6) | 0.409 ± 0.014  (*n*=6) | 0.395 ± 0.011  (*n*=6) | 0.407 ± 0.017  (*n*=6) | 0.415 ± 0.025  (*n*=6) | 0.389 ± 0.013  (*n*=6) | 0.409 ± 0.033  (*n*=6) | 0.389 ± 0.026  (*n*=6) | 0.421 ± 0.016  (*n*=6) | 0.418 ± 0.023  (*n*=6) |
|  |  | IL | 0.388 ± 0.021  (*n*=6) | 0.413 ± 0.027  (*n*=6) | 0.381 ± 0.026  (*n*=6) | 0.409 ± 0.014  (*n*=6) | 0.411 ± 0.016  (*n*=6) | 0.402 ± 0.015  (*n*=6) | 0.398 ± 0.017  (*n*=6) | 0.397 ± 0.021  (*n*=6) | 0.407 ± 0.010  (*n*=6) | 0.413 ± 0.027  (*n*=6) |

**Supplementary Table C4**. Fractal dimension values from fractal morphological analysis of IBA1+ microglia. Values represent group means ± standard deviation (SD). The mean value from all microglia measured within the field of view was used to calculate the individual value for each animal. These values were then used to compute means and SD within experimental groups (i.e. *n* refers to biological, rather than technical, replicates). Results where *n* < 3 are excluded. Hipp: hippocampus; mPFC: medial prefrontal cortex; VPL: ventroposterior lateral nucleus; DG: dentate gyrus; CG: cingulate gyrus; PL: prelimbic cortex; IL: infralimbic cortex; DL: dorsolateral; VM: ventromedial; ZI: zona incerta. *: Sham vehicle vs CCI affected; $: Sham vehicle vs CCI unaffected; ^: Sham minocycline vs CCI minocycline; @: CCI unaffected vs CCI minocycline; #: CCI affected vs CCI minocycline; &: CCI unaffected vs CCI affected; +: Sham vehicle vs Sham minocycline. **P_adj_* < 0.05*; ** P_adj_* < 0.01.

|  | | | Left (Contralateral) | | | | | Right (Ipsilateral) | | | | |
| --- | --- | --- | --- | --- | --- | --- | --- | --- | --- | --- | --- | --- |
| Region | AP Level | Subregion | Sham Vehicle | CCI Unaffected | CCI Affected | Sham Minocycline | CCI Minocycline | Sham Vehicle | CCI Unaffected | CCI Affected | Sham Minocycline | CCI Minocycline |
| Hipp | Dorsal | DG | 1.479 ± 0.017  (*n*=6) | 1.473 ± 0.012  (*n*=6) | 1.489 ± 0.021  (*n*=6) | 1.485 ± 0.019  (*n*=6) | 1.473 ± 0.016  (*n*=6) | 1.483 ± 0.020  (*n*=6) | 1.472 ± 0.014  (*n*=6) | 1.479 ± 0.003  (*n*=6) | 1.484 ± 0.016  (*n*=6) | 1.469 ± 0.021  (*n*=6) |
|  |  | CA3 | 1.468 ± 0.016  (*n*=6) | 1.454 ± 0.014  (*n*=6) | 1.474 ± 0.013  (*n*=6) | 1.451 ± 0.017  (*n*=6) | 1.469 ± 0.038  (*n*=5) | 1.464 ± 0.015  (*n*=6) | 1.456 ± 0.008  (*n*=6) | 1.466 ± 0.009  (*n*=6) | 1.453 ± 0.016  (*n*=6) | 1.461 ± 0.009  (*n*=6) |
|  |  | CA1 | 1.469 ± 0.011  (*n*=6) | 1.461 ± 0.019  (*n*=6) | 1.471 ± 0.014  (*n*=6) | 1.450 ± 0.006  (*n*=6) | 1.468 ± 0.021  (*n*=5) | 1.472 ± 0.015  (*n*=6) | 1.463 ± 0.011  (*n*=6) | 1.464 ± 0.012  (*n*=6) | 1.459 ± 0.014  (*n*=6) | 1.462 ± 0.012  (*n*=6) |
|  | Intermediate | DG | 1.491 ± 0.019  (*n*=6) | 1.479 ± 0.011  (*n*=6) | 1.476 ± 0.014  (*n*=6) | 1.477 ± 0.016  (*n*=6) | 1.481 ± 0.018  (*n*=6) | 1.483 ± 0.012  (*n*=6) | 1.478 ± 0.012  (*n*=6) | 1.482 ± 0.012  (*n*=6) | 1.472 ± 0.016  (*n*=6) | 1.471 ± 0.012  (*n*=6) |
|  |  | CA3 | 1.463 ± 0.018  (*n*=6) | 1.456 ± 0.014  (*n*=6) | 1.463 ± 0.010  (*n*=6) | 1.457 ± 0.013  (*n*=6) | 1.467 ± 0.016  (*n*=6) | 1.462 ± 0.011  (*n*=6) | 1.465 ± 0.019  (*n*=6) | 1.465 ± 0.011  (*n*=6) | 1.481 ± 0.024  (*n*=6) | 1.466 ± 0.008  (*n*=6) |
|  |  | CA1 | 1.475 ± 0.020  (*n*=6) | 1.465 ± 0.013  (*n*=6) | 1.475 ± 0.011  (*n*=6) | 1.459 ± 0.011  (*n*=6) | 1.456 ± 0.018  (*n*=6) | 1.469 ± 0.023  (*n*=6) | 1.461 ± 0.006  (*n*=6) | 1.470 ± 0.020  (*n*=6) | 1.466 ± 0.013  (*n*=6) | 1.459 ± 0.017  (*n*=6) |
|  | Ventral | DG | 1.493 ± 0.024  (*n*=6) | 1.497 ± 0.010  (*n*=6) | 1.498 ± 0.013  (*n*=6) | 1.491 ± 0.013  (*n*=5) | 1.480 ± 0.015  (*n*=5) | 1.488 ± 0.019  (*n*=6) | 1.493 ± 0.015  (*n*=6) | 1.509 ± 0.030  (*n*=6) | 1.485 ± 0.012  (*n*=6) | 1.484 ± 0.014  (*n*=5) |
|  |  | CA3 | 1.472 ± 0.015  (*n*=6) | 1.472 ± 0.014  (*n*=6) | 1.471 ± 0.016  (*n*=6) | 1.468 ± 0.009  (*n*=5) | 1.470 ± 0.013  (*n*=5) | 1.475 ± 0.019  (*n*=6) | 1.479 ± 0.010  (*n*=6) | 1.467 ± 0.007  (*n*=5) | 1.462 ± 0.016  (*n*=6) | 1.472 ± 0.024  (*n*=5) |
|  |  | CA1 | 1.466 ± 0.006  (*n*=6) | 1.469 ± 0.016  (*n*=6) | 1.474 ± 0.014  (*n*=6) | 1.466 ± 0.014  (*n*=5) | 1.475 ± 0.016  (*n*=5) | 1.475 ± 0.012  (*n*=6) | 1.474 ± 0.012  (*n*=6) | 1.466 ± 0.012  (*n*=5) | 1.470 ± 0.009  (*n*=6) | 1.474 ± 0.011  (*n*=5) |
|  | Ventral Pole | CA3 | 1.485 ± 0.022  (*n*=6) | 1.465 ± 0.018  (*n*=6) | 1.479 ± 0.011  (*n*=6) | 1.474 ± 0.009  (*n*=6) | 1.473 ± 0.015  (*n*=6) | 1.479 ± 0.032  (*n*=5) | 1.470 ± 0.023  (*n*=6) | 1.497 ± 0.031  (*n*=6) | 1.472 ± 0.017  (*n*=5) | 1.470 ± 0.012  (*n*=6) |
|  |  | CA1 | 1.470 ± 0.016  (*n*=6) | 1.477 ± 0.021  (*n*=6) | 1.473 ± 0.010  (*n*=6) | 1.467 ± 0.014  (*n*=6) | 1.473 ± 0.015  (*n*=6) | 1.486 ± 0.024  (*n*=5) | 1.475 ± 0.024  (*n*=6) | 1.480 ± 0.013  (*n*=6) | 1.470 ± 0.014  (*n*=4) | 1.476 ± 0.009  (*n*=6) |
| mPFC | Rostral | CG | 1.423 ± 0.019  (*n*=6) | 1.427 ± 0.014  (*n*=6) | 1.426 ± 0.007  (*n*=5) | 1.427 ± 0.010  (*n*=6) | 1.430 ± 0.011  (*n*=6) | 1.426 ± 0.015  (*n*=6) | 1.424 ± 0.005  (*n*=6) | 1.432 ± 0.032  (*n*=6) | 1.430 ± 0.008  (*n*=6) | 1.423 ± 0.013  (*n*=6) |
|  |  | PL | 1.434 ± 0.012  (*n*=6) | 1.418 ± 0.007  (*n*=6) | 1.416 ± 0.009  (*n*=5) | 1.415 ± 0.008^+^  (*n*=6) | 1.425 ± 0.012  (*n*=6) | 1.431 ± 0.021  (*n*=6) | 1.426 ± 0.021  (*n*=6) | 1.431 ± 0.034  (*n*=6) | 1.420 ± 0.013  (*n*=6) | 1.419 ± 0.017  (*n*=6) |
|  |  | IL | 1.434 ± 0.005  (*n*=5) | 1.423 ± 0.010  (*n*=6) | 1.429 ± 0.022  (*n*=6) | 1.417 ± 0.008  (*n*=6) | 1.427 ± 0.011  (*n*=6) | 1.431 ± 0.019  (*n*=6) | 1.425 ± 0.023  (*n*=6) | 1.440 ± 0.025  (*n*=6) | 1.420 ± 0.015  (*n*=6) | 1.427 ± 0.015  (*n*=6) |
|  | Mid | CG | 1.424 ± 0.009  (*n*=6) | 1.417 ± 0.006  (*n*=6) | 1.418 ± 0.015  (*n*=6) | 1.416 ± 0.016  (*n*=6) | 1.430 ± 0.018  (*n*=5) | 1.427 ± 0.014  (*n*=6) | 1.430 ± 0.013  (*n*=6) | 1.426 ± 0.024  (*n*=6) | 1.422 ± 0.010  (*n*=6) | 1.424 ± 0.019  (*n*=6) |
|  |  | PL | 1.430 ± 0.026  (*n*=6) | 1.411 ± 0.012  (*n*=6) | 1.414 ± 0.020  (*n*=6) | 1.413 ± 0.021  (*n*=6) | 1.411 ± 0.016  (*n*=6) | 1.423 ± 0.021  (*n*=6) | 1.417 ± 0.020  (*n*=6) | 1.424 ± 0.029  (*n*=6) | 1.410 ± 0.016  (*n*=6) | 1.419 ± 0.017  (*n*=6) |
|  |  | IL | 1.431 ± 0.019  (*n*=6) | 1.414 ± 0.008  (*n*=6) | 1.408 ± 0.013*  (*n*=6) | 1.405 ± 0.011^+^  (*n*=6) | 1.425 ± 0.011^^^  (*n*=6) | 1.435 ± 0.034  (*n*=6) | 1.424 ± 0.030  (*n*=6) | 1.431 ± 0.024  (*n*=6) | 1.404 ± 0.013  (*n*=6) | 1.423 ± 0.029  (*n*=6) |
|  | Caudal | CG | 1.429 ± 0.009  (*n*=6) | 1.422 ± 0.017  (*n*=6) | 1.429 ± 0.020  (*n*=6) | 1.413 ± 0.009  (*n*=6) | 1.410 ± 0.011  (*n*=6) | 1.424 ± 0.013  (*n*=6) | 1.411 ± 0.013  (*n*=6) | 1.411 ± 0.008  (*n*=6) | 1.412 ± 0.006  (*n*=6) | 1.425 ± 0.017  (*n*=6) |
|  |  | PL | 1.426 ± 0.011  (*n*=6) | 1.411 ± 0.013  (*n*=6) | 1.412 ± 0.014  (*n*=6) | 1.411 ± 0.010  (*n*=6) | 1.409 ± 0.017  (*n*=6) | 1.416 ± 0.012  (*n*=6) | 1.427 ± 0.022  (*n*=6) | 1.412 ± 0.029  (*n*=6) | 1.413 ± 0.008  (*n*=6) | 1.417 ± 0.008  (*n*=6) |
|  |  | IL | 1.426 ± 0.019  (*n*=6) | 1.429 ± 0.028  (*n*=6) | 1.414 ± 0.029  (*n*=6) | 1.408 ± 0.012  (*n*=6) | 1.422 ± 0.031  (*n*=6) | 1.429 ± 0.026  (*n*=6) | 1.426 ± 0.021  (*n*=6) | 1.409 ± 0.014  (*n*=6) | 1.416 ± 0.011  (*n*=6) | 1.423 ± 0.030  (*n*=6) |

**Supplementary Table C5**. Lacunarity values from fractal morphological analysis of IBA1+ microglia. Values represent group means ± standard deviation (SD). The mean value from all microglia measured within the field of view was used to calculate the individual value for each animal. These values were then used to compute means and SD within experimental groups (i.e. *n* refers to biological, rather than technical, replicates). Results where *n* < 3 are excluded. Hipp: hippocampus; mPFC: medial prefrontal cortex; VPL: ventroposterior lateral nucleus; DG: dentate gyrus; CG: cingulate gyrus; PL: prelimbic cortex; IL: infralimbic cortex; DL: dorsolateral; VM: ventromedial; ZI: zona incerta. *: Sham vehicle vs CCI affected; $: Sham vehicle vs CCI unaffected; ^: Sham minocycline vs CCI minocycline; @: CCI unaffected vs CCI minocycline; #: CCI affected vs CCI minocycline; &: CCI unaffected vs CCI affected; +: Sham vehicle vs Sham minocycline. **P_adj_* < 0.05*; ** P_adj_* < 0.01.

|  | | | Left (Contralateral) | | | | | Right (Ipsilateral) | | | | |
| --- | --- | --- | --- | --- | --- | --- | --- | --- | --- | --- | --- | --- |
| Region | AP Level | Subregion | Sham Vehicle | CCI Unaffected | CCI Affected | Sham Minocycline | CCI Minocycline | Sham Vehicle | CCI Unaffected | CCI Affected | Sham Minocycline | CCI Minocycline |
| Hipp | Dorsal | DG | 0.403 ± 0.008  (*n*=6) | 0.411 ± 0.015  (*n*=6) | 0.400 ± 0.013  (*n*=6) | 0.404 ± 0.009  (*n*=6) | 0.416 ± 0.013  (*n*=6) | 0.408 ± 0.022  (*n*=6) | 0.408 ± 0.014  (*n*=6) | 0.420 ± 0.012  (*n*=6) | 0.404 ± 0.018  (*n*=6) | 0.416 ± 0.029  (*n*=6) |
|  |  | CA3 | 0.404 ± 0.010  (*n*=6) | 0.404 ± 0.011  (*n*=6) | 0.398 ± 0.011  (*n*=6) | 0.413 ± 0.020  (*n*=6) | 0.402 ± 0.026  (*n*=5) | 0.410 ± 0.014  (*n*=6) | 0.411 ± 0.017  (*n*=6) | 0.406 ± 0.021  (*n*=6) | 0.418 ± 0.016  (*n*=6) | 0.408 ± 0.008  (*n*=6) |
|  |  | CA1 | 0.397 ± 0.022  (*n*=6) | 0.416 ± 0.004  (*n*=6) | 0.408 ± 0.017  (*n*=6) | 0.420 ± 0.022  (*n*=6) | 0.400 ± 0.014  (*n*=5) | 0.405 ± 0.018  (*n*=6) | 0.404 ± 0.010  (*n*=6) | 0.411 ± 0.015  (*n*=6) | 0.404 ± 0.010  (*n*=6) | 0.412 ± 0.020  (*n*=6) |
|  | Intermediate | DG | 0.401 ± 0.020  (*n*=6) | 0.387 ± 0.009  (*n*=6) | 0.409 ± 0.013  (*n*=6) | 0.405 ± 0.024  (*n*=6) | 0.402 ± 0.021  (*n*=6) | 0.396 ± 0.014  (*n*=6) | 0.408 ± 0.009  (*n*=6) | 0.415 ± 0.017  (*n*=6) | 0.411 ± 0.018  (*n*=6) | 0.405 ± 0.012  (*n*=6) |
|  |  | CA3 | 0.401 ± 0.016  (*n*=6) | 0.406 ± 0.018  (*n*=6) | 0.407 ± 0.017  (*n*=6) | 0.406 ± 0.016  (*n*=6) | 0.409 ± 0.014  (*n*=6) | 0.413 ± 0.019  (*n*=6) | 0.406 ± 0.014  (*n*=6) | 0.407 ± 0.012  (*n*=6) | 0.396 ± 0.029  (*n*=6) | 0.398 ± 0.013  (*n*=6) |
|  |  | CA1 | 0.401 ± 0.016  (*n*=6) | 0.402 ± 0.013  (*n*=6) | 0.412 ± 0.022  (*n*=6) | 0.404 ± 0.018  (*n*=6) | 0.414 ± 0.021  (*n*=6) | 0.409 ± 0.029  (*n*=6) | 0.413 ± 0.012  (*n*=6) | 0.414 ± 0.023  (*n*=6) | 0.415 ± 0.027  (*n*=6) | 0.426 ± 0.015  (*n*=6) |
|  | Ventral | DG | 0.393 ± 0.015  (*n*=6) | 0.384 ± 0.013  (*n*=6) | 0.395 ± 0.010  (*n*=6) | 0.396 ± 0.006  (*n*=5) | 0.407 ± 0.017  (*n*=5) | 0.403 ± 0.007  (*n*=6) | 0.384 ± 0.016  (*n*=6) | 0.383 ± 0.041  (*n*=6) | 0.408 ± 0.012  (*n*=6) | 0.405 ± 0.013  (*n*=5) |
|  |  | CA3 | 0.400 ± 0.019  (*n*=6) | 0.399 ± 0.010  (*n*=6) | 0.405 ± 0.006  (*n*=6) | 0.411 ± 0.013  (*n*=5) | 0.411 ± 0.019  (*n*=5) | 0.403 ± 0.014  (*n*=6) | 0.396 ± 0.008  (*n*=6) | 0.417 ± 0.012  (*n*=5) | 0.412 ± 0.008  (*n*=6) | 0.408 ± 0.016  (*n*=5) |
|  |  | CA1 | 0.419 ± 0.012  (*n*=6) | 0.409 ± 0.017  (*n*=6) | 0.405 ± 0.016  (*n*=6) | 0.403 ± 0.016  (*n*=5) | 0.402 ± 0.018  (*n*=5) | 0.395 ± 0.014  (*n*=6) | 0.397 ± 0.008  (*n*=6) | 0.422 ± 0.022  (*n*=5) | 0.404 ± 0.015  (*n*=6) | 0.414 ± 0.015  (*n*=5) |
|  | Ventral Pole | CA3 | 0.392 ± 0.016  (*n*=6) | 0.415 ± 0.020  (*n*=6) | 0.408 ± 0.010  (*n*=6) | 0.409 ± 0.008  (*n*=6) | 0.404 ± 0.009  (*n*=6) | 0.403 ± 0.011  (*n*=5) | 0.399 ± 0.010  (*n*=6) | 0.389 ± 0.026  (*n*=6) | 0.389 ± 0.031  (*n*=5) | 0.395 ± 0.015  (*n*=6) |
|  |  | CA1 | 0.418 ± 0.014  (*n*=6) | 0.400 ± 0.009  (*n*=6) | 0.406 ± 0.018  (*n*=6) | 0.416 ± 0.024  (*n*=6) | 0.411 ± 0.014  (*n*=6) | 0.403 ± 0.011  (*n*=5) | 0.412 ± 0.014  (*n*=6) | 0.408 ± 0.023  (*n*=6) | 0.400 ± 0.017  (*n*=4) | 0.405 ± 0.006  (*n*=6) |
| mPFC | Rostral | CG | 0.345 ± 0.012  (*n*=6) | 0.336 ± 0.020  (*n*=6) | 0.350 ± 0.005  (*n*=5) | 0.341 ± 0.009  (*n*=6) | 0.335 ± 0.006  (*n*=6) | 0.344 ± 0.011  (*n*=6) | 0.344 ± 0.010  (*n*=6) | 0.340 ± 0.012  (*n*=6) | 0.343 ± 0.007  (*n*=6) | 0.348 ± 0.008  (*n*=6) |
|  |  | PL | 0.339 ± 0.011  (*n*=6) | 0.352 ± 0.013  (*n*=6) | 0.352 ± 0.011  (*n*=5) | 0.336 ± 0.008  (*n*=6) | 0.342 ± 0.012  (*n*=6) | 0.333 ± 0.017  (*n*=6) | 0.339 ± 0.021  (*n*=6) | 0.335 ± 0.012  (*n*=6) | 0.351 ± 0.014  (*n*=6) | 0.349 ± 0.013  (*n*=6) |
|  |  | IL | 0.341 ± 0.011  (*n*=5) | 0.349 ± 0.020  (*n*=6) | 0.337 ± 0.011  (*n*=6) | 0.347 ± 0.012  (*n*=6) | 0.332 ± 0.008  (*n*=6) | 0.347 ± 0.013  (*n*=6) | 0.343 ± 0.022  (*n*=6) | 0.334 ± 0.009  (*n*=6) | 0.342 ± 0.011  (*n*=6) | 0.340 ± 0.015  (*n*=6) |
|  | Mid | CG | 0.350 ± 0.013  (*n*=6) | 0.345 ± 0.010  (*n*=6) | 0.355 ± 0.012  (*n*=6) | 0.345 ± 0.010  (*n*=6) | 0.342 ± 0.019  (*n*=5) | 0.351 ± 0.012  (*n*=6) | 0.343 ± 0.013  (*n*=6) | 0.350 ± 0.014  (*n*=6) | 0.346 ± 0.012  (*n*=6) | 0.342 ± 0.013  (*n*=6) |
|  |  | PL | 0.343 ± 0.014  (*n*=6) | 0.352 ± 0.022  (*n*=6) | 0.355 ± 0.011  (*n*=6) | 0.350 ± 0.028  (*n*=6) | 0.353 ± 0.017  (*n*=6) | 0.342 ± 0.021  (*n*=6) | 0.350 ± 0.023  (*n*=6) | 0.349 ± 0.015  (*n*=6) | 0.350 ± 0.015  (*n*=6) | 0.347 ± 0.014  (*n*=6) |
|  |  | IL | 0.341 ± 0.017  (*n*=6) | 0.353 ± 0.012  (*n*=6) | 0.357 ± 0.008  (*n*=6) | 0.349 ± 0.015  (*n*=6) | 0.336 ± 0.015  (*n*=6) | 0.336 ± 0.023  (*n*=6) | 0.344 ± 0.028  (*n*=6) | 0.339 ± 0.014  (*n*=6) | 0.353 ± 0.015  (*n*=6) | 0.348 ± 0.020  (*n*=6) |
|  | Caudal | CG | 0.349 ± 0.014  (*n*=6) | 0.337 ± 0.017  (*n*=6) | 0.340 ± 0.023  (*n*=6) | 0.346 ± 0.010  (*n*=6) | 0.347 ± 0.009  (*n*=6) | 0.357 ± 0.015  (*n*=6) | 0.347 ± 0.017  (*n*=6) | 0.358 ± 0.008  (*n*=6) | 0.351 ± 0.008  (*n*=6) | 0.343 ± 0.012  (*n*=6) |
|  |  | PL | 0.352 ± 0.015  (*n*=6) | 0.348 ± 0.023  (*n*=6) | 0.351 ± 0.007  (*n*=6) | 0.348 ± 0.014  (*n*=6) | 0.345 ± 0.016  (*n*=6) | 0.356 ± 0.004  (*n*=6) | 0.339 ± 0.025  (*n*=6) | 0.355 ± 0.020  (*n*=6) | 0.339 ± 0.010  (*n*=6) | 0.338 ± 0.012  (*n*=6) |
|  |  | IL | 0.351 ± 0.016  (*n*=6) | 0.339 ± 0.021  (*n*=6) | 0.358 ± 0.021  (*n*=6) | 0.344 ± 0.018  (*n*=6) | 0.338 ± 0.015  (*n*=6) | 0.344 ± 0.012  (*n*=6) | 0.341 ± 0.018  (*n*=6) | 0.349 ± 0.017  (*n*=6) | 0.341 ± 0.007  (*n*=6) | 0.338 ± 0.021  (*n*=6) |

**Supplementary Table C6**. Mean radius values from fractal morphological analysis of IBA1+ microglia. Values represent group means ± standard deviation (SD) in pixels. The mean value from all microglia measured within the field of view was used to calculate the individual value for each animal. These values were then used to compute means and SD within experimental groups (i.e. *n* refers to biological, rather than technical, replicates). Hippocampal microglia have larger mean radius values due to higher image resolution compared to mPFC images. Results where *n* < 3 are excluded. Hipp: hippocampus; mPFC: medial prefrontal cortex; VPL: ventroposterior lateral nucleus; DG: dentate gyrus; CG: cingulate gyrus; PL: prelimbic cortex; IL: infralimbic cortex; DL: dorsolateral; VM: ventromedial; ZI: zona incerta. *: Sham vehicle vs CCI affected; $: Sham vehicle vs CCI unaffected; ^: Sham minocycline vs CCI minocycline; @: CCI unaffected vs CCI minocycline; #: CCI affected vs CCI minocycline; &: CCI unaffected vs CCI affected; +: Sham vehicle vs Sham minocycline. **P_adj_* < 0.05*; ** P_adj_* < 0.01.

|  | | | Left (Contralateral) | | | | | Right (Ipsilateral) | | | | |
| --- | --- | --- | --- | --- | --- | --- | --- | --- | --- | --- | --- | --- |
| Region | AP Level | Subregion | Sham Vehicle | CCI Unaffected | CCI Affected | Sham Minocycline | CCI Minocycline | Sham Vehicle | CCI Unaffected | CCI Affected | Sham Minocycline | CCI Minocycline |
| Hipp | Dorsal | DG | 87.9 ± 5.4  (*n*=6) | 88.7 ± 2.8  (*n*=6) | 89.5 ± 9.2  (*n*=6) | 84.0 ± 5.5  (*n*=6) | 88.8 ± 9.2  (*n*=6) | 90.3 ± 6.9  (*n*=6) | 87.8 ± 9.2  (*n*=6) | 91.5 ± 6.1  (*n*=6) | 87.6 ± 7.8  (*n*=6) | 87.4 ± 7.3  (*n*=6) |
|  |  | CA3 | 98.6 ± 4.8  (*n*=6) | 91.7 ± 9.3  (*n*=6) | 92.2 ± 7.0  (*n*=6) | 92.1 ± 5.3  (*n*=6) | 95.2 ± 6.5  (*n*=5) | 95.8 ± 5.6  (*n*=6) | 91.7 ± 6.7  (*n*=6) | 92.8 ± 8.9  (*n*=6) | 94.7 ± 8.4  (*n*=6) | 84.6 ± 6.8  (*n*=6) |
|  |  | CA1 | 86.8 ± 2.4  (*n*=6) | 90.6 ± 7.7  (*n*=6) | 90.1 ± 11.1  (*n*=6) | 87.2 ± 5.6  (*n*=6) | 89.4 ± 1.5  (*n*=5) | 89.5 ± 3.3  (*n*=6) | 87.5 ± 10.4  (*n*=6) | 92.3 ± 7.2  (*n*=6) | 87.2 ± 2.5  (*n*=6) | 87.3 ± 8.8  (*n*=6) |
|  | Intermediate | DG | 91.6 ± 6.3  (*n*=6) | 88.6 ± 6.5  (*n*=6) | 93.3 ± 8.5  (*n*=6) | 87.2 ± 10.5  (*n*=6) | 91.9 ± 8.4  (*n*=6) | 91.0 ± 6.9  (*n*=6) | 92.1 ± 6.6  (*n*=6) | 96.4 ± 10.4  (*n*=6) | 87.8 ± 9.4  (*n*=6) | 85.8 ± 2.8  (*n*=6) |
|  |  | CA3 | 89.3 ± 6.2  (*n*=6) | 88.6 ± 3.4  (*n*=6) | 91.2 ± 6.2  (*n*=6) | 88.0 ± 8.4  (*n*=6) | 89.3 ± 6.3  (*n*=6) | 91.9 ± 10.7  (*n*=6) | 88.5 ± 6.5  (*n*=6) | 91.5 ± 5.3  (*n*=6) | 86.5 ± 16.8  (*n*=6) | 83.1 ± 5.5  (*n*=6) |
|  |  | CA1 | 93.7 ± 4.3  (*n*=6) | 87.5 ± 2.6  (*n*=6) | 94.4 ± 5.6  (*n*=6) | 87.9 ± 4.4  (*n*=6) | 90.6 ± 6.1  (*n*=6) | 90.5 ± 6.2  (*n*=6) | 92.1 ± 5.3  (*n*=6) | 93.8 ± 7.4  (*n*=6) | 91.3 ± 9.0  (*n*=6) | 94.5 ± 6.9  (*n*=6) |
|  | Ventral | DG | 94.1 ± 10.2  (*n*=6) | 88.0 ± 6.1  (*n*=6) | 92.9 ± 7.0  (*n*=6) | 87.1 ± 6.2  (*n*=5) | 91.2 ± 9.9  (*n*=5) | 92.6 ± 7.8  (*n*=6) | 88.2 ± 9.4  (*n*=6) | 87.4 ± 14.1  (*n*=6) | 93.4 ± 6.9  (*n*=6) | 92.9 ± 11.6  (*n*=5) |
|  |  | CA3 | 89.6 ± 4.5  (*n*=6) | 84.9 ± 9.1  (*n*=6) | 90.6 ± 5.2  (*n*=6) | 89.1 ± 4.7  (*n*=5) | 90.8 ± 6.5  (*n*=5) | 93.6 ± 9.1  (*n*=6) | 90.9 ± 7.3  (*n*=6) | 90.9 ± 2.7  (*n*=5) | 90.8 ± 9.1  (*n*=6) | 93.4 ± 19.3  (*n*=5) |
|  |  | CA1 | 90.1 ± 8.4  (*n*=6) | 85.4 ± 5.2  (*n*=6) | 89.0 ± 6.1  (*n*=6) | 86.5 ± 6.1  (*n*=5) | 84.0 ± 13.7  (*n*=5) | 87.3 ± 7.0  (*n*=6) | 86.2 ± 9.1  (*n*=6) | 91.3 ± 6.0  (*n*=5) | 86.5 ± 7.9  (*n*=6) | 94.0 ± 4.2  (*n*=5) |
|  | Ventral Pole | CA3 | 88.3 ± 7.7  (*n*=6) | 90.9 ± 6.2  (*n*=6) | 96.5 ± 7.9  (*n*=6) | 88.5 ± 4.9  (*n*=6) | 86.6 ± 6.6  (*n*=6) | 94.2 ± 6.4  (*n*=5) | 87.7 ± 6.4  (*n*=6) | 86.5 ± 14.3  (*n*=6) | 81.6 ± 12.0  (*n*=5) | 82.9 ± 6.9  (*n*=6) |
|  |  | CA1 | 100.9 ± 10.6  (*n*=6) | 93.4 ± 6.5  (*n*=6) | 91.6 ± 6.2  (*n*=6) | 92.2 ± 6.1  (*n*=6) | 87.5 ± 5.9  (*n*=6) | 91.8 ± 5.3  (*n*=5) | 94.0 ± 8.6  (*n*=6) | 92.7 ± 5.7  (*n*=6) | 86.5 ± 6.0  (*n*=4) | 89.8 ± 2.7  (*n*=6) |
| mPFC | Rostral | CG | 30.3 ± 2.9  (*n*=6) | 28.4 ± 1.3  (*n*=6) | 33.8 ± 2.5^&&^  (*n*=5) | 30.3 ± 1.9  (*n*=6) | 31.1 ± 2.1  (*n*=6) | 31.5 ± 2.1  (*n*=6) | 31.4 ± 3.2  (*n*=6) | 33.4 ± 3.1  (*n*=6) | 30.7 ± 2.3  (*n*=6) | 33.3 ± 4.5  (*n*=6) |
|  |  | PL | 31.1 ± 1.3  (*n*=6) | 28.9 ± 1.9  (*n*=6) | 32.0 ± 2.7  (*n*=5) | 29.0 ± 2.4  (*n*=6) | 31.5 ± 2.7  (*n*=6) | 30.8 ± 3.2  (*n*=6) | 29.5 ± 3.4  (*n*=6) | 31.8 ± 3.8  (*n*=6) | 28.8 ± 1.1  (*n*=6) | 30.6 ± 1.9  (*n*=6) |
|  |  | IL | 31.0 ± 1.2  (*n*=5) | 29.5 ± 1.6  (*n*=6) | 30.9 ± 2.2  (*n*=6) | 30.3 ± 3.9  (*n*=6) | 29.9 ± 2.6  (*n*=6) | 32.2 ± 2.4  (*n*=6) | 29.1 ± 2.8  (*n*=6) | 32.4 ± 4.1  (*n*=6) | 28.0 ± 2.3  (*n*=6) | 30.7 ± 3.0  (*n*=6) |
|  | Mid | CG | 32.3 ± 2.3  (*n*=6) | 28.2 ± 1.6  (*n*=6) | 31.7 ± 3.9  (*n*=6) | 30.1 ± 1.9  (*n*=6) | 32.9 ± 2.0^@^  (*n*=5) | 33.4 ± 2.0  (*n*=6) | 31.9 ± 2.0  (*n*=6) | 33.2 ± 2.3  (*n*=6) | 30.8 ± 2.4  (*n*=6) | 33.0 ± 2.9  (*n*=6) |
|  |  | PL | 32.1 ± 3.1  (*n*=6) | 30.8 ± 2.9  (*n*=6) | 32.2 ± 3.2  (*n*=6) | 30.2 ± 2.6  (*n*=6) | 30.7 ± 2.0  (*n*=6) | 30.7 ± 2.8  (*n*=6) | 28.8 ± 1.8  (*n*=6) | 31.5 ± 4.5  (*n*=6) | 29.8 ± 1.5  (*n*=6) | 30.7 ± 2.3  (*n*=6) |
|  |  | IL | 32.3 ± 2.5  (*n*=6) | 30.9 ± 1.5  (*n*=6) | 32.2 ± 2.0  (*n*=6) | 28.4 ± 1.9  (*n*=6) | 30.1 ± 4.1  (*n*=6) | 31.4 ± 3.7  (*n*=6) | 29.6 ± 1.4  (*n*=6) | 32.0 ± 3.8  (*n*=6) | 29.3 ± 1.7  (*n*=6) | 32.1 ± 3.2  (*n*=6) |
|  | Caudal | CG | 31.3 ± 2.4  (*n*=6) | 29.6 ± 2.7  (*n*=6) | 32.1 ± 1.6  (*n*=6) | 28.7 ± 2.3  (*n*=6) | 28.7 ± 1.3  (*n*=6) | 32.7 ± 3.0  (*n*=6) | 29.9 ± 2.7  (*n*=6) | 32.2 ± 1.8  (*n*=6) | 28.4 ± 1.9^+^  (*n*=6) | 30.7 ± 2.2  (*n*=6) |
|  |  | PL | 31.1 ± 2.8  (*n*=6) | 28.0 ± 1.9  (*n*=6) | 30.5 ± 1.9  (*n*=6) | 28.5 ± 2.0  (*n*=6) | 27.6 ± 1.9  (*n*=6) | 32.0 ± 2.8  (*n*=6) | 30.9 ± 2.7  (*n*=6) | 31.5 ± 2.3  (*n*=6) | 27.2 ± 1.4^+^  (*n*=6) | 27.8 ± 2.0  (*n*=6) |
|  |  | IL | 33.2 ± 3.2  (*n*=6) | 30.4 ± 3.0  (*n*=6) | 32.1 ± 2.1  (*n*=6) | 27.4 ± 1.9^+^  (*n*=6) | 29.6 ± 4.6  (*n*=6) | 32.3 ± 2.7  (*n*=6) | 32.0 ± 3.4  (*n*=6) | 29.1 ± 2.3  (*n*=6) | 29.0 ± 2.5  (*n*=6) | 29.3 ± 3.1  (*n*=6) |

**Supplementary Table C7**. Perimeter values from fractal morphological analysis of IBA1+ microglia. Values represent group means ± standard deviation (SD) in pixels. The mean value from all microglia measured within the field of view was used to calculate the individual value for each animal. These values were then used to compute means and SD within experimental groups (i.e. *n* refers to biological, rather than technical, replicates). Hippocampal microglia have larger perimeter values due to higher image resolution compared to mPFC images. Results where *n* < 3 are excluded. Hipp: hippocampus; mPFC: medial prefrontal cortex; VPL: ventroposterior lateral nucleus; DG: dentate gyrus; CG: cingulate gyrus; PL: prelimbic cortex; IL: infralimbic cortex; DL: dorsolateral; VM: ventromedial; ZI: zona incerta. *: Sham vehicle vs CCI affected; $: Sham vehicle vs CCI unaffected; ^: Sham minocycline vs CCI minocycline; @: CCI unaffected vs CCI minocycline; #: CCI affected vs CCI minocycline; &: CCI unaffected vs CCI affected; +: Sham vehicle vs Sham minocycline. **P_adj_* < 0.05*; ** P_adj_* < 0.01.

|  | | | Left (Contralateral) | | | | | Right (Ipsilateral) | | | | |
| --- | --- | --- | --- | --- | --- | --- | --- | --- | --- | --- | --- | --- |
| Region | AP Level | Subregion | Sham Vehicle | CCI Unaffected | CCI Affected | Sham Minocycline | CCI Minocycline | Sham Vehicle | CCI Unaffected | CCI Affected | Sham Minocycline | CCI Minocycline |
| Hipp | Dorsal | DG | 512.4 ± 33.0  (*n*=6) | 519.1 ± 17.1  (*n*=6) | 524.4 ± 57.5  (*n*=6) | 489.6 ± 29.9  (*n*=6) | 519.5 ± 51.9  (*n*=6) | 526.6 ± 41.9  (*n*=6) | 512.6 ± 54.2  (*n*=6) | 538.4 ± 37.8  (*n*=6) | 514.7 ± 46.8  (*n*=6) | 510.9 ± 41.8  (*n*=6) |
|  |  | CA3 | 573.8 ± 26.6  (*n*=6) | 536.1 ± 55.3  (*n*=6) | 535.3 ± 42.5  (*n*=6) | 537.4 ± 33.3  (*n*=6) | 552.1 ± 36.5  (*n*=5) | 559.3 ± 33.0  (*n*=6) | 533.5 ± 40.0  (*n*=6) | 541.5 ± 50.9  (*n*=6) | 550.4 ± 46.5  (*n*=6) | 490.7 ± 40.9  (*n*=6) |
|  |  | CA1 | 508.3 ± 15.0  (*n*=6) | 531.0 ± 44.8  (*n*=6) | 523.8 ± 66.3  (*n*=6) | 510.5 ± 35.5  (*n*=6) | 520.1 ± 9.5  (*n*=5) | 522.1 ± 21.6  (*n*=6) | 510.8 ± 60.4  (*n*=6) | 541.2 ± 45.2  (*n*=6) | 509.7 ± 15.9  (*n*=6) | 510.3 ± 53.1  (*n*=6) |
|  | Intermediate | DG | 535.0 ± 37.6  (*n*=6) | 516.5 ± 38.0  (*n*=6) | 545.9 ± 52.1  (*n*=6) | 509.1 ± 64.7  (*n*=6) | 536.6 ± 49.6  (*n*=6) | 532.7 ± 41.7  (*n*=6) | 539.5 ± 38.2  (*n*=6) | 567.9 ± 62.8  (*n*=6) | 508.6 ± 58.1  (*n*=6) | 501.5 ± 14.7  (*n*=6) |
|  |  | CA3 | 521.4 ± 36.5  (*n*=6) | 513.5 ± 19.9  (*n*=6) | 535.6 ± 36.8  (*n*=6) | 511.3 ± 47.5  (*n*=6) | 519.7 ± 39.8  (*n*=6) | 536.4 ± 64.0  (*n*=6) | 513.1 ± 39.7  (*n*=6) | 532.6 ± 30.5  (*n*=6) | 502.5 ± 98.8  (*n*=6) | 484.5 ± 31.6  (*n*=6) |
|  |  | CA1 | 548.6 ± 25.4  (*n*=6) | 508.1 ± 14.2  (*n*=6) | 552.2 ± 36.7  (*n*=6) | 510.4 ± 27.2  (*n*=6) | 525.3 ± 36.7  (*n*=6) | 526.3 ± 38.7  (*n*=6) | 537.8 ± 30.9  (*n*=6) | 548.8 ± 45.1  (*n*=6) | 533.1 ± 50.7  (*n*=6) | 554.0 ± 40.0  (*n*=6) |
|  | Ventral | DG | 547.8 ± 58.2  (*n*=6) | 514.5 ± 36.6  (*n*=6) | 547.3 ± 42.5  (*n*=6) | 509.0 ± 33.8  (*n*=5) | 533.6 ± 61.1  (*n*=5) | 542.9 ± 44.4  (*n*=6) | 515.2 ± 55.2  (*n*=6) | 512.8 ± 86.6  (*n*=6) | 547.1 ± 38.3  (*n*=6) | 544.4 ± 72.3  (*n*=5) |
|  |  | CA3 | 521.6 ± 28.5  (*n*=6) | 494.3 ± 54.8  (*n*=6) | 527.9 ± 28.0  (*n*=6) | 522.6 ± 33.3  (*n*=5) | 530.8 ± 43.5  (*n*=5) | 546.4 ± 54.4  (*n*=6) | 531.0 ± 41.9  (*n*=6) | 532.6 ± 14.6  (*n*=5) | 532.4 ± 52.8  (*n*=6) | 545.2 ± 119.0  (*n*=5) |
|  |  | CA1 | 525.5 ± 51.2  (*n*=6) | 496.9 ± 29.9  (*n*=6) | 522.4 ± 35.8  (*n*=6) | 504.7 ± 33.1  (*n*=5) | 492.8 ± 82.2  (*n*=5) | 510.1 ± 40.0  (*n*=6) | 503.5 ± 55.4  (*n*=6) | 534.9 ± 36.0  (*n*=5) | 505.4 ± 46.8  (*n*=6) | 551.0 ± 26.2  (*n*=5) |
|  | Ventral Pole | CA3 | 517.0 ± 44.5  (*n*=6) | 531.5 ± 38.1  (*n*=6) | 564.3 ± 47.4  (*n*=6) | 517.0 ± 28.8  (*n*=6) | 508.2 ± 40.7  (*n*=6) | 552.7 ± 37.6  (*n*=5) | 509.2 ± 41.2  (*n*=6) | 502.6 ± 83.7  (*n*=6) | 471.5 ± 81.4  (*n*=5) | 487.3 ± 39.4  (*n*=6) |
|  |  | CA1 | 590.6 ± 60.9  (*n*=6) | 545.3 ± 39.9  (*n*=6) | 535.0 ± 37.5  (*n*=6) | 538.1 ± 37.5  (*n*=6) | 514.8 ± 38.0  (*n*=6) | 536.3 ± 32.6  (*n*=5) | 550.2 ± 52.8  (*n*=6) | 542.0 ± 32.5  (*n*=6) | 501.4 ± 34.9  (*n*=4) | 523.5 ± 15.7  (*n*=6) |
| mPFC | Rostral | CG | 177.3 ± 16.7  (*n*=6) | 166.2 ± 8.4  (*n*=6) | 198.4 ± 14.1^&&^  (*n*=5) | 176.6 ± 11.5  (*n*=6) | 180.8 ± 11.8  (*n*=6) | 183.8 ± 12.3  (*n*=6) | 183.6 ± 18.8  (*n*=6) | 195.2 ± 18.3  (*n*=6) | 179.5 ± 14.6  (*n*=6) | 194.5 ± 26.2  (*n*=6) |
|  |  | PL | 182.2 ± 8.0  (*n*=6) | 168.8 ± 11.7  (*n*=6) | 187.3 ± 16.6  (*n*=5) | 168.9 ± 13.9  (*n*=6) | 184.3 ± 16.1  (*n*=6) | 179.7 ± 18.6  (*n*=6) | 172.8 ± 20.6  (*n*=6) | 186.1 ± 22.9  (*n*=6) | 168.7 ± 6.6  (*n*=6) | 178.5 ± 10.8  (*n*=6) |
|  |  | IL | 181.7 ± 7.2  (*n*=5) | 173.3 ± 10.3  (*n*=6) | 181.4 ± 12.8  (*n*=6) | 177.7 ± 23.8  (*n*=6) | 175.0 ± 15.6  (*n*=6) | 188.5 ± 14.6  (*n*=6) | 170.9 ± 17.0  (*n*=6) | 190.8 ± 24.3  (*n*=6) | 163.6 ± 13.2  (*n*=6) | 180.5 ± 18.2  (*n*=6) |
|  | Mid | CG | 188.8 ± 13.4  (*n*=6) | 164.3 ± 10.1  (*n*=6) | 185.0 ± 23.5  (*n*=6) | 175.8 ± 11.2  (*n*=6) | 191.8 ± 10.6^@^  (*n*=5) | 195.4 ± 11.1  (*n*=6) | 185.8 ± 11.8  (*n*=6) | 194.3 ± 14.1  (*n*=6) | 180.3 ± 13.3  (*n*=6) | 192.8 ± 16.9  (*n*=6) |
|  |  | PL | 187.6 ± 19.4  (*n*=6) | 179.6 ± 18.0  (*n*=6) | 187.7 ± 19.1  (*n*=6) | 176.5 ± 14.8  (*n*=6) | 179.0 ± 11.7  (*n*=6) | 179.5 ± 17.0  (*n*=6) | 167.9 ± 10.6  (*n*=6) | 183.8 ± 26.4  (*n*=6) | 173.2 ± 8.9  (*n*=6) | 179.5 ± 12.6  (*n*=6) |
|  |  | IL | 189.1 ± 15.2  (*n*=5) | 180.7 ± 9.0  (*n*=6) | 188.5 ± 12.4  (*n*=6) | 168.5 ± 14.8  (*n*=6) | 175.5 ± 25.5  (*n*=6) | 184.3 ± 22.9  (*n*=6) | 173.2 ± 8.1  (*n*=6) | 187.4 ± 22.6  (*n*=6) | 170.8 ± 9.7  (*n*=6) | 188.0 ± 19.4  (*n*=6) |
|  | Caudal | CG | 182.8 ± 14.2  (*n*=6) | 171.6 ± 16.2  (*n*=6) | 187.1 ± 9.6  (*n*=6) | 166.9 ± 14.0  (*n*=6) | 166.6 ± 7.4  (*n*=6) | 190.8 ± 17.9  (*n*=6) | 174.4 ± 16.7  (*n*=6) | 187.5 ± 10.2  (*n*=6) | 165.4 ± 11.0^+^  (*n*=6) | 178.6 ± 13.4  (*n*=6) |
|  |  | PL | 182.2 ± 16.4  (*n*=6) | 162.5 ± 11.0  (*n*=6) | 177.2 ± 10.7  (*n*=6) | 165.7 ± 11.6  (*n*=6) | 160.1 ± 10.9  (*n*=6) | 186.8 ± 16.4  (*n*=6) | 180.4 ± 16.5  (*n*=6) | 183.9 ± 14.7  (*n*=6) | 157.8 ± 7.4^++^  (*n*=6) | 161.7 ± 11.9  (*n*=6) |
|  |  | IL | 194.7 ± 18.4  (*n*=6) | 178.2 ± 17.7  (*n*=6) | 187.2 ± 12.2  (*n*=6) | 159.7 ± 11.8^+^  (*n*=6) | 173.2 ± 27.8  (*n*=6) | 189.0 ± 17.1  (*n*=6) | 187.1 ± 20.0  (*n*=6) | 170.0 ± 14.3  (*n*=6) | 169.0 ± 14.6  (*n*=6) | 170.9 ± 19.1  (*n*=6) |
