# Supplementary material for: Minocycline Abrogates Individual Differences in Nerve Injury-Evoked Affective Disturbances in Male Rats and Prevents Associated Supraspinal Neuroinflammation
Source: J Neuroimmune Pharmacol. 2024 Jun 15;19(1):30. doi: 10.1007/s11481-024-10132-y (PMC11180027; doi:10.1007/s11481-024-10132-y)
Supplement: Supplementary file 5 — Supplementary Material 5 [file 11481_2024_10132_MOESM5_ESM.docx]

**Supplementary File E. Within-subjects comparisons for behavioural data pre- vs post-surgery.**

**Supplementary Table E**. Nonparametric within-subject analysis of radial maze, rotarod, and mechanical allodynia behavioural parameters over time. The within-subjects data in this table differently analyses the mixed ANOVA data depicted in Figures 3 and 4 and in the main text which focus instead on the between-subject comparison across these time points. Importantly, radial maze measures do not have a pre-surgery time point, so comparisons are between the first, middle, and last 7 days post-surgery to show changes in behaviours over the post-surgery period. Rotarod and sensory comparisons are post-surgery time points compared with the mean of pre-surgery trials. Friedman and Wilcoxon tests, and Holm multiple comparison correction were performed with the rstatix package in *R* and evaluated to *P* < 0.05.

| Behaviour | Statistical Test | Measure | Sham Vehicle | CCI Unaffected | CCI Affected | Sham Minocycline | CCI Minocycline |  | |  |  | |  | |  |
| --- | --- | --- | --- | --- | --- | --- | --- | --- | --- | --- | --- | --- | --- | --- | --- |
| Time in Central Atrium | Friedman | *n* | 9 | 30 | 7 | 6 | 21 |  | | | | | | | |
|  |  | Statistic | 0.22 | 40.1 | 8.86 | 1 | 15.0 |  |  |  |  |  |  |  |  |
|  |  | *P*-value | 0.89 | <0.0001 | 0.01 | 0.61 | 0.0006 |  |  |  |  |  |  |  |  |
|  | Wilcoxon Test (*P*-value) | Week 1 : 2 |  | 0.03 | 0.26 |  | 0.10 |  |  |  |  |  |  |  |  |
|  |  | Week 1 : 3 |  | <0.0001 | 0.012 |  | 0.002 |  |  |  |  |  |  |  |  |
|  |  | Week 2 : 3 |  | 0.03 | 0.052 |  | 0.13 |  |  |  |  |  |  |  |  |
| Stretch-Attend Postures | Friedman | *n* | 9 | 30 | 7 | 6 | 21 |  |  |  |  |  |  |  |  |
|  |  | Statistic | 3.31 | 1.69 | 4.57 | 3.74 | 12.0 |  |  |  |  |  |  |  |  |
|  |  | *P*-value | 0.19 | 0.43 | 0.10 | 0.15 | 0.002 |  |  |  |  |  |  |  |  |
|  | Wilcoxon Test (*P*-value) | Week 1 : 2 |  |  |  |  | 0.75 |  |  |  |  |  |  |  |  |
|  |  | Week 1 : 3 |  |  |  |  | 0.054 |  |  |  |  |  |  |  |  |
|  |  | Week 2 : 3 |  |  |  |  | 0.067 |  |  |  |  |  |  |  |  |
| Time in End of Arm | Friedman | *n* | 9 | 30 | 7 | 6 | 21 |  |  |  |  |  |  |  |  |
|  |  | Statistic | 4.22 | 28.5 | 2 | 1.33 | 8.67 |  |  |  |  |  |  |  |  |
|  |  | *P*-value | 0.12 | <0.0001 | 0.37 | 0.51 | 0.013 |  |  |  |  |  |  |  |  |
|  | Wilcoxon Test (*P*-value) | Week 1 : 2 |  | 0.18 |  |  | 0.85 |  |  |  |  |  |  |  |  |
|  |  | Week 1 : 3 |  | 0.013 |  |  | 0.38 |  |  |  |  |  |  |  |  |
|  |  | Week 2 : 3 |  | 0.27 |  |  | 0.85 |  |  |  |  |  |  |  |  |
| Grooming | Friedman | *n* | 9 | 30 | 7 | 6 | 21 |  |  |  |  |  |  |  |  |
|  |  | Statistic | 2 | 8.08 | 5.43 | 2.8 | 1.35 |  |  |  |  |  |  |  |  |
|  |  | *P*-value | 0.37 | 0.018 | 0.066 | 0.25 | 0.51 |  |  |  |  |  |  |  |  |
|  | Wilcoxon Test (*P*-value) | Week 1 : 2 |  | 0.27 |  |  |  |  |  |  |  |  |  |  |  |
|  |  | Week 1 : 3 |  | 0.055 |  |  |  |  |  |  |  |  |  |  |  |
|  |  | Week 2 : 3 |  | 0.28 |  |  |  |  |  |  |  |  |  |  |  |
| Nose Poke | Friedman | *n* | 9 | 30 | 7 | 6 | 21 |  |  |  |  |  |  |  |  |
|  |  | Statistic | 0.022 | 2.60 | 3.43 | 3.00 | 0.38 |  |  |  |  |  |  |  |  |
|  |  | *P*-value | 0.89 | 0.27 | 0.18 | 0.22 | 0.83 |  |  |  |  |  |  |  |  |
| Pellet Omissions | Friedman | *n* | 9 | 30 | 7 | 6 | 21 |  |  |  |  |  |  |  |  |
|  |  | Statistic | 4.24 | 8.27 | 5.81 | 1.33 | 2.67 |  |  |  |  |  |  |  |  |
|  |  | *P*-value | 0.12 | 0.016 | 0.055 | 0.51 | 0.26 |  |  |  |  |  |  |  |  |
|  | Wilcoxon Test (*P*-value) | Week 1 : 2 |  | 0.43 |  |  |  |  |  |  |  |  |  |  |  |
|  |  | Week 1 : 3 |  | 0.82 |  |  |  |  |  |  |  |  |  |  |  |
|  |  | Week 2 : 3 |  | 0.82 |  |  |  |  |  |  |  |  |  |  |  |
| Rearing | Friedman | *n* | 9 | 30 | 7 | 6 | 21 |  |  |  |  |  |  |  |  |
|  |  | Statistic | 4.22 | 1.40 | 3.71 | 3.00 | 1.81 |  |  |  |  |  |  |  |  |
|  |  | *P*-value | 0.12 | 0.50 | 0.16 | 0.22 | 0.40 |  |  |  |  |  |  |  |  |
| Reference Memory Errors | Friedman | *n* | 9 | 30 | 7 | 6 | 21 |  |  |  |  |  |  |  |  |
|  |  | Statistic | 2.89 | 11.3 | 1.14 | 4.00 | 1.24 |  |  |  |  |  |  |  |  |
|  |  | *P*-value | 0.24 | 0.0036 | 0.56 | 0.14 | 0.54 |  |  |  |  |  |  |  |  |
|  | Wilcoxon Test (*P*-value) | Week 1 : 2 |  | 0.53 |  |  |  |  |  |  |  |  |  |  |  |
|  |  | Week 1 : 3 |  | 0.48 |  |  |  |  |  |  |  |  |  |  |  |
|  |  | Week 2 : 3 |  | 0.70 |  |  |  |  |  |  |  |  |  |  |  |
| Total Time to Solve | Friedman | *n* | 9 | 30 | 7 | 6 | 21 |  |  |  |  |  |  |  |  |
|  |  | Statistic | 5.56 | 31.2 | 1.33 | 6.33 | 10.0 |  |  |  |  |  |  |  |  |
|  |  | *P*-value | 0.062 | <0.0001 | 0.51 | 0.042 | 0.0066 |  |  |  |  |  |  |  |  |
|  | Wilcoxon Test (*P*-value) | Week 1 : 2 |  | 0.018 |  | 1 | 0.23 |  |  |  |  |  |  |  |  |
|  |  | Week 1 : 3 |  | 0.003 |  | 0.72 | 0.19 |  |  |  |  |  |  |  |  |
|  |  | Week 2 : 3 |  | 0.49 |  | 1 | 0.62 |  |  |  |  |  |  |  |  |
| Working Memory Errors | Friedman | *n* | 9 | 30 | 7 | 6 | 21 |  |  |  |  |  |  |  |  |
|  |  | Statistic | 5.56 | 25.0 | 0.29 | 3.00 | 2.95 |  |  |  |  |  |  |  |  |
|  |  | *P*-value | 0.062 | <0.0001 | 0.87 | 0.22 | 0.23 |  |  |  |  |  |  |  |  |
|  | Wilcoxon Test (*P*-value) | Week 1 : 2 |  | 0.12 |  |  |  |  |  |  |  |  |  |  |  |
|  |  | Week 1 : 3 |  | 0.0050 |  |  |  |  |  |  |  |  |  |  |  |
|  |  | Week 2 : 3 |  | 0.25 |  |  |  |  |  |  |  |  |  |  |  |
| Rotarod | Friedman | *n* | 9 | 30 | 7 | 7 | 21 | **Sham Vehicle** | | **CCI Unaffected** | **CCI Affected** | | **Sham Minocycline** | | **CCI Minocycline** |
|  |  | Statistic | 3.86 | 53.8 | 17.8 | 2.36 | 36.2 |  |  |  |  |  |  |  |  |
|  |  | *P*-value | 0.57 | <0.0001 | 0.003 | 0.80 | <0.0001 |  |  |  |  |  |  |  |  |
|  | Wilcoxon Test (*P*-value, compared with pre-surgery) | Day 2 |  | 0.0001 | 0.07 |  | <0.0001 |  |  |  |  |  |  |  |  |
|  |  | Day 6 |  | 0.0003 | 0.21 |  | <0.0001 |  |  |  |  |  |  |  |  |
|  |  | Day 10 |  | 0.002 | 0.76 |  | 0.0002 |  |  |  |  |  |  |  |  |
|  |  | Day 13 |  | 0.007 | 0.61 |  | <0.0001 |  |  |  |  |  |  |  |  |
|  |  | Day 17 |  | 0.01 | 1 |  | 0.002 |  |  |  |  |  |  |  |  |
|  |  | **Left (Contralateral)** | | | | | | **Right (Ipsilateral)** | | | | | | | |
| Sensory | Friedman | *n* | 9 | 30 | 7 | 7 | 21 | 9 | 30 | | 7 | 7 | | 21 | |
|  |  | Statistic | 4.17 | 9.24 | 1.37 | 10.3 | 19.9 | 14.1 | 67.6 | | 18.7 | 4.96 | | 37.2 | |
|  |  | *P*-value | 0.52 | 0.10 | 0.93 | 0.07 | 0.001 | 0.01 | <0.0001 | | 0.002 | 0.42 | | <0.0001 | |
|  | Wilcoxon Test (*P*-value, compared with pre-surgery) | Day 4 |  |  |  |  | 1 | 1 | <0.0001 | | 0.009 |  | | <0.0001 | |
|  |  | Day 8 |  |  |  |  | 1 | 1 | <0.0001 | | 0.009 |  | | 0.002 | |
|  |  | Day 11 |  |  |  |  | 1 | 1 | <0.0001 | | 0.009 |  | | <0.0001 | |
|  |  | Day 15 |  |  |  |  | 1 | 1 | <0.0001 | | 0.009 |  | | 0.008 | |
|  |  | Day 19 |  |  |  |  | 1 | 1 | <0.0001 | | 0.01 |  | | 0.001 | |
